# PRAS40-driven pro-inflammatory macrophage polarization is required for inflammatory bowel disease

**DOI:** 10.1038/s41419-026-08931-7

**Published:** 2026-06-08

**Authors:** Chengfei Zhang, Yufei Bo, Jia Deng, Ting Zhang, Xinran Chen, Hongming Teng, Yuanyuan Luo, Lin Huang

**Affiliations:** 1 Department of Pathophysiology, College of Basic Medical Sciences, Dalian Medical University, Dalian, Liaoning P. R. China; 2 Liaoning Provincial Key Laboratory of Medical Cellular and Molecular Biology, Dalian, Liaoning P. R. China

**Keywords:** Inflammatory bowel disease, Monocytes and macrophages, Mucosal immunology

## Abstract

Inflammatory bowel diseases (IBDs) are chronic inflammatory disorders with unclear pathogenesis and limited therapeutic options. Macrophage dysfunction is central to IBD progression, yet how metabolic reprogramming governs macrophage function is poorly defined. Although the proline-rich Akt substrate of 40 kDa (PRAS40) is known to be important in colon cancer, its role in IBD is elusive. We found that PRAS40 mRNA expression was elevated in IBD patients, and its protein expression was similarly increased in IBD mouse models. Global PRAS40 deficiency alleviated IBD symptoms in mice, which was reversed by macrophage elimination. Consistently, myeloid-specific PRAS40 knockout mice recapitulated the protective phenotype in IBD mice. Metabolomic analysis revealed decreased amino acid levels in PRAS40-deficient BMDMs, with leucine and tryptophan identified as key drivers of macrophage polarization. Mechanistically, PRAS40 enhanced the phosphorylation of the transcription factor specificity protein 1 (SP1) to stabilize it through activating ERK and JNK signaling pathways. Upregulated SP1 augmented the transcription of solute carrier family 7 member 5 (SLC7A5), thereby boosting leucine and tryptophan uptake. This subsequently activated mTOR signaling, driving the pro-inflammatory polarization of macrophages. Collectively, PRAS40/SP1/SLC7A5 axis drives macrophage pro-inflammatory polarization by enhancing leucine and tryptophan uptake, thereby exacerbating IBD. These findings highlight the importance of amino acid reprogramming in macrophage polarization during IBD progression and propose targeting this axis as a potential therapeutic strategy for IBD.

## Introduction

Inflammatory bowel diseases (IBDs), encompassing ulcerative colitis (UC) and Crohn’s disease (CD), are characterized by chronic intestinal inflammation with unclear etiology and limited therapeutic options [1, 2]. Macrophages play a pivotal role in maintaining intestinal homeostasis, but their dysregulation is increasingly implicated in driving IBD pathogenesis. Aberrant macrophage responses to commensal bacteria amplify inflammation, while excessive infiltration of monocyte-derived macrophages (MDMs) in active IBD promotes their differentiation into pro-inflammatory phenotypes, releasing cytokines that exacerbate tissue damage. These macrophages further enhance Th17 cell responses and stimulate stromal cells to produce chemokines, creating a pro-inflammatory feedback loop. Targeting monocyte recruitment or cytokine production is potentially effective for IBD. However, the specific roles and mechanisms of macrophages in the pathogenesis of IBD require further in-depth investigation [2–7].

Macrophage metabolism, including glycolysis, TCA cycle, and amino acid pathways, greatly influences macrophage behavior [2, 3]. Amino acid metabolism has recently emerged as a key modulator of macrophage activation with diverse and context-dependent effects [8]. Arginine depletion is present in IBD, yet the arginine-NO pathway promotes macrophage pro-inflammatory activation, exacerbating tissue damage, while the arginine-urea pathway leads to macrophage anti-inflammatory activation, supporting tissue repair [9]. Serine drives IL-1β production by promoting glutathione synthesis and enhancing glycolysis, while its depletion was also reported to induce macrophage pro-inflammatory activation by activating IGF1-p38 axis [10, 11]. The conversion of glutamine to succinate increases in pro-inflammatory macrophages, whereas its influx to α-ketoglutarate is a cause of anti-inflammatory macrophage polarization [12, 13]. Tryptophan and its metabolites activate aryl hydrocarbon receptor (AhR) in tumor associated macrophages to induce immune suppression, while promotes macrophage pro-inflammatory activation under LPS treatment [14, 15]. Branched-chain amino acids (BCAAs), including leucine, isoleucine, and valine, enhance macrophage secretion of NO, IL-6, and TNFα, with leucine specifically activating mTORC1 to drive pro-inflammatory responses [16, 17]. Despite these advances, the roles of amino acids in macrophage activation remain incompletely understood.

The proline-rich Akt substrate of 40 kDa (PRAS40), encoded by *AKT1S1* gene, is a substrate of Akt1 and a component of mTORC1 [18–20]. It plays a significant role in promoting cancer cell survival, proliferation, stemness, autophagy, and metastasis [21–27]. While PRAS40 is known to contribute to colon cancer progression, its potential influence on IBD remains unexplored. Emerging yet limited evidence links PRAS40 to macrophages: PRAS40 phosphorylation is enhanced in pro-inflammatory macrophages induced by high glucose and oxidized low-density lipoproteins (oxLDL), and is reduced by Gaudichaudione H, a compound that mitigates both macrophage inflammation and IBD [28, 29]. These observations are compelling but correlative; the specific role of PRAS40 in macrophage activation and its consequent impact on IBD development warrant direct investigation.

In this study, we demonstrated that PRAS40 exacerbates IBD by promoting macrophage pro-inflammatory polarization. Mechanistically, PRAS40 enhances the stability of the transcription factor specificity protein 1 (SP1) to transcriptionally upregulates the expression of solute carrier family 7 member 5 (SLC7A5)/large amino acid transporter 1 (LAT1), thereby increasing the uptake of leucine and tryptophan to activate mTOR in macrophages. Our findings highlight the potential of targeting the PRAS40/SP1/SLC7A5 axis as a therapeutic strategy for IBD.

## Materials and methods

### Mice

All experiments involving animals were conducted according to the ethical policies and procedures approved by the Animal Care and Ethics Committee of Dalian Medical University (AEE22010), and followed ARRIVE guidelines. Mice were housed in a temperature- and humidity-controlled environment with a 12-hour light/dark cycle at the Specific Pathogen Free Experimental Animal Center of Dalian Medical University.

The *Akt1s1* global knockout mouse model was generated using the CRISPR/Cas9 system by Beijing Biocytogen (Beijing, China). Exons 2-5 at the EGE-HYT-006 locus in the mouse genome were deleted, and the deletion was confirmed by PCR product sequencing. Genotyping of the *Akt1s1* global knockout mice was performed using primers MSD-F and MSD-R for wild-type alleles and primers MSD-F and MSD-R1 for mutant alleles. The *Lyz2 Cre* mice were purchased from GemPharmatech (Jiangsu, China), and the *Akt1s1*^*f/f*^ mice were obtained from Beijing Biocytogen. The *Akt1s1*^*f/f*^ mice were crossed with the *Lyz2 Cre* mice to generate the *Akt1s1*^*f/f*^*; Lyz2 Cre* mice. Genotyping of the *Akt1s1*^*f/f*^*; Lyz2 Cre* mice was confirmed by PCR using primers Cre-F and Cre-R for *Lyz2 Cre* and Flox-F and Flox-R for *Akt1s1*^*f/f*^ (Suppl. Table 1).

### Antibodies and reagents

The following antibodies were used: PRAS40, p-S6, S6 (Cell Signaling Technology); CD80, SLC7A5, α-Tubulin, iNOS, SP1, ERK1/2, p-ERK1/2, JNK, p-JNK (Proteintech); anti-phosphoserine/threonine (Abcam); and CD206 (APC), F4/80 (Alexa Fluor™ 700), CD11b (PerCP-Cyanine5.5), CD80 (FITC) (Thermo Fisher Scientific). Dextran sodium sulfate (DSS) was purchased from MP Biomedicals; DB07268, ASN007, phorbol 12-myristate 13-acetate (PMA), and MG132 were obtained from TOPSCIENCE; Polybrene, lipopolysaccharides (LPS), and chloroquine (CQ) were from Sigma-Aldrich; IFN-γ were from MedChemExpress; and PBS liposome and clodronate liposome were from YEASEN.

### Hematoxylin and eosin (H&E) staining

Colorectal tissues were fixed in 70% ethanol for 24 h, dehydrated, and embedded in paraffin. Serial 4 μm sections were deparaffinized, rehydrated, and stained with hematoxylin for 13 min, followed by eosin for 3 min.

### Immunohistochemical staining

Tissue sections were deparaffinized, rehydrated, and treated with 3% hydrogen peroxide for 30 min. Antigen retrieval was performed in citric acid buffer using a pressure cooker. Sections were incubated with primary antibodies overnight at 4 °C, followed by HRP-conjugated secondary antibodies for 1 h at room temperature. Signals were visualized using a diaminobenzidine substrate kit, and slides were counterstained with hematoxylin.

### Flow cytometry analysis

Cells were incubated with fluorescent-labeled antibodies for 60 min at room temperature. After washing, cells were resuspended in PBS and analyzed using an Accuri C6 Plus flow cytometer. Data acquisition was performed using software FlowJo.

### Quantitative reverse transcription PCR (qRT-PCR)

Total RNA was extracted using Trizol (Thermo Fisher Scientific) and reverse-transcribed into cDNA. qRT-PCR was performed using SYBR Green PCR Master Mix (Sevenbio, China) on a real-time PCR system. Gene expression levels were normalized to β-actin. Primer sequences are listed in Suppl. Tables 2 and 3.

### Western blotting analysis

Protein extracted from the cells in lysis buffer (50 mM Tris (pH 7.4), 150 mM NaCl, 20 mM Na_2_HPO_4_/NaH_2_PO_4_, 10% glycerol, 1% Triton X-100, complete protease inhibitors and phosphatase inhibitors (Thermo)) was electrophoresed by SDS-polyacrylamide gels and was transferred to poly vinylidene difuoride (PVDF) membranes. Primary antibody was used at 4 °C overnight. Incubate the secondary antibody at room temperature for one hour. Blots were visualized with SuperSignal^TM^ West Pico PLUS Stable Peroxied.

### DSS-induced colitis model

Six- to eight week-old male mice were administered 2.0% DSS in drinking water for 6–7 days, followed by a 1–2-day recovery period with regular water. DAI was used to evaluate the grade and extent of intestinal inflammation according to the data of weight loss (0–4), stool consistency (0–3), and rectal bleeding (0–3). Body weights were recorded daily to calculate weight change percentages and colon lengths were measured at the end of the experiment [30].

### Depletion of macrophages in mice

Systemic depletion of macrophages in mice was conducted as previous described with minor modifications [31]. Briefly, six to eight week-old male mice were randomly divided into clodronate liposome and control groups. Clodronate liposomes (200 μl/25 g body weight) were intraperitoneal injected on days −2, 0, 2, and 4 relative to DSS treatment initiation. Same volume of PBS liposomes was used as the vehicle control.

### Cell lines and cell culture

The human embryonic kidney cell line HEK293T, human monocytic leukemia cell line THP-1, and mouse macrophage cell line RAW264.7 were obtained from the Cell Resource Center, Peking Union Medical College. All cell lines were authenticated by short tandem repeat (STR) profiling and tested negative for mycoplasma contamination. HEK293T and RAW264.7 cells were cultured in Dulbecco’s Modified Eagle’s Medium (DMEM) supplemented with 10% fetal bovine serum (FBS). THP-1 cells were maintained in RPMI-1640 medium supplemented with 10% FBS and 0.05 mM β-mercaptoethanol. All cell lines were incubated at 37 °C in a humidified atmosphere with 5% CO_2_.

### Lentivirus production and transduction

Lentiviral particles were produced by co-transfecting HEK293T cells with the packaging plasmid psPAX2 (Addgene plasmid #12260) and the envelope plasmid pMD2.G (Addgene plasmid #12259). Virus-containing supernatants were harvested 48 hours post-transfection. Target cells were infected with lentivirus in the presence of 8 μg/mL Polybrene.

### Bone marrow-derived macrophage (BMDM) isolation and in vitro polarization

BMDMs were isolated from the femurs and tibias of 6- to 8-week-old mice. After erythrocyte lysis, bone marrow cells were cultured in BMDM differentiation medium (high-glucose DMEM, 10% FBS, and 20% L929-conditioned supernatant) for 7 days, with fresh medium replenished every 2–3 days. On day 7, differentiated BMDMs were harvested and plated in six-well plates. For polarization, BMDMs were stimulated with 100 ng/mL LPS for 24 h, followed by RNA or protein extraction [5].

### Isolation of colon lamina propria mononuclear Cells (cLPMCs)

Colon tissues were washed in PBS, cut into 1 × 1 mm pieces, and incubated twice in PBS containing 5 mM EDTA and 1 mM DTT at 37 °C for 25 min. Tissues were further digested in RPMI-1640 medium containing 2 mg/mL collagenase IV and 0.1 mg/mL DNase I at 37 °C with shaking. cLPMCs were isolated by Percoll gradient centrifugation (40% and 80%) and collected from the interface [5].

### Isolation and culture of intestinal epithelial cells (IECs)

IECs were isolated from wild-type mice. Tissues were cut into 1 × 1 mm pieces and digested in RPMI-1640 medium containing 0.5% FBS, 1 mg/mL collagenase IV, and 0.08 mg/mL DNase I at 37 °C for 45 min. Cell pellets were resuspended in PBS, and the 75 μm fraction was cultured in complete DMEM/F12 medium [5].

### Targeted metabolomics analysis

Metabolic profiling was performed using the Q300 Metabolite Array Kit (Metabo-Profile, Shanghai, China). The targeted metabolomic panel comprises 300 metabolites, including fatty acids, amino acids, organic acids, benzenoids and carbohydrates, etc. The standards of all targeted metabolites were obtained from Sigma-Aldrich (St. Louis, MO, USA) and TRC Chemicals (Toronto, ON, Canada). In brief, each sample mixed with internal standards was detected by an ultra-performance liquid chromatography coupled to a tandem mass spectrometry system (UPLC-MS/MS, ACQUITY UPLC-Xevo TQ-S, Waters Corp., Milford, MA, USA). The raw UPLC-MS/MS data were processed using the TMBQ software (v1.0, Metabo-Profile, Shanghai, China) to perform peak integration, calibration, and quantification for each metabolite. The platform iMAP (v1.0, Metabo-Profile, Shanghai, China) was used for statistical analyses, including PCA, OPLS-DA, univariate analysis and pathway analysis, et al.

### Analysis of leucine and tryptophan levels

Cells supernatant and lysates were subjected to ELISA kit of leucine (MM-60296H1, MEIMIAN, China) and tryptophan (MM-51157H1, MEIMIAN, China) following the manufacturer’s instructions. After the reaction, the value was read at 450 nm and the relative amount was calculated [32].

### Luciferase reporter assay

The promoter fragment (−2000 bp-+100 bp) of SLC7A5 was cloned into the pGL3-Basic vector. Cells were co-transfected with the pGL3-promoter plasmid, a pRL-SV40 Renilla luciferase reporter plasmid, and an expression vector encoding the indicated transcription factors or a control vector. Firefly and Renilla luciferase activities were measured, and firefly luciferase activity was normalized to Renilla luciferase activity.

### Chromatin immunoprecipitation (ChIP)

Cells were cross-linked with 1% formaldehyde, and the reaction was quenched with glycine. Chromatin was sonicated and immunoprecipitated with specific antibodies. Rabbit IgG served as a negative control. Precipitated DNA was analyzed by qPCR using primers listed in Suppl. Table 4.

### Co-immunoprecipitation (Co-IP)

Cell lysates were incubated with anti-FLAG M2 Affinity Gel (Sigma) or anti-SP1 antibody coupled to protein A agarose (GE) at 4 °C overnight. Immunoprecipitates were analyzed by Western blotting.

### Statistical analysis

The analyses of differentially expressed genes were performed in GEO datasets: GSE38713 for comparing UC and normal colon tissues, and GSE10220 for comparing peripheral blood mononuclear cells (PBMCs) and monocyte-derived macrophages (MDMs). Data are presented as mean ± standard deviation (SD). For experiments with more than three groups, one-way ANOVA was used. Differences between two groups were assessed using a two-sided Student’s t-test. All in vitro experiments were performed in triplicate. Statistical analyses were conducted using GraphPad Prism 8 software, and a *P*-value < 0.05 was considered statistically significant.

## Results

### PRAS40 is critical to IBD pathogenesis

**Fig. 1:**
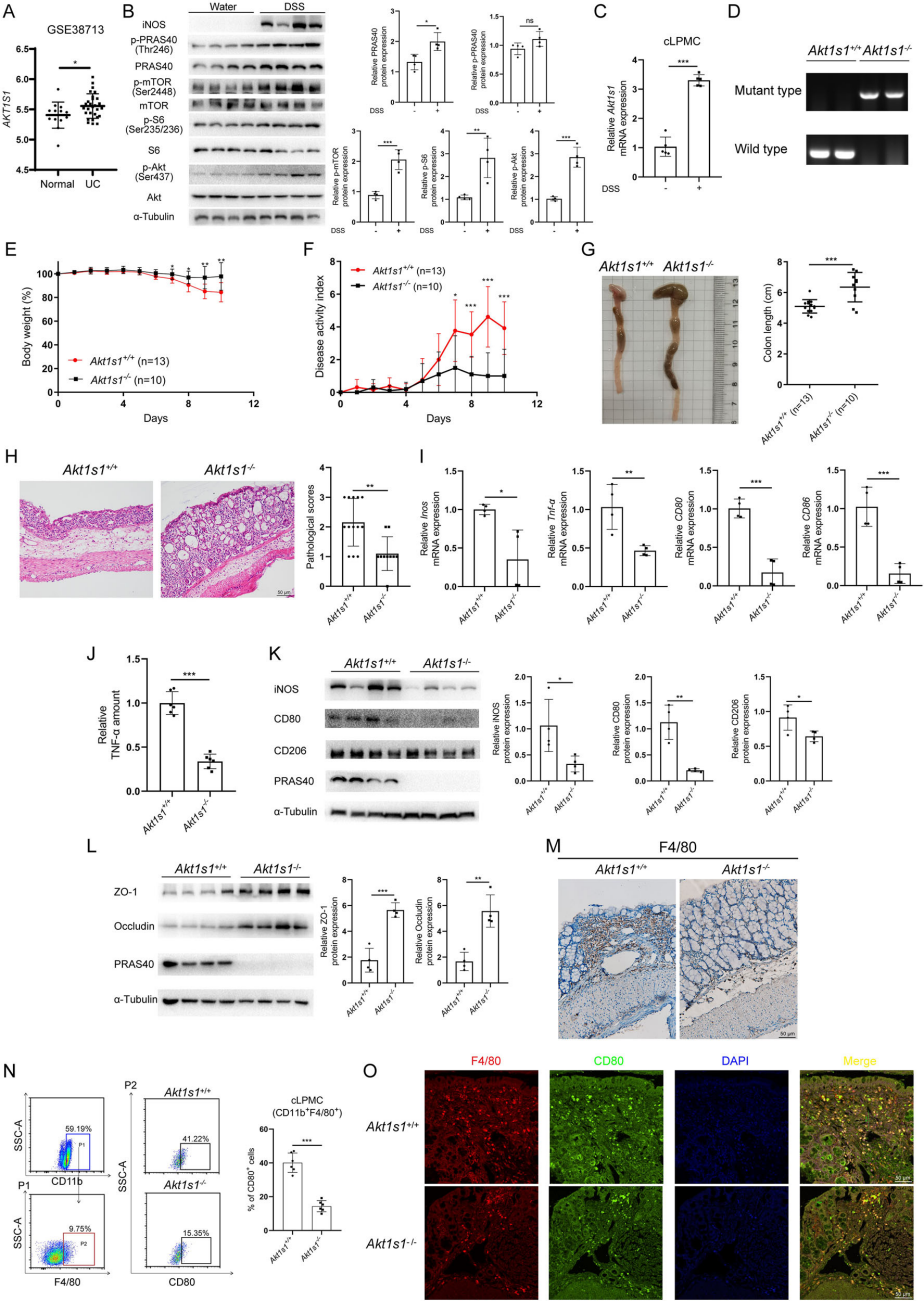
Expression of PRAS40 in IBD and effects of PRAS40 globally depletion on DSS-induced colitis. **A** Expression of *AKT1S1* mRNA in UC samples from the GSE38713 dataset. **B** Western blotting analyses of colon tissues from control or DSS-treated mice. **C** qPCR analyses of cLPMCs from control or DSS-treated mice (*n* = 5). **D**−**O** DSS-induced colitis in *Akt1s1*^*+/+*^ (*n* = 13) and *Akt1s1*^*-/-*^ mice (*n* = 10). Genotyping results (**D**), body weights (**E**), disease activity index (DAI)(F), colon lengths (**G**), H&E staining of colon tissues and corresponding pathological scores (**H**), qPCR analyses of colon tissues (*n* = 4) (**I**), ELISA analyses (*n* = 6) (**J**), western blotting analyses of colon tissues (*n* = 4) (**K**, **L**), IHC staining with anti-F4/80 antibody (**M**), flow cytometry analyses of mouse cLPMCs using anti-F4/80, anti-CD11b and anti-CD80 antibodies (*n* = 6) (**N**) and immunofluorescent staining of colon tissues with anti-F4/80 and anti-CD80 antibodies (**O**). The quantification of the band density was graphed. Phosphorylation bands were normalized to their total protein bands, and unphosphorylation bands were normalized to α-Tubulin. ns not significant. Data represent the mean ± SD. *P < 0.05; **P < 0.01; ***P < 0.001.

To investigate the role of PRAS40 in IBD, we analyzed microarray data from UC patients (GSE38713) in The Gene Expression Omnibus database (GEO). The mRNA levels of *AKT1S1* which encodes PRAS40 were significantly higher in intestinal tissues of UC patients compared to healthy controls (Fig. 1A). Consistently, both PRAS40 expression and its phosphorylation were markedly upregulated in colon tissues of DSS-induced colitis mice, whereas the p-PRAS40/PRAS40 ratio remained unchanged (Fig. 1B, Suppl. Fig. 8A). Furthermore, PRAS40 upregulation was specific to colon lamina propria mononuclear cells (cLPMCs) and not observed in intestinal epithelial cells (IECs), highlighting a potential crucial role for PRAS40 in cLPMCs (Fig. 1C, Suppl. Fig. 1A).

To further explore the impact of PRAS40 on IBD-relevant model of colitis, we generated PRAS40 global knockout mice (*Akt1s1*^-/-^)(Fig. 1D). PRAS40 deletion notably alleviated colitis, as demonstrated by attenuated body weight loss, decelerated disease activity index (DAI) progression, reduced colon shortening, less severe epithelium damage at the tissue level, and decreased iNOS and TNFα (Fig. 1E–K, Suppl. Fig. 8B). Additionally, the expression of epithelial tight junction markers ZO-1 (Zo-1) and Occludin (Ocln) was much higher in DSS-treated *Akt1s1*^-/-^ mice compared to *Akt1s1*^+/+^ mice (Fig. 1L, Suppl. Fig. 2A, Suppl. Fig. 8C). Collectively, these data suggest that PRAS40 plays a critical role in IBD.

### PRAS40 promotes IBD by increasing pro-inflammatory macrophages

To further clarify the role of PRAS40 in IBD, we constructed intestinal epithelium-specific *Akt1s1* knockout mice (*Akt1s1*^*f/f*^*; Villin Cre*). Consistent with the lack of PRAS40 upregulation in IECs during colitis, these mice exhibited colitis phenotypes indistinguishable from controls (Suppl. Fig. 1B–G, Suppl. Fig. 8D), indicating that epithelial PRAS40 is dispensable. We therefore focused on cLPMCs, where PRAS40 is specifically upregulated. Unlike neutrophils (Ly6G^+^), infiltration of macrophages (F4/80^+^) was prominent in the inflamed intestines, and this macrophage infiltration was reduced in *Akt1s1*^-/-^ mice compared to *Akt1s1*^+/+^ mice (Fig. 1M, Suppl. Fig. 2B). These results suggest that PRAS40 likely promotes IBD by regulating macrophage function.

We next found that the expression of pro-inflammatory macrophage markers (CD80, CD86, iNOS, and TNFα) was markedly decreased in the colon tissues of *Akt1s1*^-/-^ mice, while anti-inflammatory macrophage markers (CD206, IL-10, and Arginase 1) remained unchanged (Fig. 1I, K, Suppl. Fig. 2C). Furthermore, the proportion of pro-inflammatory macrophages (CD11b^+^F4/80^+^CD80^+^) in cLPMCs was greatly lower in DSS-treated *Akt1s1*^-/-^ mice compared to *Akt1s1*^+/+^ mice (Fig. 1N). Immunofluorescence staining confirmed fewer F4/80^+^CD80^+^ cells in colon tissues of *Akt1s1*^-/-^ mice (Fig. 1O). Although the proportion of anti-inflammatory macrophage (CD11b^+^F4/80^+^CD206^+^) showed a statistically significant increase in *Akt1s1*^-/-^ mice, their much lower abundance relative to pro-inflammatory macrophages suggests a limited impact on intestinal inflammation (Suppl. Fig. 2D). Thus, PRAS40 increases pro-inflammatory macrophages in IBD.

**Fig. 2:**
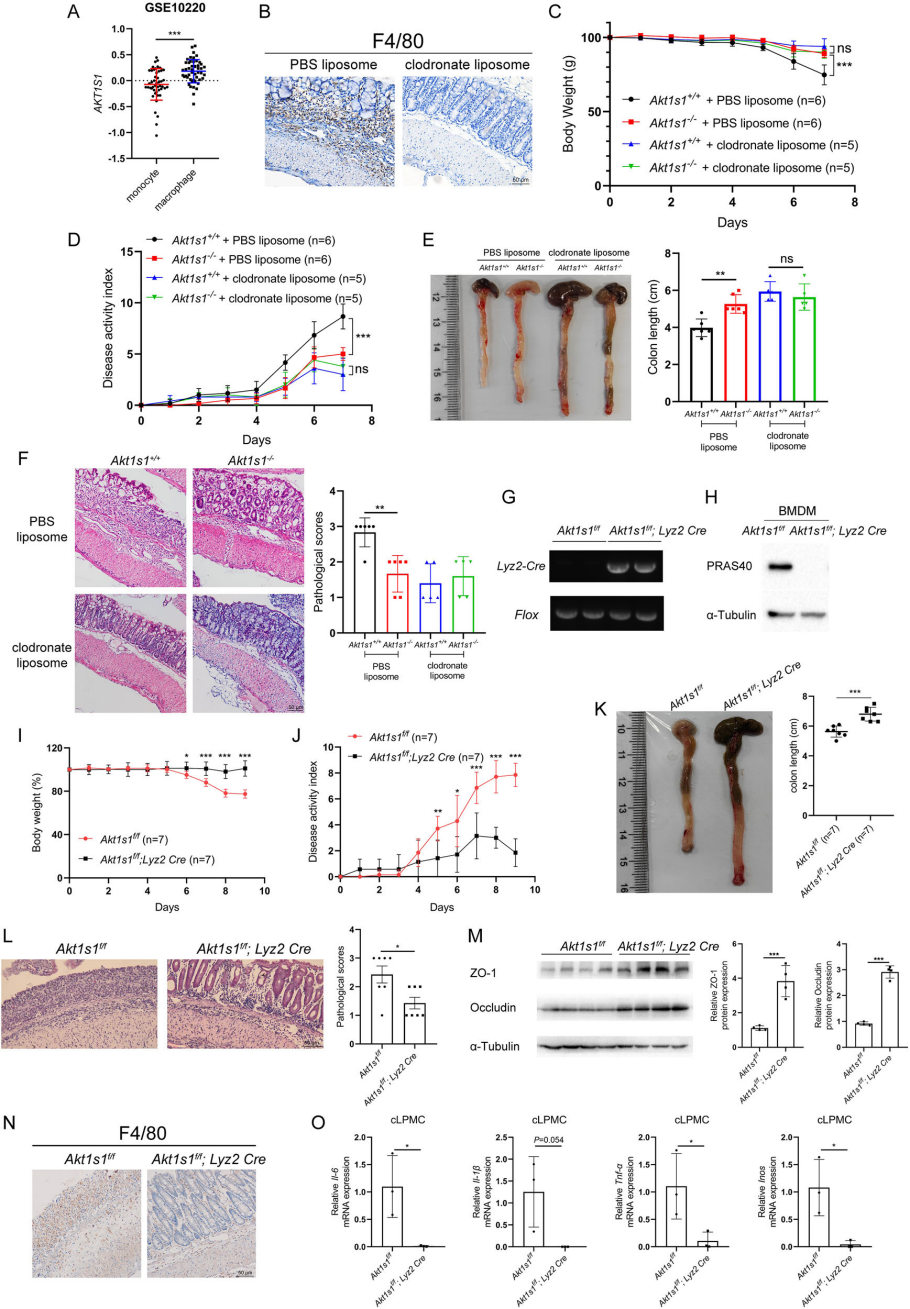
Roles of macrophages in PRAS40-driven colitis. **A** Expression of *AKT1S1* mRNA in human monocytes and monocyte-derived macrophages from the GSE10220 dataset. **B**–**F** DSS-induced colitis in *Akt1s1*^*+/+*^ and *Akt1s1*^*-/-*^ mice treated with control (*n* = 6) or clodronate liposomes (*n* = 5). IHC staining with anti-F4/80 antibody (**B**), body weights (**C**), DAI (**D**), colon length (**E**) and H&E staining and pathological scores (**F**). **G**–**O** DSS-induced colitis in *Akt1s1*^*f/f*^ and *Akt1s1*^*f/f*^*; Lyz2 Cre* mice (*n* = 7). Genotyping results (**G**), western blotting analyses in mouse BMDMs (**H**), body weights (**I**), DAI (**J**), colon lengths (**K**), H&E staining and corresponding pathological scores (**L**), western blotting analyses of colon tissues. The quantification of the band density normalized to α-Tubulin was graphed (**M**), IHC staining with anti-F4/80 antibody (**N**) and qPCR analyses of mouse cLPMCs (*n* = 3) (**O**). Data represent the mean ± SD. *P < 0.05; **P < 0.01; ***P < 0.001.

RNA-seq data (GSE10220) revealed an increase of *AKT1S1* mRNA level in monocyte-derived macrophages compared to human monocytes (Fig. 2A). To determine the necessity of macrophages in PRAS40-driven IBD, we eliminated macrophages in mice using clodronate liposome (Fig. 2B). Depletion of macrophages alleviated the colitis phenotypes in *Akt1s1*^+/+^ but not *Akt1s1*^-/-^ mice (Fig. 2C–F). We next generated myeloid-specific *Akt1s1* knockout mice (*Akt1s1*^*f/f*^*; Lyz2 Cre*) (Fig. 2G, H, Suppl. Fig. 3A, Suppl. Fig. 8E, F). Colitis phenotypes were significantly mitigated in DSS-treated *Akt1s1*^*f/f*^*; Lyz2 Cre* mice compared to *Akt1s1*^*fl/fl*^ mice (Fig. 2I–M, Suppl. Fig. 3B, Suppl. Fig. 8G). Reduced macrophage filtration and decreased pro-inflammatory cytokine expression in cLPMCs were observed in *Akt1s1*^*f/f*^*; Lyz2 Cre* mice, while anti-inflammatory cytokine levels remained unchanged (Fig. 2N, O, Suppl. Fig. 3C). These findings suggest that pro-inflammatory macrophages are essential for PRAS40-driven IBD.

### PRAS40 orchestrates the pro-inflammatory polarization of macrophages

**Fig. 3:**
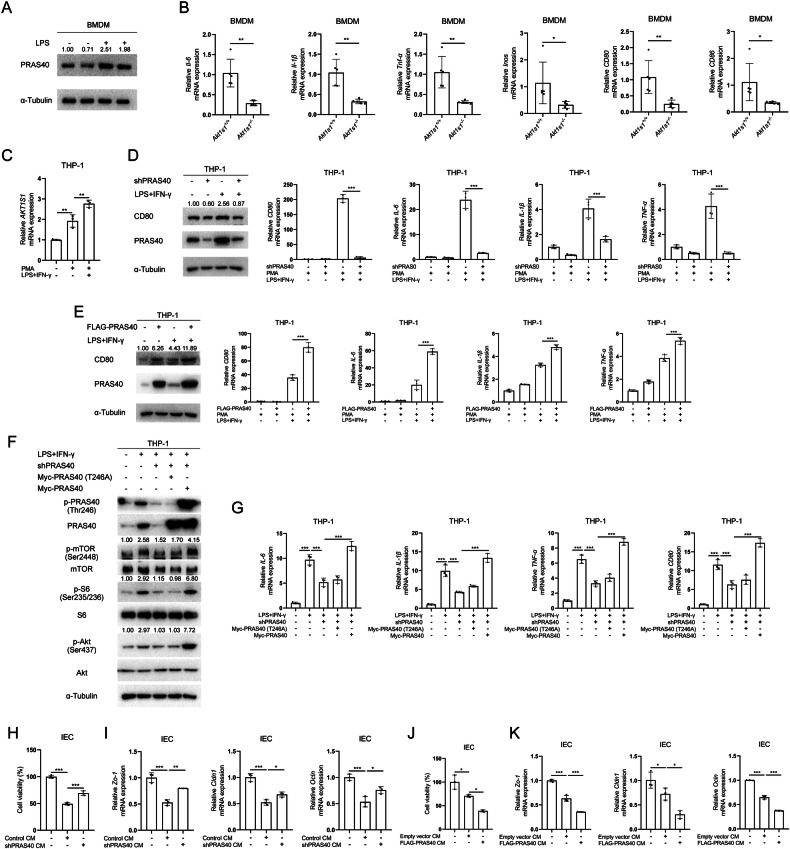
Promotion of PRAS40 on the pro-inflammatory polarization of macrophages. **A**, B, mouse BMDMs were treated with or without LPS. Western blotting analyses (**A**) and qPCR analyses (*n* = 5) (**B**). **C** qPCR analyses of THP-1 cells treated with or without LPS and IFN-γ. **D** THP-1 cells introduced with control or PRAS40 shRNA, followed by western blotting and qPCR analyses. **E** THP-1 cells introduced with empty vector or FLAG-PRAS40 expression vector, followed by western blotting and qPCR analyses. **F**, **G** THP-1 cells introduced with control or PRAS40 shRNA, together with or without Myc-PRAS40 T246A or Myc-PRAS40, followed by western blotting and qPCR analyses. **H**, **I** Mouse IECs treated with CM from control or shPRAS40 transfected RAW264.7 cells. Cell viability (**H**) and qPCR analyses (**I**). **J**, **K** Mouse IECs treated with CM from empty vector or FLAG-PRAS40 transfected RAW264.7 cells. Cell viability (**J**) and qPCR analyses (**K**). Data represent the mean ± SD. *P < 0.05; **P < 0.01; ***P < 0.001.

To further elucidate the role of PRAS40 in macrophage polarization, we conducted in vitro experiments. PRAS40 expression was upregulated in LPS-treated bone marrow-derived macrophages (BMDMs)(Fig. 3A, Suppl. Fig. 8H). While the viability of BMDMs from *Akt1s1*^-/-^ mice was not different from that of *Akt1s1*^+/+^ mice, the expression of pro-inflammatory macrophage markers was lower in PRAS40-deficient BMDMs upon LPS stimulation (Fig. 3B, Suppl. Fig. 4A). Consistently, PRAS40 expression was elevated during pro-inflammatory polarization in THP-1 cells (Fig. 3C) (If not indicated, THP-1 cells and RAW264.7 cells were treated with LPS and IFN-γ). PRAS40 deletion downregulated pro-inflammatory macrophage markers, while PRAS40 overexpression upregulated their expression (Fig. 3D, E, Suppl. Fig. 4B, C, Suppl. Fig. 8I, J). We further employed a PRAS40 T246 phosphorylation dead mutant (Myc-PRAS40 T246A)[20, 22], and found that wild type but not mutant type PRAS40 increased the expression of pro-inflammatory macrophage markers (Fig. 3F, G, Suppl. Fig. 8K). Therefore, PRAS40 promotes the pro-inflammatory polarization of macrophages in a phosphorylation-dependent manner.

To investigate the functional impact of PRAS40-polarized macrophages on IECs, we prepared conditioned medium (CM) from the culture supernatant of pro-inflammatory macrophages. Treatment of primary mouse IECs with control macrophage-derived CM suppressed IEC viability and the expression of epithelial tight junction markers, while PRAS40-deficient macrophage-derived CM showed less suppression (Supplementary Fig. 4D, Fig. 3H, I). Conversely, CM from PRAS40-overexpressing macrophages exacerbated these detrimental effects (Fig. 3J, K). These data suggest that PRAS40-driven pro-inflammatory macrophage responses directly impair the integrity and function of epithelial cells.

### PRAS40 enhances leucine and tryptophan uptake to drive macrophage pro-inflammatory polarization

**Fig. 4:**
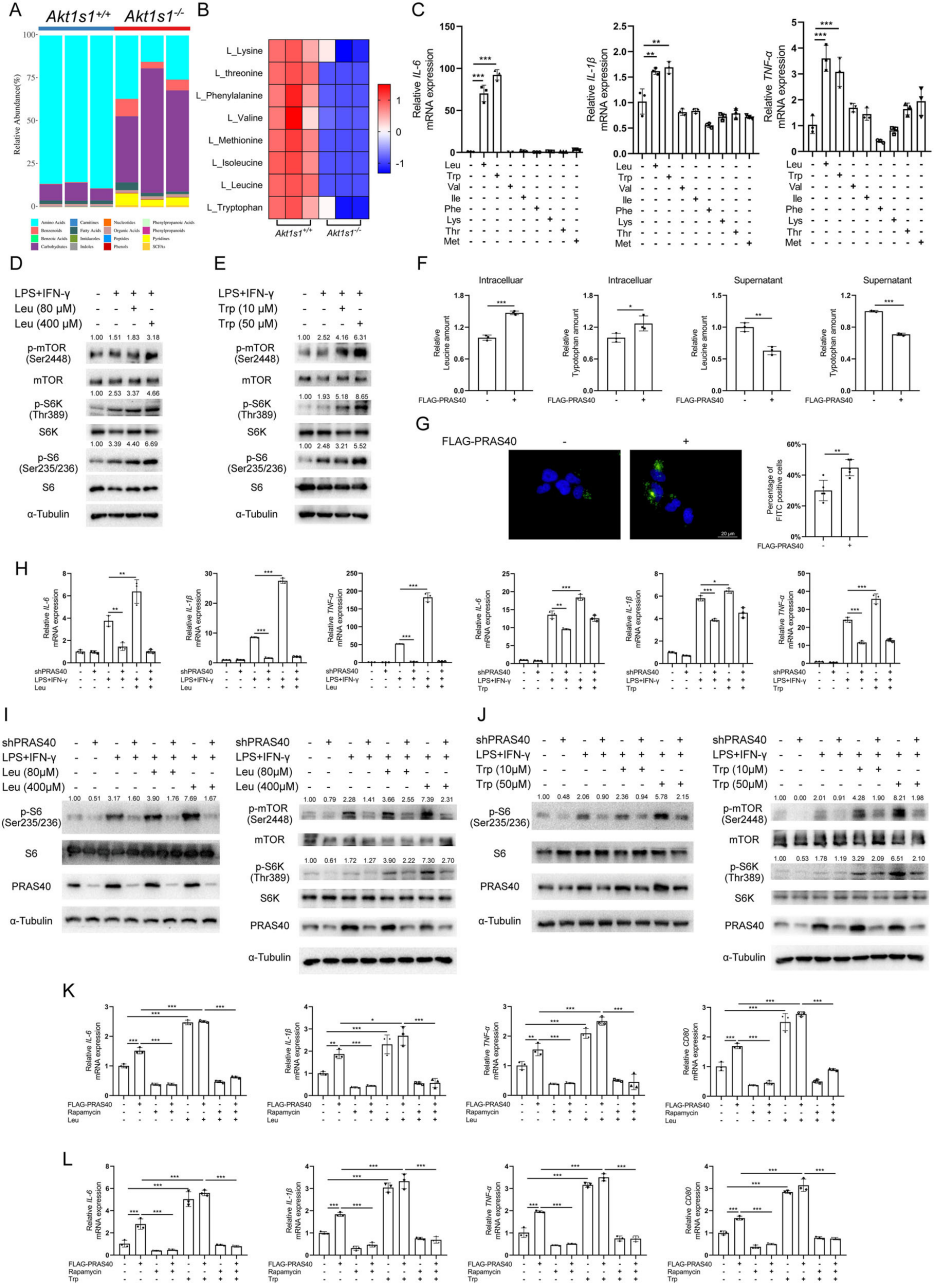
Effects of PRAS40 on amino acid metabolism during pro-inflammatory polarization of macrophages. **A**, **B** Metabolomics analyses of LPS-treated *Akt1s1*^*+/+*^ and *Akt1s1*^*-/-*^ BMDMs. Metabolite distribution (**A**) and heatmap of essential amino acids (**B**). **C** qPCR analyses of THP-1 cells treated with Leu (0.8 mM), Trp (80 μM), Val (0.8 mM), Ile (0.8 mM), Phe (0.4 mM), Lys (0.8 mM), Thr (0.8 mM) or Met (0.2 mM). **D**, **E** western blotting analyses of THP-1 cells treated with Leu (**D**) or Trp (**E**). **F** ELISA analyses of leucine and tryptophan levels in empty vector- or PRAS40-transfected THP-1 cells. **G** The indicated cells were treated with FITC-leucine at 500 μM for 16 h after 4 h preincubation in EBSS (Earle’s balanced salt solution), followed by fluorescence imaging. The quantification of FITC-positive cells was shown in the right panel. **H**–**J** THP-1 cells introduced with control or PRAS40 shRNA, with or without treatment of leucine (2 mM) or tryptophan (50 μM), followed by qPCR (**H**) and western blotting analyses (**I**, **J**). **K**, **L** THP-1 cells transfected with empty vector or PRAS40 expression vector, and treated with Leu (2 mM) or Trp (100 μM), or together with rapamycin (100 nM), followed by qPCR analyses. The quantification values of the band density were labeled above the bands. Phosphorylation bands were normalized to their total protein bands, and unphosphorylation bands were normalized to α-Tubulin. Data represent the mean ± SD. *P < 0.05; **P < 0.01; ***P < 0.001.

Metabolism reprogramming is a key driver of macrophage polarization [2, 3, 8]. To explore the mechanism by which PRAS40 promotes macrophage pro-inflammatory polarization, we screened key metabolites in LPS-treated BMDMs. PRAS40 deletion notably reduced overall amino acid levels, particularly essential amino acids (Fig. 4A-B). Among these essential amino acids, only leucine and tryptophan consistently enhanced pro-inflammatory cytokine expression in macrophages, which required the presence of LPS (Fig. 4C), as neither amino acid alone was sufficient to induce the expression of pro-inflammatory cytokines (Supplementary Fig. 5). In IBD mouse models, mTOR activation was upregulated, paralleling the upregulation of PRAS40 (Fig. 1B, Supplementary Fig. 8A). Identical to the established role of mTOR in driving macrophage pro-inflammatory polarization, mTOR activation was induced by leucine or tryptophan treatment, as demonstrated by the upregulated phosphorylation of mTOR and its downstream targets S6K, S6 (Fig. 4D, E, Supplementary Fig. 8L, M). Furthermore, PRAS40 overexpression increased the intracellular levels of both leucine and tryptophan in pro-inflammatory macrophages while decreasing their concentrations in the culture media, suggesting enhanced uptake of both essential amino acids (Fig. 4F). This was further supported by increased FITC-labeled leucine uptake in PRAS40-overexpressing cells (Fig. 4G). Notably, supplementation of leucine or tryptophan failed to restore pro-inflammatory cytokine expression or activate mTOR in PRAS40-depleted macrophages, different from control macrophages (Fig. 4H–J, Supplementary Fig. 8N, O). Treatment with mTOR inhibitor rapamycin completely abolished the increase in pro-inflammatory cytokines induced by PRAS40 overexpression and supplementation of leucine or tryptophan (Fig. 4K, L). Therefore, PRAS40 activates mTOR by increasing cellular uptake of leucine and tryptophan, thereby inducing macrophage inflammatory polarization.

### PRAS40 upregulates SLC7A5 expression to increase leucine and tryptophan uptake in macrophages

**Fig. 5:**
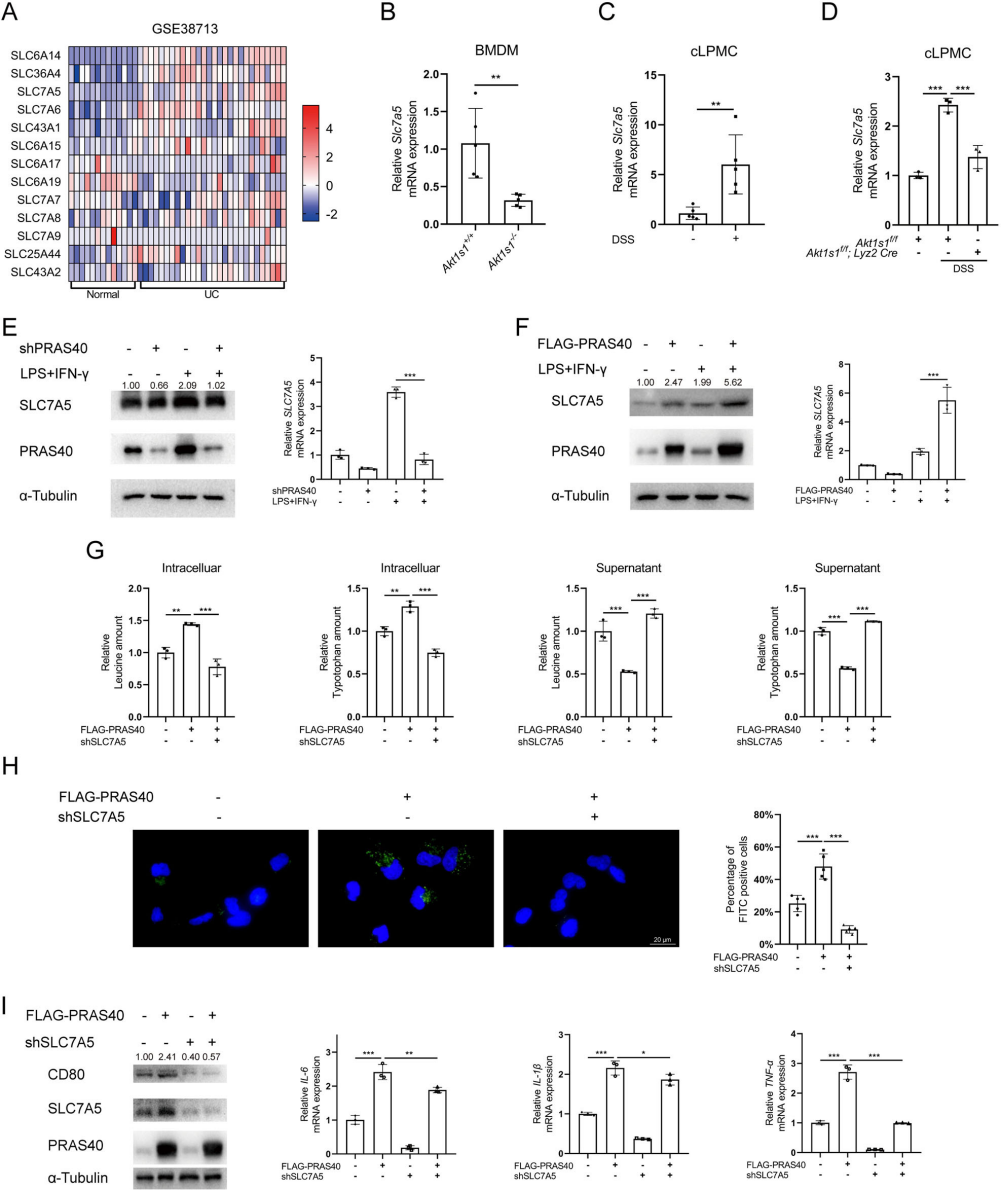
Effects of SLC7A5 on the PRAS40-promoted leucine and tryptophan uptake during pro-inflammatory polarization of macrophages. **A** mRNA expression of leucine and tryptophan transporters in control or UC samples from the GSE38713 dataset. **B** qPCR analyses of LPS-treated *Akt1s1*^*+/+*^ and *Akt1s1*^*-/-*^ BMDMs (*n* = 5). **C**, **D** qPCR analyses of mouse cLPMCs from control or DSS-treated mice (*n* = 5 for **C**; *n* = 3 for **D**). **E**, **F** Western blotting and qPCR analyses of THP-1 cells transfected with control or PRAS40 shRNA (**E**), or empty vector or PRAS40 expression vector (**F**). **G**–**I** THP-1 cells were introduced with empty vector or FLAG-PRAS40 expression vector, together with control or SLC7A5 shRNA. ELISA analyses of leucine and tryptophan levels (**G**), fluorescence analyses of FITC positive cells (**H**), western blotting and qPCR analyses (**I**). The quantification values of the band density normalized to α-Tubulin were labeled above the bands. Data represent the mean ± SD. *P < 0.05; **P < 0.01; ***P < 0.001.

Given that PRAS40 increased intracellular levels while decreased extracellular concentrations of leucine and tryptophan, and that supplementation with either amino acid failed to induce pro-inflammatory polarization in PRAS40-deleted macrophages, PRAS40 likely regulates their specific transporters. Analysis of the GSE38713 dataset for known transporters of leucine and tryptophan revealed significantly elevated expression of SLC6A14, SLC7A5, SLC7A6, SLC36A4 and SLC43A1 in UC samples (Fig. 5A) [33]. However, only SLC7A5 expression was downregulated in *Akt1s1*^-/-^ BMDMs (Fig. 5B, Supplementary Fig. 6A). The in vivo DSS challenge demonstrated robust induction of SLC7A5 expression in cLPMCs. However, this induction was attenuated in the cLPMCs from DSS-treated *Akt1s1*^*f/f*^*; Lyz2 Cre* mice compared to those from *Akt1s1*^*f/f*^ controls (Fig. 5C, D). Consistently, SLC7A5 expression exhibited significant upregulation in pro-inflammatory macrophages, which was suppressed by PRAS40 knockdown, and enhanced by PRAS40 overexpression (Fig. 5E, F, Supplementary Fig. 8P, Q).

Furthermore, SLC7A5 depletion reversed the increased uptake of leucine and tryptophan induced by PRAS40 overexpression (Fig. 5G, H). Meanwhile, the depletion or pharmacological inhibition of SLC7A5 by JPH203 reduced the expression of pro-inflammatory macrophage markers upregulated by PRAS40 overexpression (Fig. 5I, Suppl. Fig. 6B, Supplementary Fig. 8R). Conversely, SLC7A5 overexpression restored the expression of pro-inflammatory macrophage markers downregulated in PRAS40 depleted-macrophages (Suppl. Fig. 6C, Suppl. Fig. 8S). Taken together, PRAS40 upregulates SLC7A5 to enhance the uptake of leucine and tryptophan, thereby driving macrophage pro-inflammatory polarization.

### SP1 is the key transcription factor mediating PRAS40-induced SLC7A5 expression in macrophage pro-inflammatory polarization

**Fig. 6:**
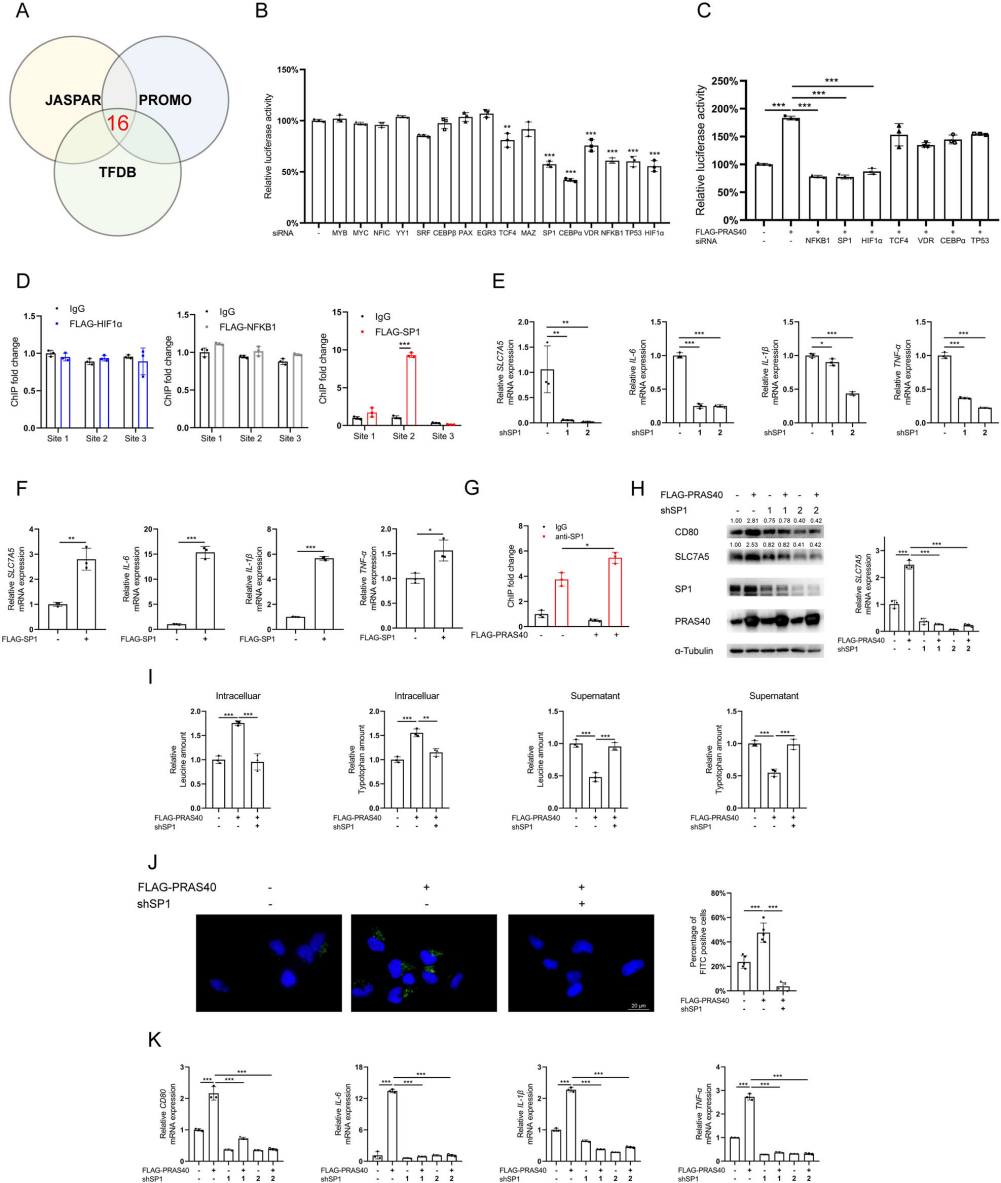
Improvement of PRAS40 on SLC7A5 transcription through SP1. **A** Transcription factor prediction for SLC7A5 using JASPER (https://jaspar.elixir.no/), PROMO (https://alggen.lsi.upc.es/cgi-bin/promo_v3/promo/promoinit.cgi?dirDB= TF_8.3) and TFDB (https://ngdc.cncb.ac.cn/databasecommons/database/id/8) databases. **B**, **C** luciferase assays in THP-1 cells introduced with siRNAs only (**B**) or together with empty vector or FLAG-SLC7A5 expression vector (**C**). **D** ChIP assays assessing the binding of HIF1α, NFKB1 or SP1 to SLC7A5 promoter in THP-1 cells. **E**, **F** qPCR analyses of THP-1 cells transfected with control or SP1 shRNA (**E**), or empty vector or SP1 expression vector (**F**). **G** ChIP assays assessing SP1 binding to SLC7A5 promoter in THP-1 cells introduced with empty vector or FLAG-PRAS40 expression vector. **H**–**K** THP-1 cells introduced with empty vector or FLAG-PRAS40 expression vector, together with control or SP1 shRNA. western blotting and qPCR analyses (**H**), ELISA analyses of leucine and tryptophan levels (**I**), fluorescence analyses of FITC positive cells (**J**) and qPCR analyses (**K**). The quantification values of the band density normalized to α-Tubulin were labeled above the bands. Data represent the mean ± SD. *P < 0.05; **P < 0.01; ***P < 0.001.

Given that both mRNA and protein levels of SLC7A5 were upregulated by PRAS40 in pro-inflammatory macrophages, we systematically explored the potential transcription factors governing SLC7A5 expression. Initial database analyses using JASPAR, PROMO and TFDB identified 16 candidates (Fig. 6A). Through luciferase reporter assays with an SLC7A5 promoter construct, we found that TCF4, SP1, CEBPα, VDR, NFKB1, TP53 and HIF1α silencing reduced the reporter activity (Fig. 6B). Further specificity analyses revealed that only depletion of NFKB1, SP1 and HIF1α counteracted the enhancement of reporter activity induced by PRAS40 overexpression in pro-inflammatory macrophages (Fig. 6C). Chromatin immunoprecipitation (ChIP) assays provided direct evidences of SP1 binding to the SLC7A5 promoter in pro-inflammatory macrophages, while NFKB1 and HIF1α showed no such interaction (Fig. 6D). Furthermore, SP1 depletion downregulated both SLC7A5 expression and pro-inflammatory macrophage markers, while SP1 overexpression showed the opposite effects (Fig. 6E, F). These findings collectively identify SP1 as the principal transcription factor responsible for SLC7A5 transcriptional activation in macrophage pro-inflammatory polarization.

We next investigated the role of SP1 in PRAS40-triggered SLC7A5 upregulation and macrophage pro-inflammatory polarization. PRAS40 overexpression substantially increased SP1 binding to the SLC7A5 promoter (Fig. 6G). SP1 knockdown eliminated PRAS40-induced upregulation of SLC7A5 expression (Fig. 6H, Supplementary Fig. 8T). Consistent with these molecular observations, SP1 depletion reversed PRAS40-induced increases in leucine and tryptophan uptake, as well as pro-inflammatory macrophage marker expression (Fig. 6I–K). SP1 overexpression restored PRAS40 deletion-downregulated expression of SLC7A5 and CD80 (Supplementary Fig. 7A, Supplementary Fig. 8U). Therefore, PRAS40 stimulates SLC7A5 transcription and promotes macrophage pro-inflammatory polarization through an SP1-dependent mechanism.

### PRAS40 maintains SP1 stability by promoting ERK- and JNK-induced SP1 phosphorylation

**Fig. 7:**
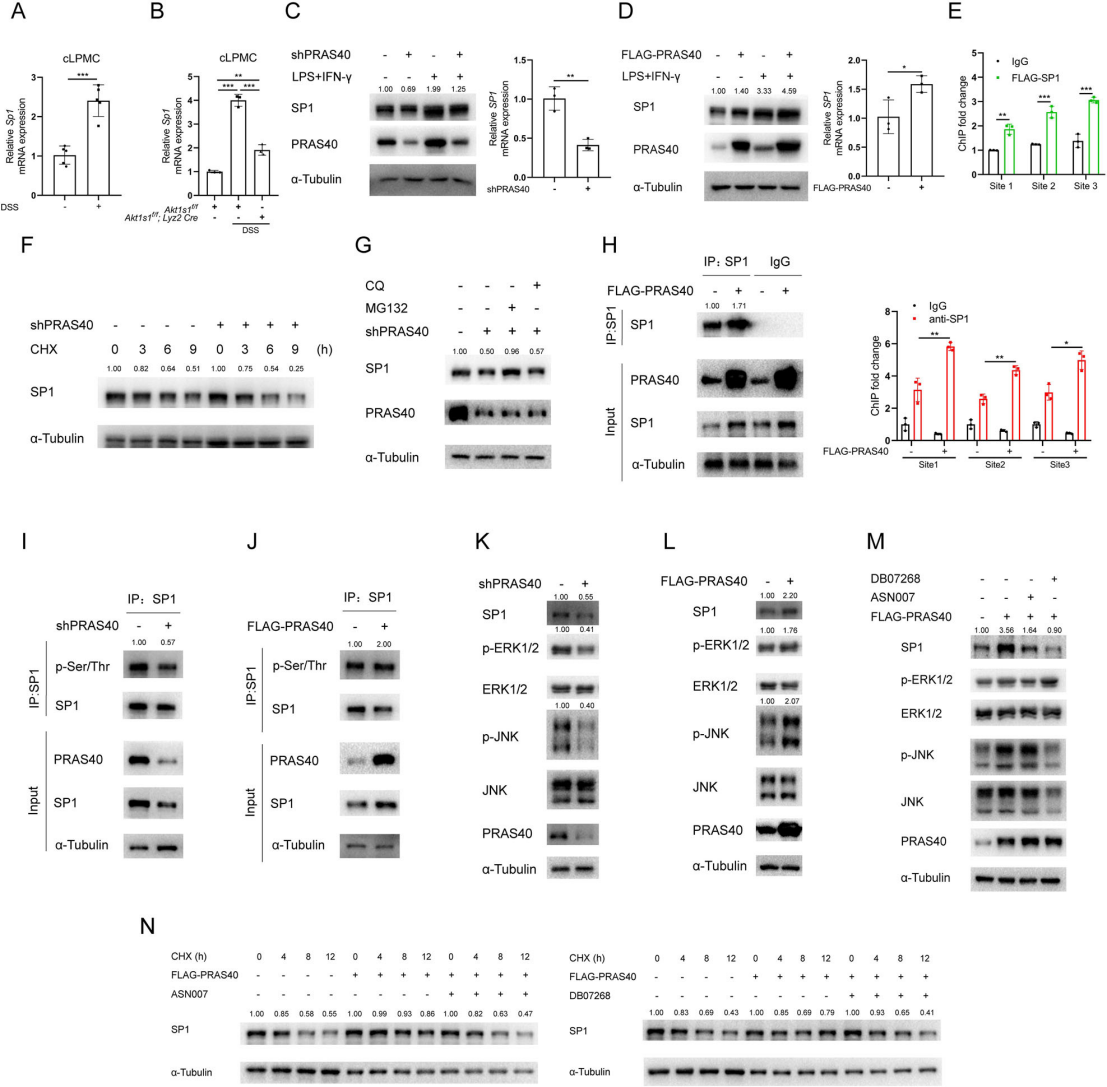
Regulation of SP1 stability by PRAS40. **A**, **B** qPCR analyses of mouse cLPMCs from control or DSS-treated mice (*n* = 5 for **A**; *n* = 3 for **B**). **C**, **D** Western blotting and qPCR analyses of THP-1 cells transfected with control or PRAS40 shRNA (**C**), or empty vector or PRAS40 expression vector (**D**). **E** ChIP assays assessing SP1 protein binding to SP1 promoter in THP-1 cells. **F**, **G** western blotting analyses of THP-1 cells introduced with control or PRAS40 shRNA, and treated with protein translation inhibitor CHX (100 μg/ml) (**F**), lysosome inhibitor chloroquine (CQ) (10 μM), or MG132 (5 μM) (**G**). **H** ChIP assays assessing SP1 protein binding to SP1 promoter in THP-1 cells. Western blotting analyses for precipitated SP1 protein and qPCR analyses for bound DNA were shown. **I**, **J** Co-IP followed by western blotting analyses in THP-1 cells transfected with control or PRAS40 shRNA (**I**), with empty vector or PRAS40 expression vector (**J**). **K**, **L** western blotting analyses of THP-1 cells transfected with control or PRAS40 shRNA (**K**), or empty vector or PRAS40 expression vector (**L**). **M**, **N** THP-1 cells transfected with empty vector or PRAS40 expression vector, and treated with ERK1/2 inhibitor ASN007 (50 nM) or JNK1/2 inhibitor DB07268 (50 μM). Western blotting analyses (**M**). Western blotting analyses after CHX treatment (**N**). The quantification values of the band density normalized to α-Tubulin were labeled above the bands. Data represent the mean ± SD. *P < 0.05; **P < 0.01; ***P < 0.001.

To elucidate the mechanism by which PRAS40 regulates SP1, we first measured SP1 expression levels. In vivo analyses revealed that DSS treatment significantly induced SP1 expression in mouse cLPMCs. However, this induction was markedly attenuated in *Akt1s1*^*f/f*^*; Lyz2 Cre* mice compared to *Akt1s1*^*f/f*^ mice (Fig. 7A, B). Meanwhile, in THP-1 cells, PRAS40 knockdown downregulated both SP1 mRNA and protein levels, while PRAS40 overexpression upregulated them (Fig. 7C, D, Supplementary Fig. 8V–W). Since SP1 has been reported to autoregulate its own transcription in cancer cells and in vitro [34, 35], we tested whether this autoregulatory mechanism operates in pro-inflammatory macrophages. SP1 protein binding to its own promoter in pro-inflammatory macrophages was confirmed by ChIP (Fig. 7E). Additionally, PRAS40 knockdown did not alter the mRNA stability of SP1 (Supplementary Fig. 7B). Cycloheximide (CHX) chase assays revealed that the stability of SP1 protein was significantly reduced by PRAS40 elimination (Fig. 7F, Supplementary Fig. 8X). Further, the proteasome inhibitor MG132, but not the lysosome inhibitor chloroquine (CQ), significantly restored SP1 protein levels in the PRAS40-depleted macrophages (Fig. 7G, Supplementary Fig. 8Y). Therefore, PRAS40 stabilizes SP1 by protecting it from proteasomal degradation in pro-inflammatory macrophages. Given that the PRAS40-mediated enhancement of SP1 binding to its promoter was quantitatively comparable to its upregulation on SP1 protein level, we conclude that PRAS40-trigged alterations in SP1 mRNA levels are secondary to protein-level regulation (Fig. 7H, Supplementary Fig. 8Z).

The stability of SP1 are known to be controlled by phosphorylation [36]. We therefore examined the effects of PRAS40 on SP1 phosphorylation. SP1 serine/threonine phosphorylation was reduced upon PRAS40 knockdown while increased upon PRAS40 overexpression (Fig. 7I, J, Supplementary Fig. 8AA, AB). Since ERK and JNK are key regulators of SP1 phosphorylation and stability [36], we next assessed the impact of PRAS40 on their activation. PRAS40 depletion significantly inhibited the phosphorylation of ERK and JNK, whereas PRAS40 overexpression enhanced it (Fig. 7K, L, Supplementary Fig. 8AC–AD). Importantly, the ERK inhibitor ASN007 and the JNK inhibitor DB07268 reversed the PRAS40-induced increase in SP1 expression and stability (Fig. 7M, N, Supplementary Fig. 8AE–AF). In conclusion, PRAS40 promotes SP1 phosphorylation by activating ERK and JNK signaling pathways, thereby increasing SP1 stability in pro-inflammatory macrophages.

## Discussion

The pathogenesis of IBD remains incompletely understood, due to its complex interplay of genetic, environmental, microbial, and immune factors. This incomplete mechanistic understanding likely contributes to the limitations of current therapies, which often fail to induce or sustain long-term clinical remission. Therefore, gaining deeper insights into the pathogenic drivers of IBD is essential for developing more effective treatment strategies [1, 7]. In this study, we demonstrate that PRAS40 is hyperexpressed in IBD clinical samples and mouse tissues, and exacerbates IBD by promoting pro-inflammatory macrophage polarization, as validated in both PRAS40 global and myeloid-specific knockout mice. Mechanistically, PRAS40 stabilizes the transcription factor SP1 through enhancing its phosphorylation, thereby promoting amino acid transporter SLC7A5 expression. The subsequent increase in intracellular leucine and tryptophan levels activates mTOR signaling, ultimately mediating PRAS40-induced pro-inflammatory polarization of macrophages. These findings highlight the therapeutic potential of targeting the PRAS40/SP1/SLC7A5 axis for IBD treatment.

PRAS40, an Akt1 substrate and mTORC1 component, has been studied in the context of cancer, where it regulates cancer cell survival, proliferation, stemness, autophagy and metastasis [22–24, 26, 27]. Although PRAS40 expression is upregulated in colon cancer cells, we demonstrate here that PRAS40 expression in IECs is not altered in IBD, nor does intestinal epithelial PRAS40 affect IBD development. Furthermore, PRAS40 expression is upregulated in pro-inflammatory macrophages and it functionally promotes IBD progression through enhancing macrophage pro-inflammatory polarization. Our findings thus reveal a novel function and mechanism of PRAS40 in regulating macrophage pro-inflammatory polarization, providing new insights into its role in IBD pathogenesis. Targeting PRAS40 in macrophages may offer a promising therapeutic strategy for IBD, a possibility we are actively investigating. Considering the complex cellular microenvironment of colon, where multiple cell types contribute to IBD pathogenesis, we acknowledge the potential involvement of PRAS40 in modulating other cellular functions during disease progression. This aspect will be systematically explored in our future studies.

Macrophages are crucial in maintaining intestinal homeostasis, while their aberrant activation is a key driver of IBD progression [3, 7]. The transition from a homeostatic to a pro-inflammatory state is tightly regulated, with metabolic reprogramming playing a critical role. Among metabolic inputs, leucine is known to stimulate mTOR signaling to promote pro-inflammatory polarization, whereas the role of tryptophan and its metabolites appears more complex, with both pro- and anti-inflammatory effects reported [14, 15, 17]. Our study establishes that PRAS40 drives macrophage pro-inflammatory polarization by specifically facilitating the uptake of both leucine and tryptophan, leading to mTOR activation. Although PRAS40 deficiency broadly reduced the levels of multiple essential amino acids, only leucine and tryptophan were functionally critical for polarization, as supplementing others had no effect. Crucially, neither amino acid could adequately activate mTOR or induce polarization in the absence of PRAS40, establishing PRAS40 as an essential upstream regulator. This finding adds a new dimension to the previous understanding of PRAS40, which is viewed as an mTOR inhibitor, and aligns with our prior observations in cancer cells [22, 23, 37]. Our study thus clarifies a novel mechanism whereby PRAS40 positively regulates mTOR signaling in macrophages by controlling intracellular availability of key amino acids. Whether this regulatory axis operates in other physiological or pathological contexts warrants further investigation.

We further identified SLC7A5 as the key transporter responsible for the PRAS40-enhanced uptake of leucine and tryptophan. While SLC7A5 has been implicated in macrophage pro-inflammatory polarization in rheumatoid arthritis (RA) and metabolic dysfunction-associated steatohepatitis (MASH), primarily via leucine transport [36, 37], our work extends its functional scope that tryptophan constitutes another critical substrate for SLC7A5 in driving macrophage polarization. SLC7A5 regulation has been studied in cancer and epithelial cells, involving transcription factors such as NF-κB, HIF1α, c-MYC, JunD, TP63, ATF4, and FOXC1 [38–43]. Our data revealed that NFKB1 and HIF1α modulated PRAS40-induced SLC7A5 reporter expression but did not bind SLC7A5 promoter, indicating an indirect regulating mechanism. In contrast, we identify SP1 as a novel and critical transcription factor for SLC7A5 in macrophages.

SP1 regulates gene expression by binding to GC-rich promoter regions, and is involved in diverse cellular processes [36]. In macrophages, SP1 has been reported to facilitate macrophage protumor activation and anti-inflammatory polarization [44–46]. Surprisingly, our study reveals a novel role of SP1 in driving pro-inflammatory macrophage polarization, suggesting that SP1 may direct macrophages toward distinct functional states depending on the pathological context. Moreover, SP1 stability is markedly influenced by posttranslational modifications. Previous studies have shown that ERK-mediated phosphorylation at Thr278 and JNK-induced phosphorylation at Thr278/739 enhance SP1 protein stability [36]. In line with this, we found that PRAS40 activates ERK and JNK signaling, which in turn increases SP1 Ser/Thr phosphorylation and stabilize it. Inhibition of ERK and JNK signaling abolishes these effects, confirming the central role of PRAS40 in this regulatory cascade. These results parallel our previous report that PRAS40 positively regulates insulin receptor phosphorylation in tumor cells, leading to the downstream pathway activation. Whether PRAS40 engages in crosstalk with growth factors during macrophage pro-inflammatory polarization warrants future investigation.

In summary, our study elucidates a novel PRAS40/SP1/SLC7A5 axis that drives macrophage pro-inflammatory polarization by enhancing leucine and tryptophan uptake to activate mTOR signaling. This pathway contributes to IBD progression, highlighting its potential as a therapeutic target. By targeting PRAS40 or its downstream effectors, it may be possible to disrupt this pathogenic cascade and develop more effective treatments for IBD.

## Supplementary information

Supplementary materials

uncropped WB

## Data Availability

All the datasets analyzed during the current study are available on reasonable request.
