# Supplementary material for: PRAS40-driven pro-inflammatory macrophage polarization is required for inflammatory bowel disease
Source: Cell Death Dis. 2026 Jun 8;17(1):719. doi: 10.1038/s41419-026-08931-7 (PMC13469118; doi:10.1038/s41419-026-08931-7)
Supplement: Supplementary file 1 [file 41419_2026_8931_MOESM1_ESM.pdf]

PRAS40-driven proinflammatory macrophage polarization is required for inflammatory bowel disease

Chengfei Zhang[1*], Yufei Bo[1*], Jia Deng[1*], Ting Zhang[1, 2], Xinran Chen[1], Hongming Teng[1, 2], Yuanyuan Luo[1, 2], Lin Huang[1, 2]

[1]Department of Pathophysiology, College of Basic Medical Sciences, Dalian Medical University, 9 South Lvshun Road, Dalian, Liaoning 116044, P. R. China; [2]Liaoning Provincial Key Laboratory of Medical Cellular and Molecular Biology, 9 South Lvshun Road, Dalian, Liaoning 116044, P. R. China

Supplementary Tables

Supplementary Table 1 List of genotyping primers

Name	Sequence
MSD-F	AACTGGATGCATGAGAATCGGGACT
MSD-R	TCAGTAATACCAGCTCTGTGCCAGT
MSD-R1	GGGGAACCGGGATACAATTGTCAGG
Cre-F	AGTGCTGAAGTCCATAGATCGG
Cre-R	CTGATTCTCCTCATCACCAGG
Flox-F	GAGGAGCCCCACTGTAGTTTAGGAG
Flox-R	TCCGCATCCCACCTCCTTCTTCAAC

Supplementary Table 2 List of human primers

Name	Sequence
β-actin-F	TGGCACCCAGCACAATGAA
β-actin-R	CTAAGTCATAGTCCGCCTAGAAGCA
CD80-F	AAACTCGCATCTACTGGCAAA
CD80-R	GGTTCTTGTACTCGGGCCATA
IL-6-F	AGACAGCCACTCACCTCTTCAG
IL-6-R	TTCTGCCAGTGCCTCTTTGCTG

IL-1β-F	CCACAGACCTTCCAGGAGAATG
IL-1β-R	GTGCAGTTCAGTGATCGTACAGG
TNF-α-F	CTCTTCTGCCTGCTGCACTTTG
TNF-α-R	ATGGGCTACAGGCTTGTCACTC
SLC7A5-F	CCGTGAACTGCTACAGCGT
SLC7A5-R	CTTCCCGATCTGGACGAAGC
SP1-F	AGTTCCAGACCGTTGATGGG
SP1-R	GTTTGCACCTGGTATGATCTGT

Supplementary Table 3 List of mouse primers

Name	Sequence
β-actin-F	CATTGCTGACAGGATGCAGAAGG
β-actin-R	TGCTGGAAGGTGGACAGTGAGG
CD80-F	TGCTGCTGATTCGTCTTTCAC
CD80-R	GAGGAGAGTTGTAACGGCAAG
CD86-F	GAGCTGGTAGTATTTTGGCAGG
CD86-R	GGCCCAGGTACTTGGCATT
Il-6-F	TACCACTTCACAAGTCGGAGGC
Il-6-R	CTGCAAGTGCATCATCGTTGTTC
Il-1β-F	TGGACCTTCCAGGATGAGGACA
Il-1β-R	GTTCATCTCGGAGCCTGTAGTG
Tnf-α-F	GGTGCCTATGTCTCAGCCTCTT
Tnf-α-R	GCCATAGAACTGATGAGAGGGAG
Inos-F	GCAAACATCACATTCAGATCCC
Inos-R	TCAGCCTCATGGTAAACACG
CD206-F	CTCTGTTCAGCTATTGGACGC
CD206-R	TGGCACTCCCAAACATAATTTGA
Arg-1-F	CATTGGCTTGCGAGACGTAGAC

Arg-1-R	GCTGAAGGTCTCTTCCATCACC
Il-10-F	GCTCTTACTGACTGGCATGAG
Il-10-R	CGCAGCTCTAGGAGCATGTG
Slc7a5-F	CTACGCCTACATGCTGGAGG
Slc7a5-R	GAGGGCCGAATGATGAGCAG
Slc38a9-F	GGAACATGAAGTAAGCCCTGG
Slc38a9-R	TGGTCACTAACCCTCTGAATGA
Slc7a8-F	TGTGACTGAGGAACTTGTGGA
Slc7a8-R	GTGGACAGGGCAACAGAAATG
Slc38a7-F	CACCACTTCGACGCTTGGA
Slc38a7-R	GTAGGCCAGAATGACCAGCC
Slc36a4-F	TATTGGTACGTTGCAGTCACTTT
Slc36a4-R	CATGGCAAAACTCACGGTGT
Zo-1-F	GCCGCTAAGAGCACAGCAA
Zo-1-R	GCCCTCCTTTTAACACATCAGA
Ocln-F	TACTGGTCTCTACGTGGATCAAT
Ocln-R	TTCTTCGGGTTTTCACAGCAA
Cldn1-F	TGCCCCAGTGGAAGATTTACT
Cldn1-R	CTTTGCGAAACGCAGGACAT

Supplementary Table 4 List of ChIP primers

Name	Sequence
SP1-SLC7A5-1-F	AAGCCAGCAATCTGCCTGAG
SP1-SLC7A5-1-R	CTTCACACTGAAGCCTGCCT
SP1-SLC7A5-2-F	CTGTCGTGCTGGGACCTCTA
SP1-SLC7A5-2-R	CTCGGTTTTATCACCGCAGA
SP1-SLC7A5-3-F	GCACGTGCGTCGTCCG
SP1-SLC7A5-3-R	CATGGCGCAGGAGGACTG

H1F1A-SLC7A5-1-F	GCACGTGCGTCGTCC
H1F1A-SLC7A5-1-R	CATGGCGCAGGAGGACT
H1F1A-SLC7A5-2-F	GTTCCTTGGCGTGTGTGC
H1F1A-SLC7A5-2-R	TTGCTGTGAACACCCAGCTC
H1F1A-SLC7A5-3-F	AACCGGGGGTTCGCTGG
H1F1A-SLC7A5-3-R	GAAGCCTGCCTTTGAATTCCCC
NFKB1-SLC7A5-1-F	CTAGGCTCCTGTCCCGGAT
NFKB1-SLC7A5-1-R	CCCACTGATGCAGCCCC
NFKB1-SLC7A5-2-F	CAGCCTGGCGGGCTATTC
NFKB1-SLC7A5-2-R	GAGGACGGGCTGGGATGA
NFKB1-SLC7A5-3-F	ACCGGGGGTTCGCTGG
NFKB1-SLC7A5-3-R	TTTTAGCTAAGATGCCTCTCGCA
SP1-SP1-1-F	TGGCAGGATTTGAGCTCACTG
SP1-SP1-1-R	CAGACCTGTAATCCCAGCAC
SP1-SP1-2-F	TGCTGTCACCAGATCTCGTC
SP1-SP1-2-R	ATTAGGTGGGACGCTTTAGC
SP1-SP1-3-F	CCTGAGGAGGGCCAATATGG
SP1-SP1-3-R	CTCAGTTATTCAATCCAATGAG

Supplementary Figures

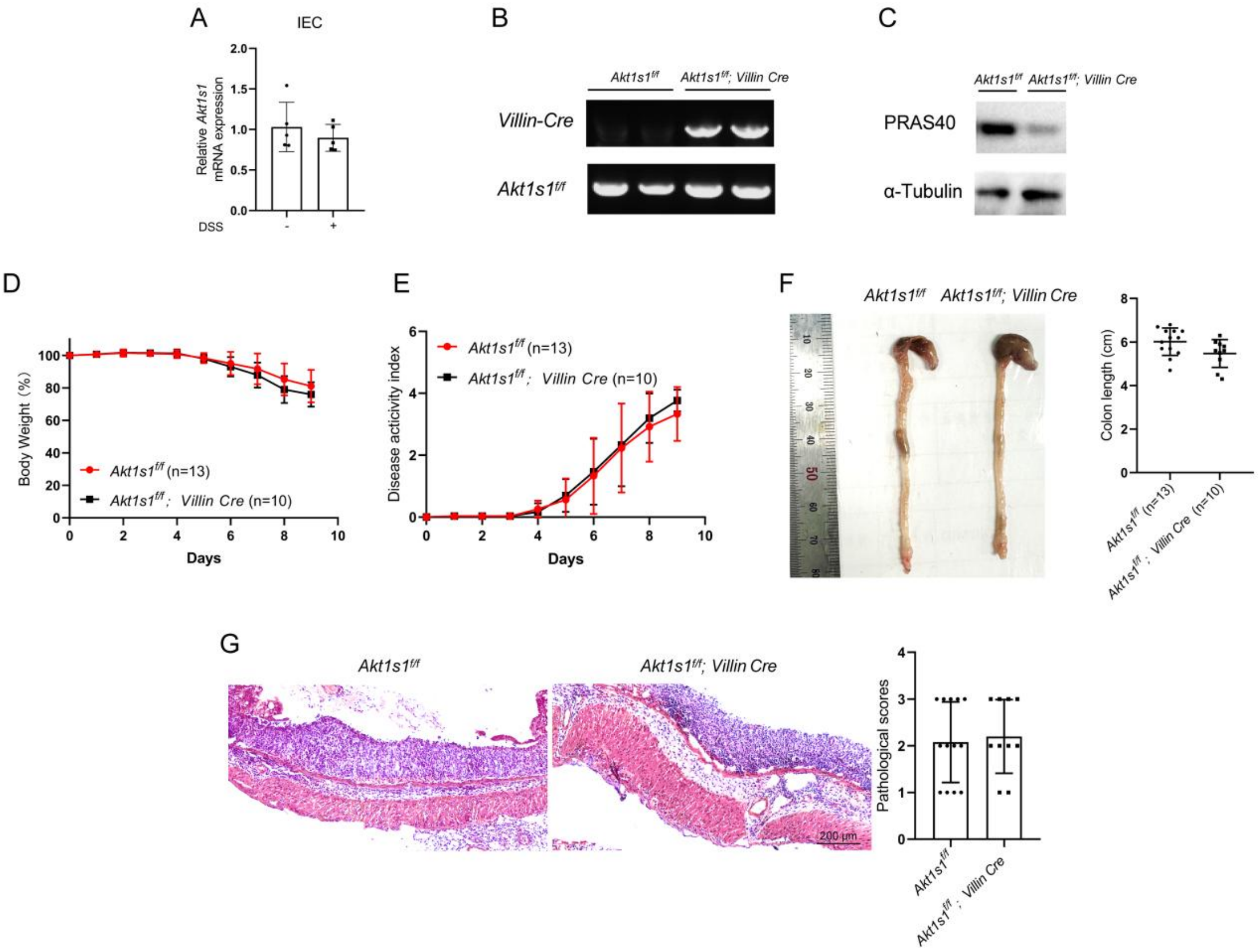

Supplementary Fig. 1. Effects of epithelial PRAS40 on DSS-treated mice.

A, qPCR analyses of IECs from control (n=5) or DSS-treated mice (n=5). B-G, DSS-induced colitis in *Akt1s1*$^{f/f}$ (n=13) and *Akt1s1*$^{f/f}$*; Villin Cre* mice (n=10). Genotyping results (B), western blotting analyses of mouse colon tissues (C), body weights (D), disease activity index (DAI)(E), colon lengths (F) and H&E staining of colon tissues and corresponding pathological scores (G).

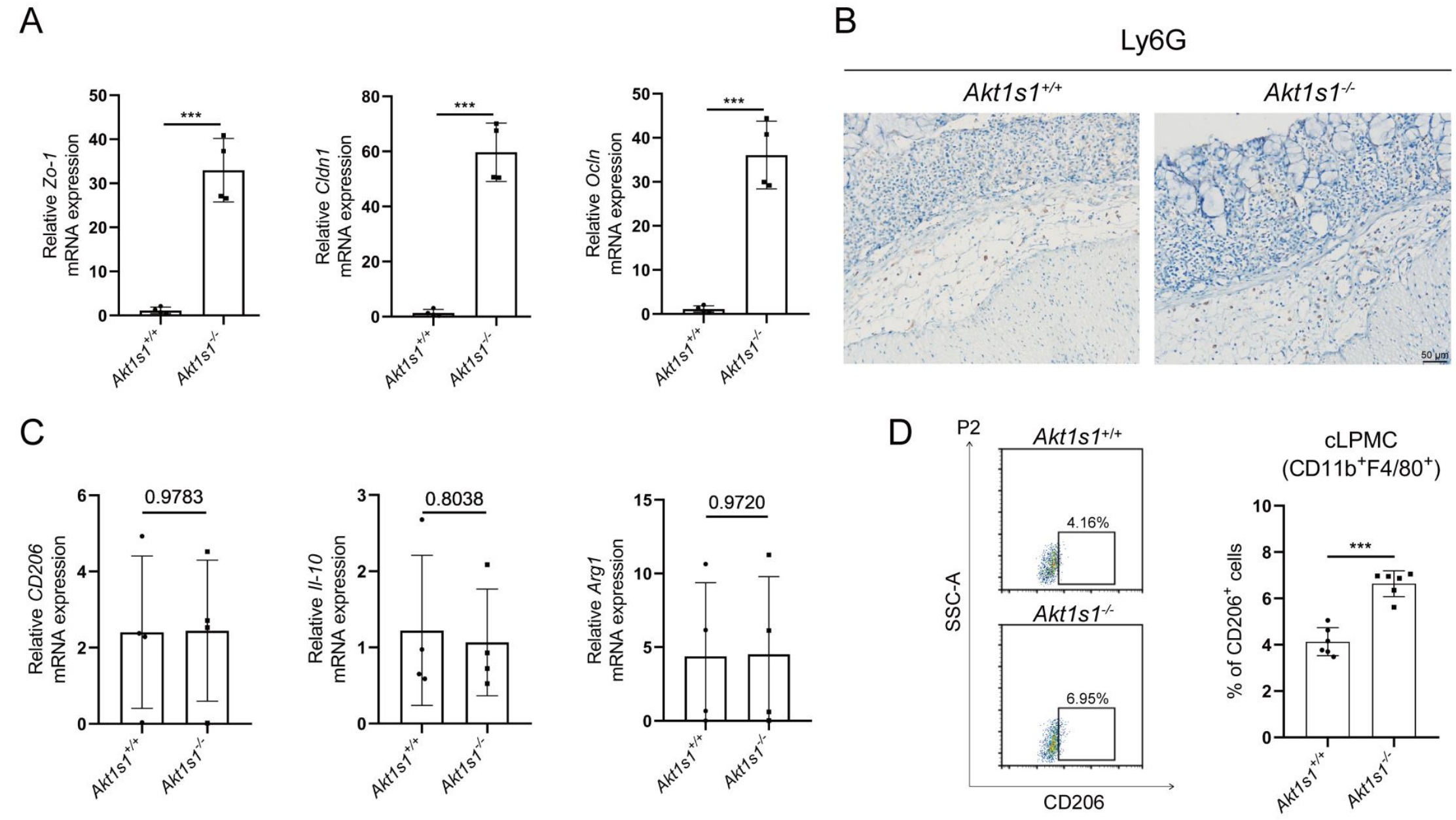

Supplementary Fig. 2. Analyses in PRAS40 global knockout mice.

A, qPCR analyses of mouse colon tissues (n=4). B, IHC staining with anti-Ly6G antibody. C, qPCR analyses of mouse colon tissues (n=4). D, flow cytometry analyses of mouse cLPMCs (n=6) with anti-F4/80, anti-CD11b and anti-CD206 antibodies. Data represent the mean ± SD. ****P* < 0.001.

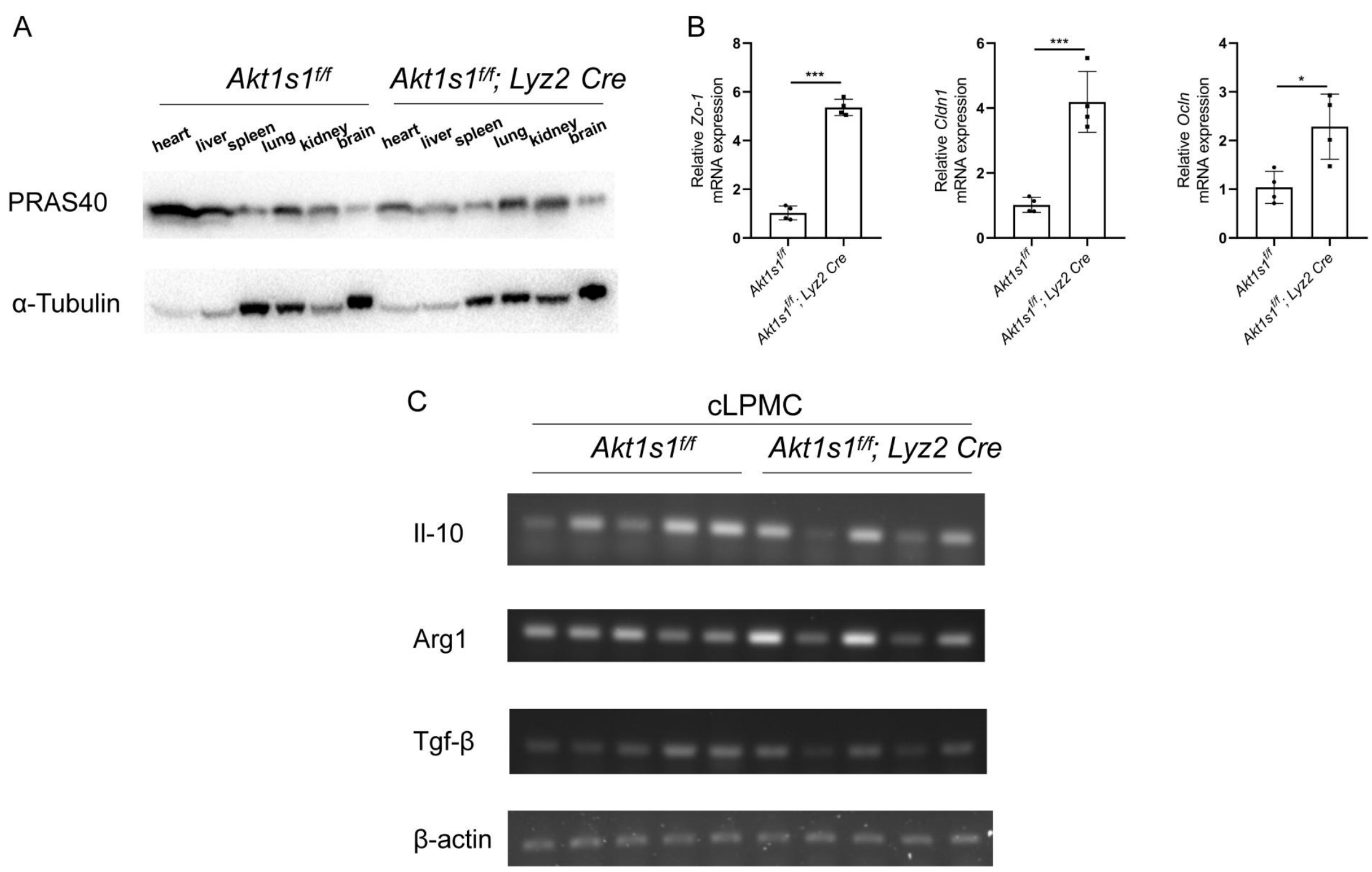

Supplementary Fig. 3. Analyses in PRAS40 myeloid-specific knockout mice.

A, western blotting analyses of different tissues. B, qPCR analyses of mouse colon tissues (n=4). C, PCR analyses of mouse cLPMCs. Data represent the mean ± SD. *$P < 0.05$; ***$P < 0.001$.

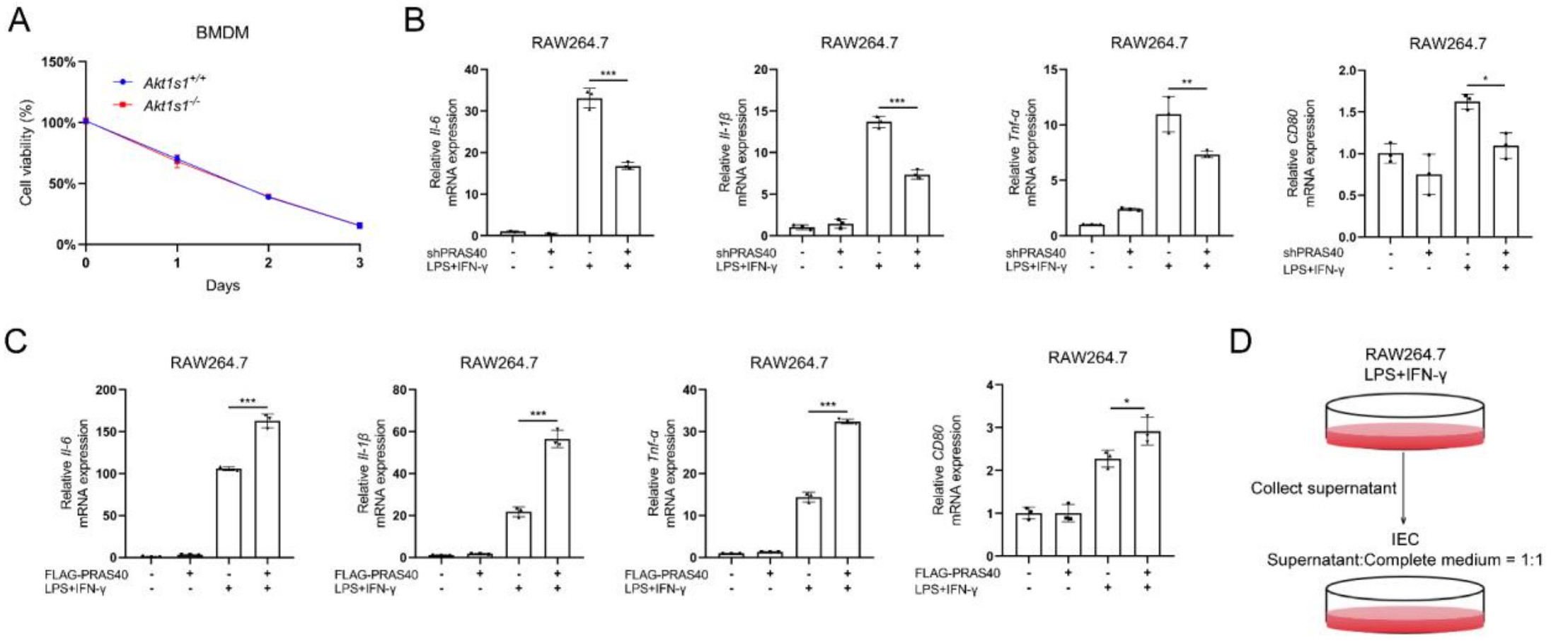

Supplementary Fig. 4. Effects of PRAS40 on macrophages in *vitro*.

A, cell viability assays in *Akt1s1*$^{+/+}$ and *Akt1s1*$^{-/-}$ BMDMs. B-C, qPCR analyses of RAW264.7 cells transfected with control or PRAS40 shRNA (B), or empty vector or PRAS40 expression vector (C). D, schematic description of CM treatment in mouse IECs. Data represent the mean ± SD. *$P < 0.05$; **$P < 0.01$; ***$P < 0.001$.

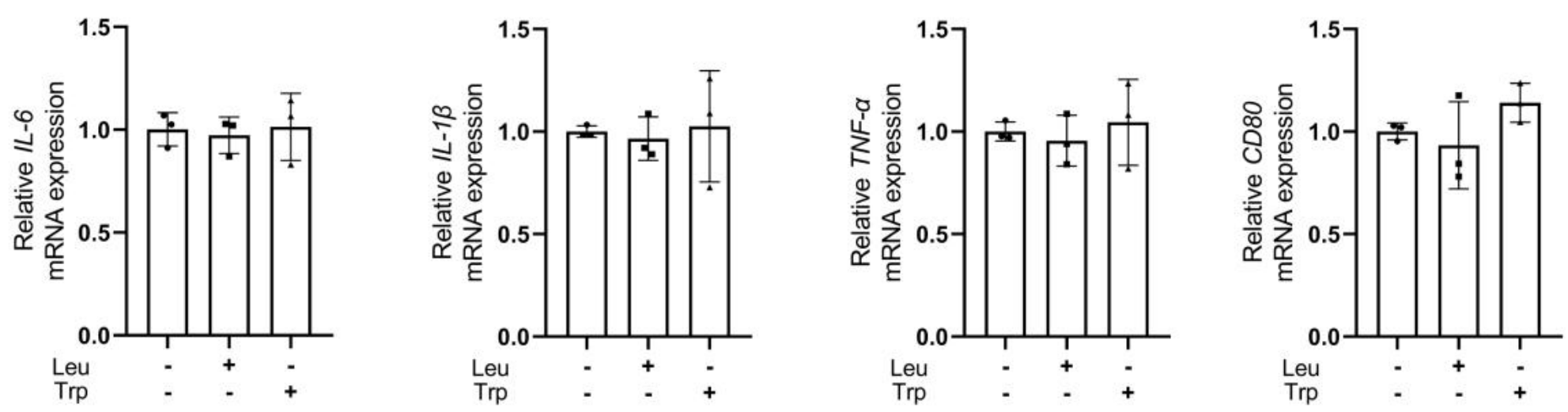

Supplementary Fig. 5. Effects of leucine and tryptophan on the expression of cytokines in macrophages.

qPCR analyses of THP-1 cells treated with Leu (2 mM) or Trp (100 μM).

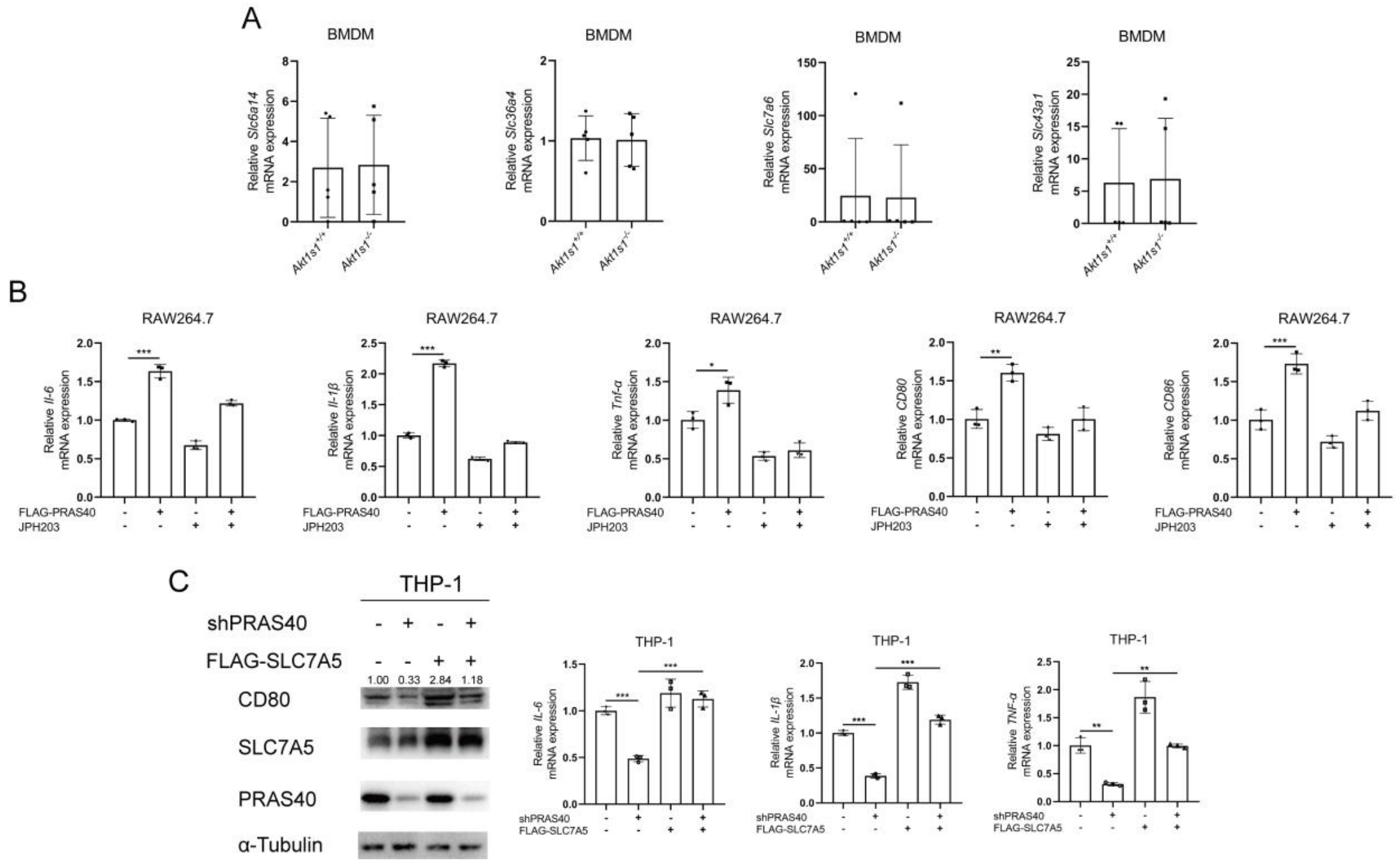

Supplementary Fig. 6. Effects of PRAS40 on the uptake of leucine and tryptophan.

A-B, qPCR analyses of LPS-treated *Akt1s1*$^{+/+}$ and *Akt1s1*$^{-/-}$ BMDMs (n=5) (A), and RAW264.7 cells introduced with empty vector or FLAG-PRAS40 expression vector and treated with SLC7A5 inhibitor JPH203 (10 μM or 50 μM) (B). C, western blotting and qPCR analyses of THP-1 cells introduced with control or PRAS40 shRNA, together with empty vector or FLAG-SLC7A5 expression vector. The quantification values of the band density normalized to α-Tubulin were labeled above the bands. Data represent the mean ± SD. *$P < 0.05$; **$P < 0.01$; ***$P < 0.001$.

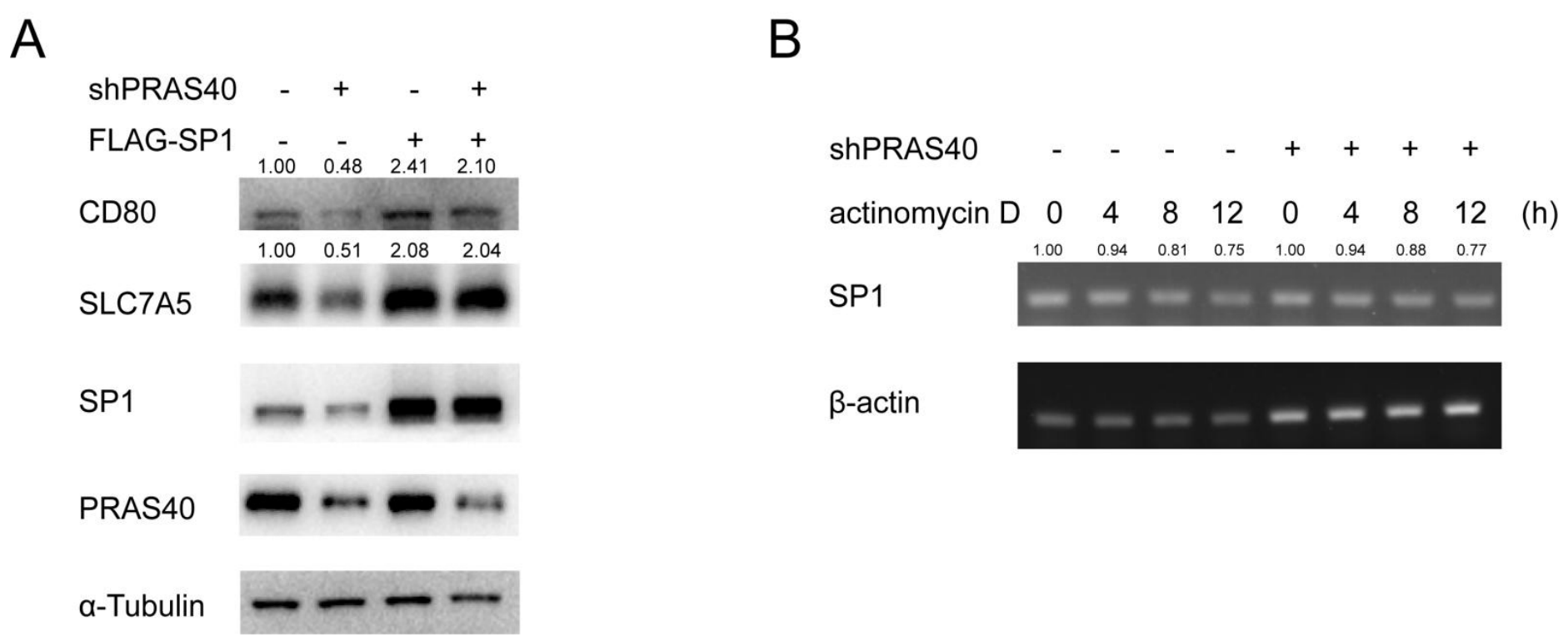

Supplementary Fig. 7. Effects of PRAS40 on SLC7A5 and SP1.

A, western blotting analyses of THP-1 cells introduced with control or PRAS40 shRNA together with empty vector or FLAG-SP1 expression vector. B, PCR analyses of THP-1 cells introduced with control or PRAS40 shRNA and treated with

transcription inhibitor actinomycin D (5 μg/ml). The quantification values of the band density normalized to α-Tubulin or β-actin were labeled above the bands.
