# Supplementary material for: PRAS40-driven pro-inflammatory macrophage polarization is required for inflammatory bowel disease
Source: Cell Death Dis. 2026 Jun 8;17(1):719. doi: 10.1038/s41419-026-08931-7 (PMC13469118; doi:10.1038/s41419-026-08931-7)

Supplementary Fig. 8A

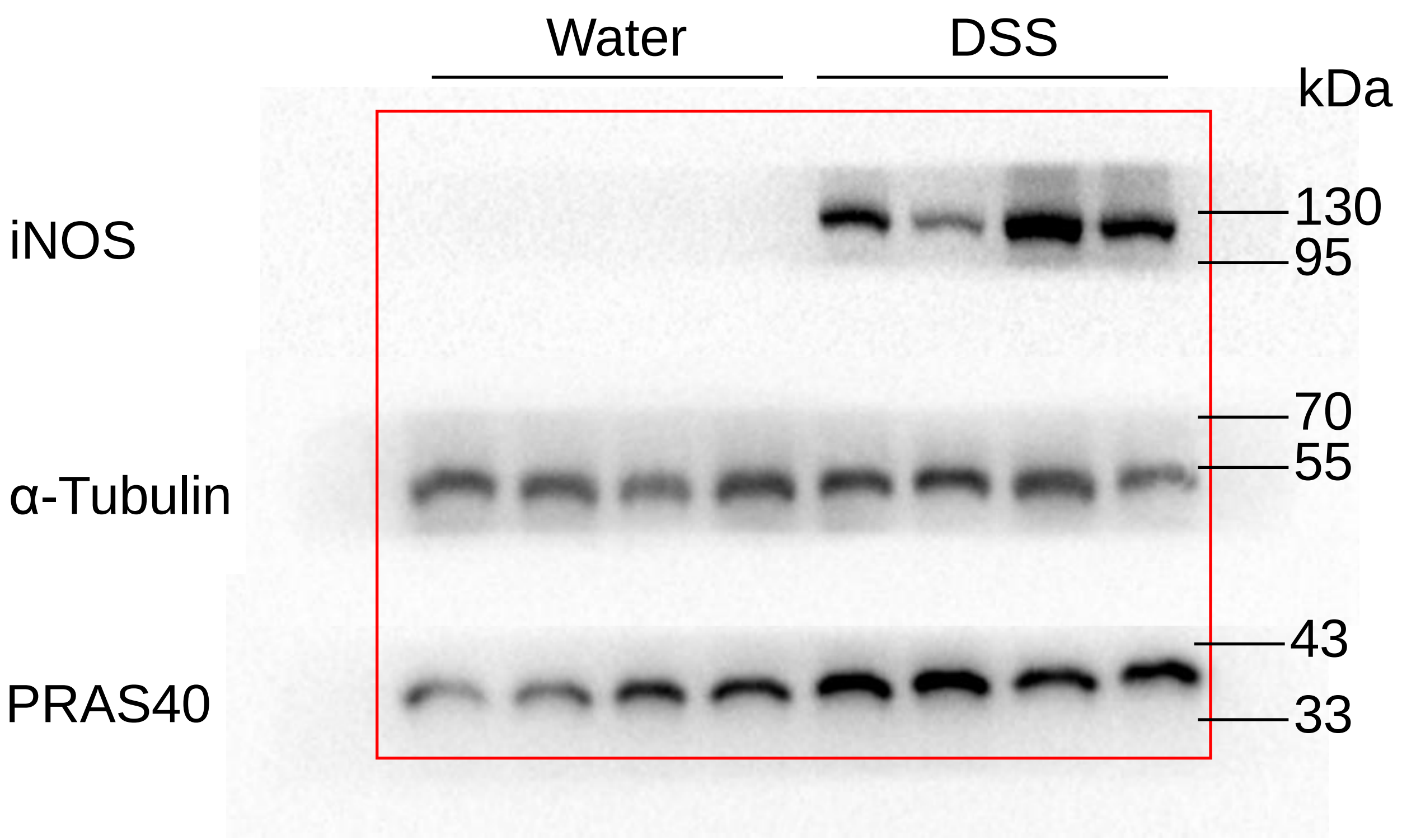

Correlated to Fig. 1B

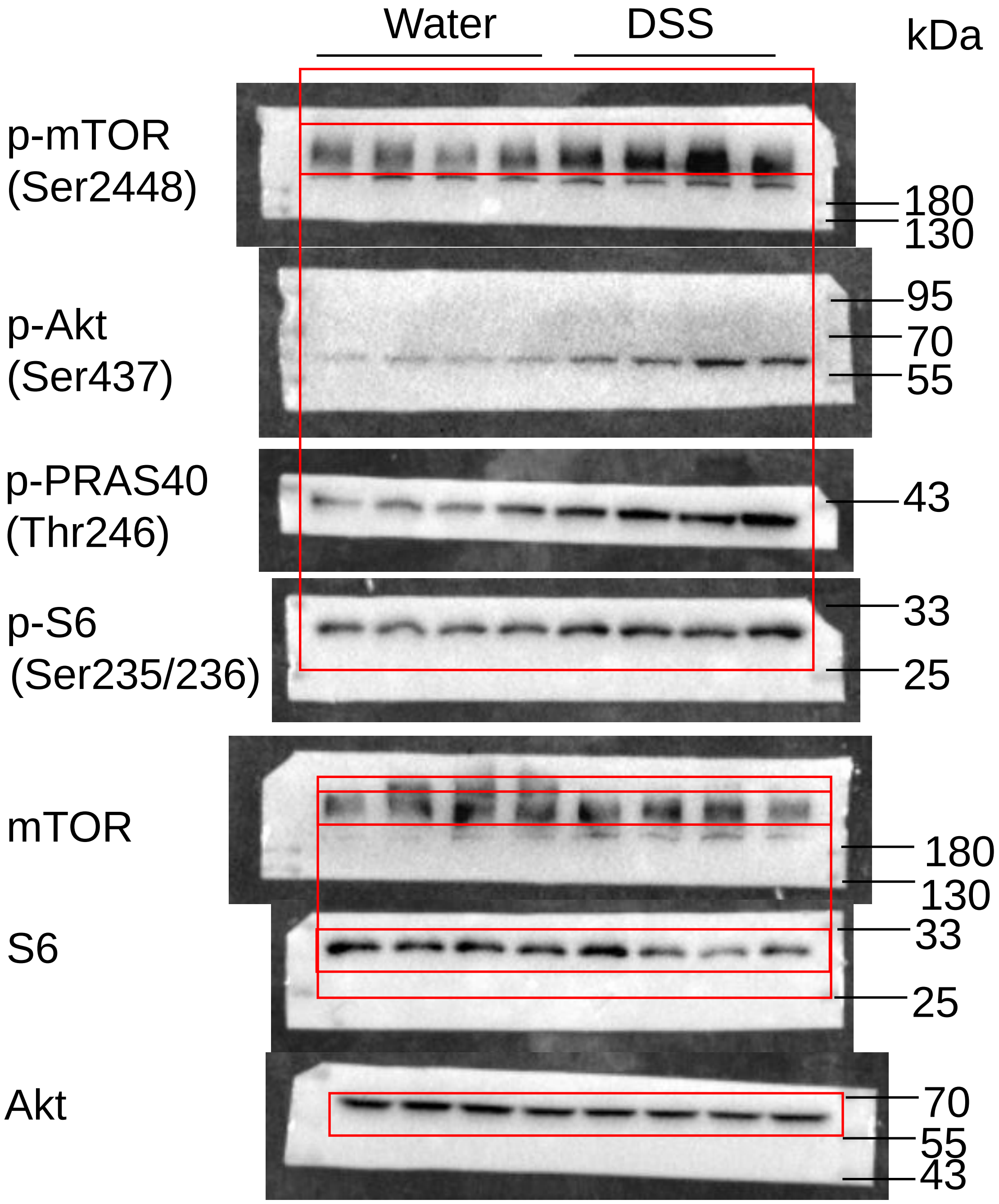

Supplementary Fig. 8B

Correlated to Fig. 1K

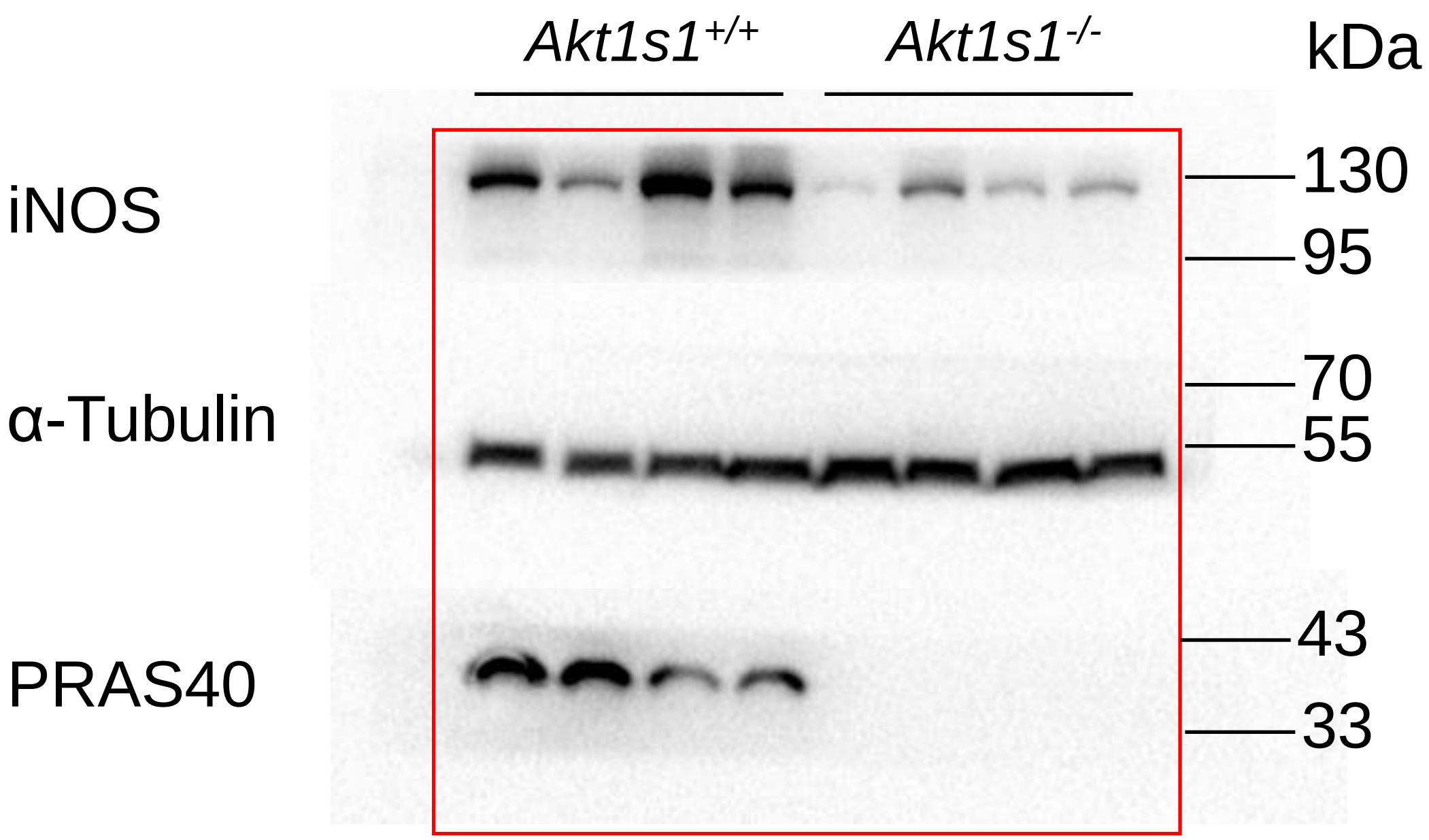

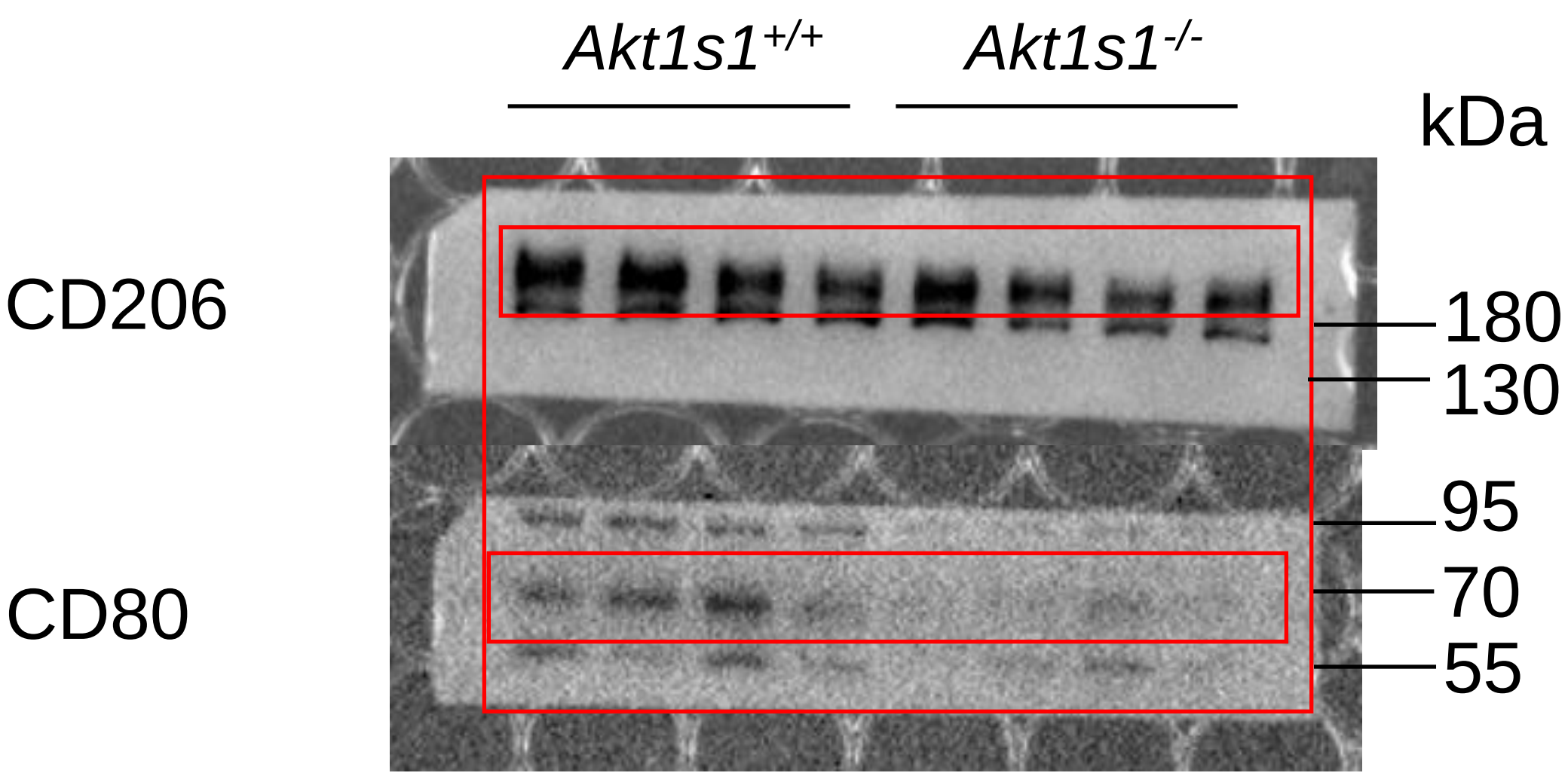

Supplementary Fig. 8C

Correlated to Fig. 1L

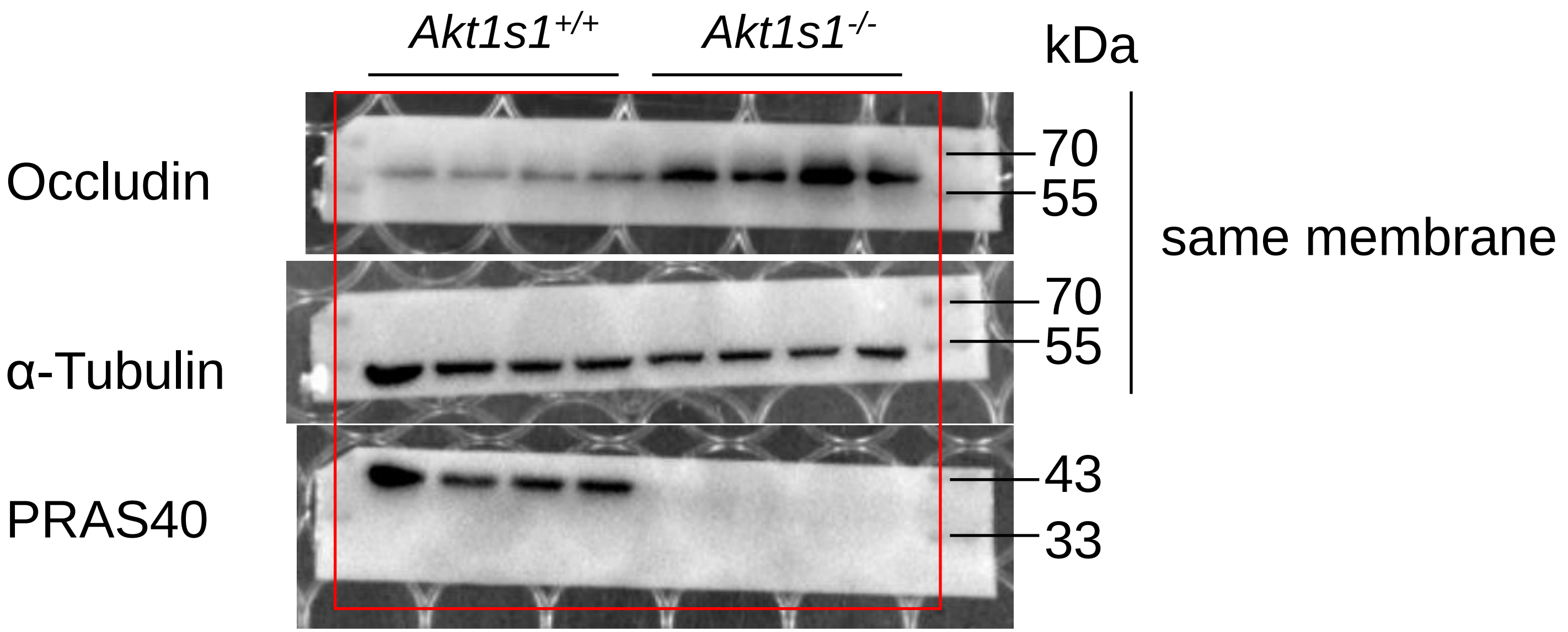

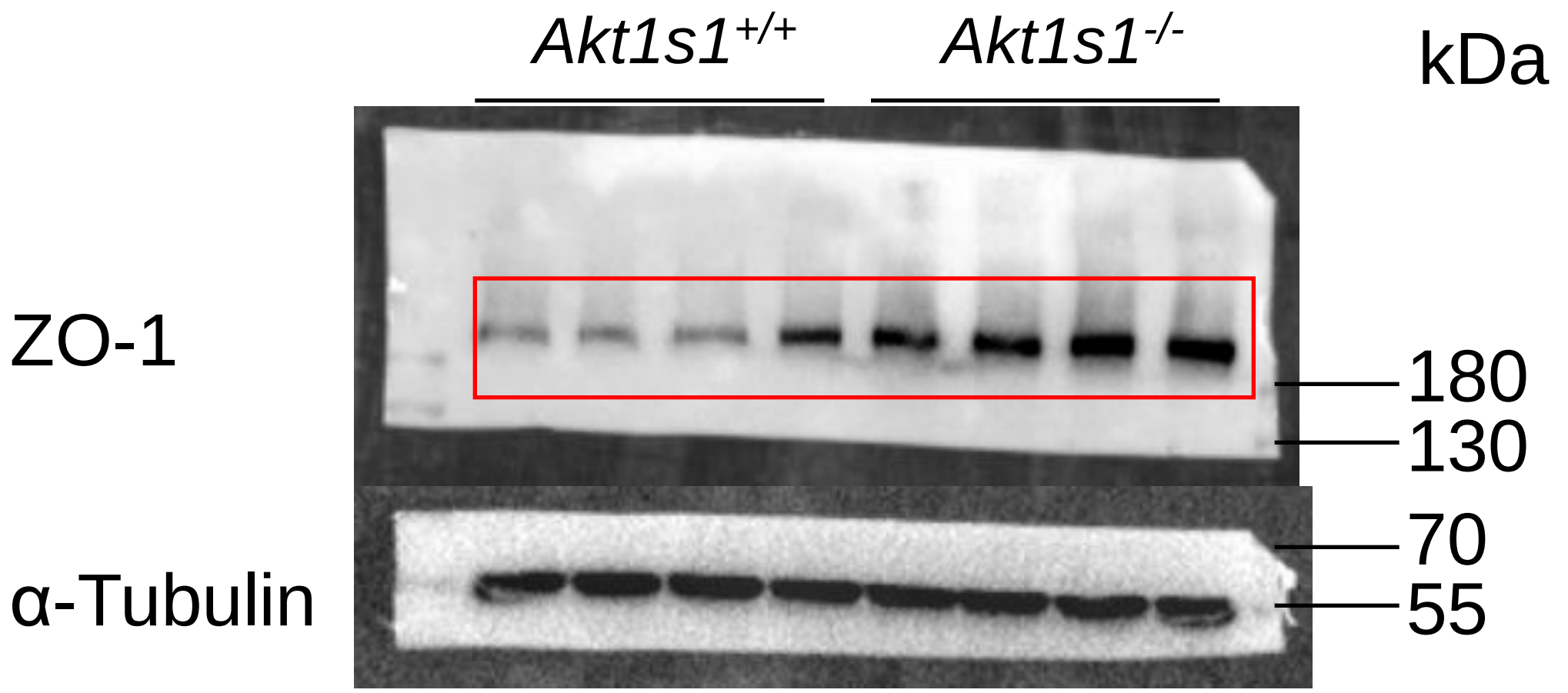

Supplementary Fig. 8D

Correlated to Fig. S1C

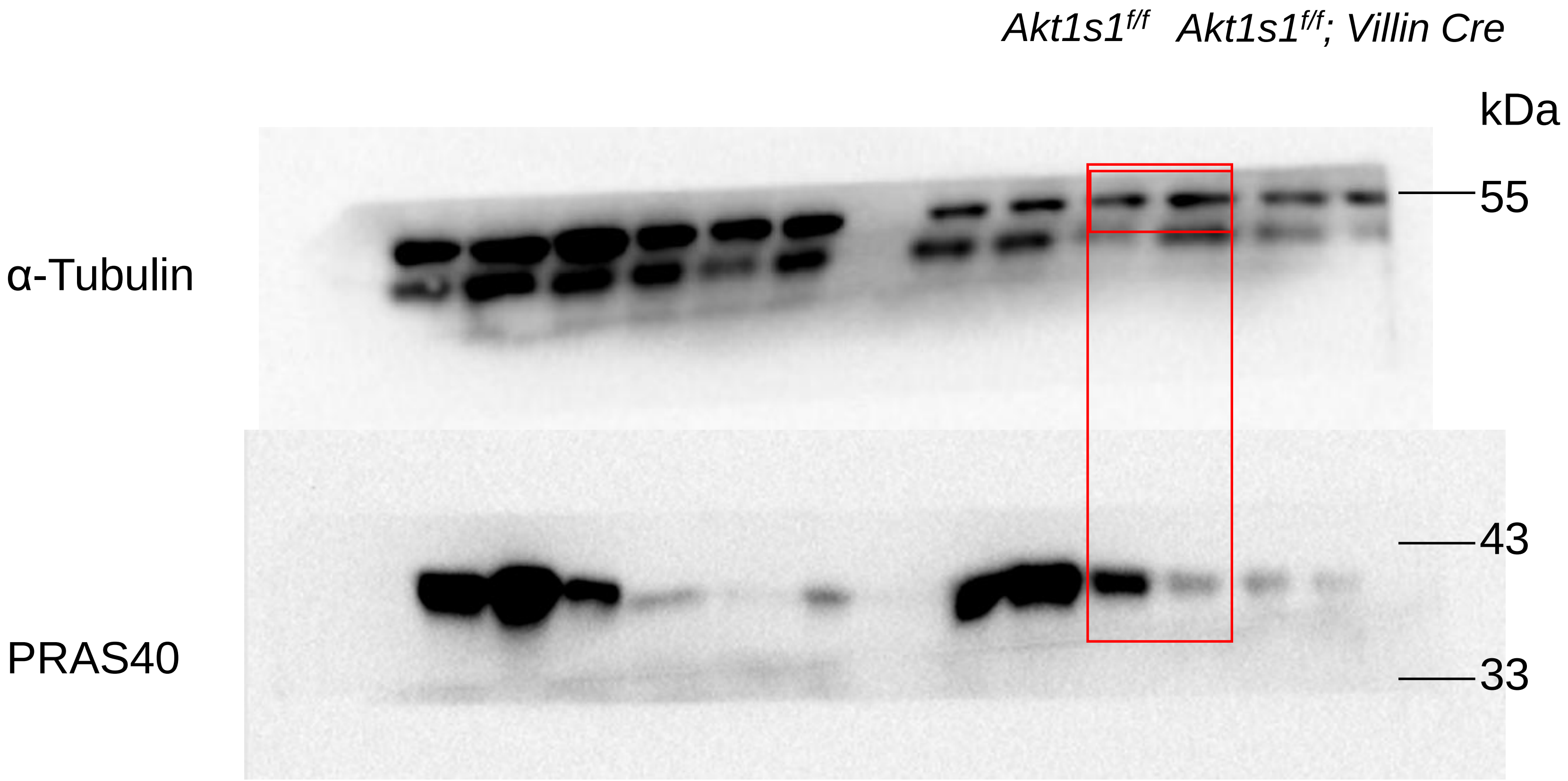

Supplementary Fig. 8E

Correlated to Fig. 2H

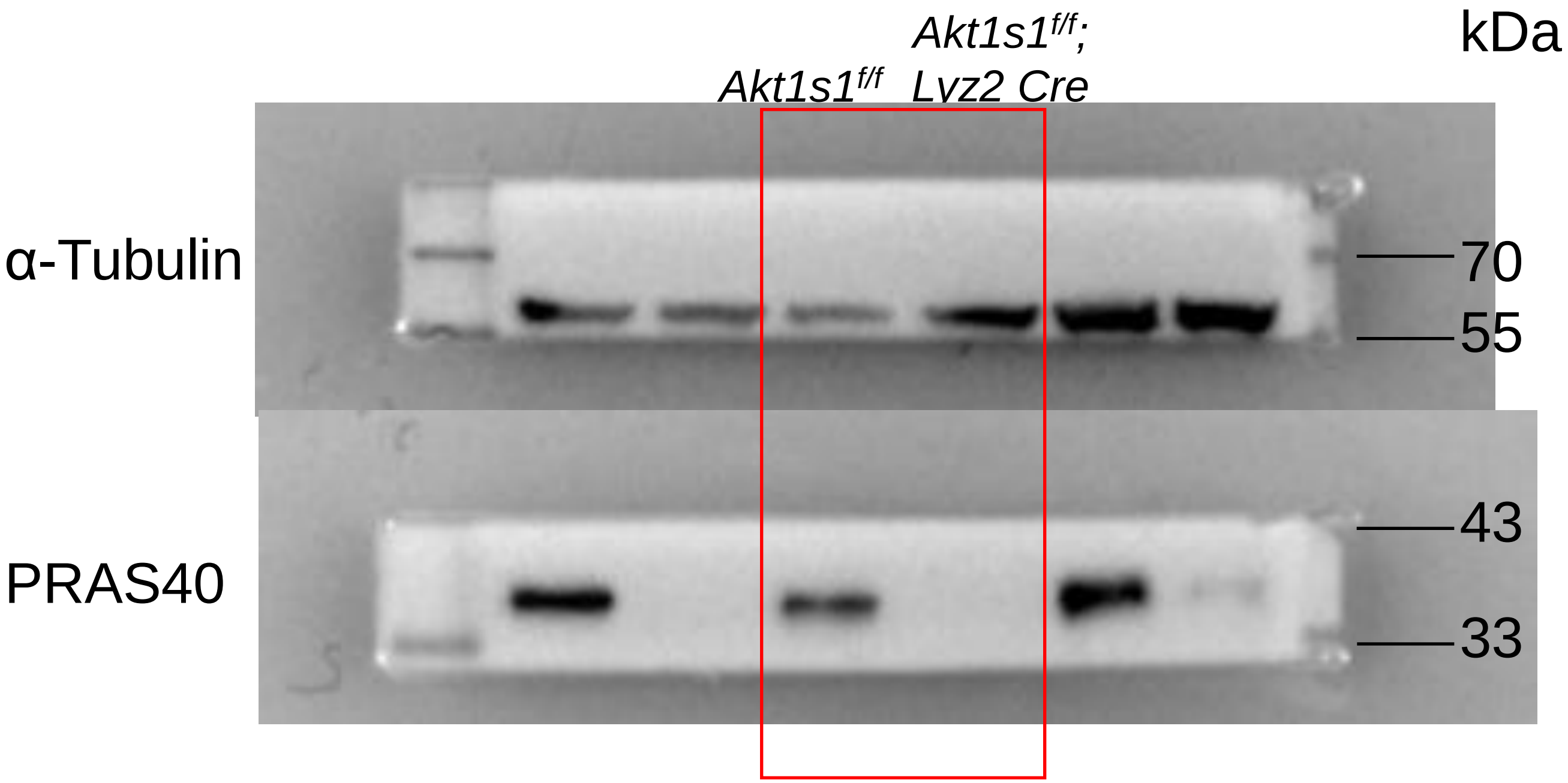

Supplementary Fig. 8F

Correlated to Fig. S3A

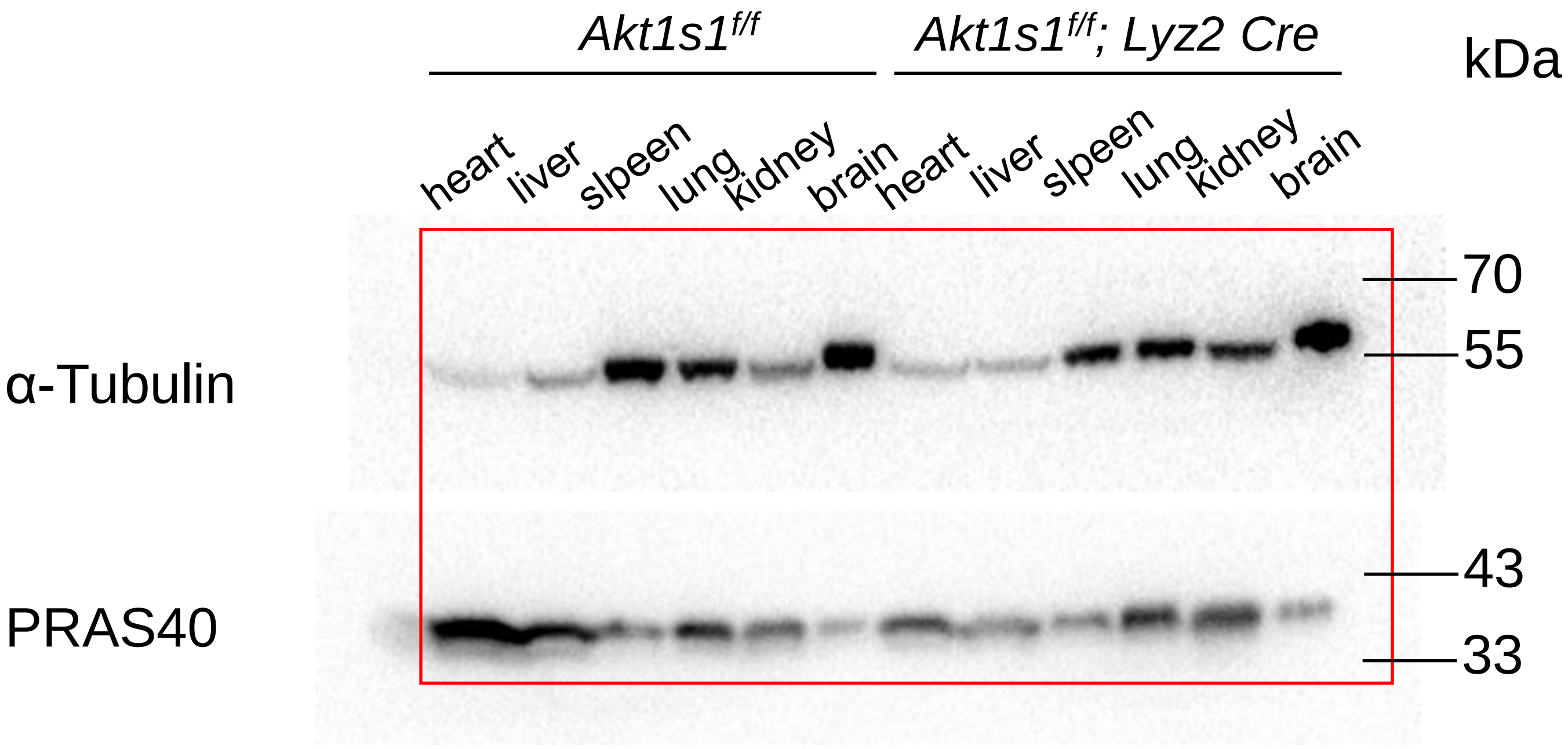

Supplementary Fig. 8G

Correlated to Fig. 2M

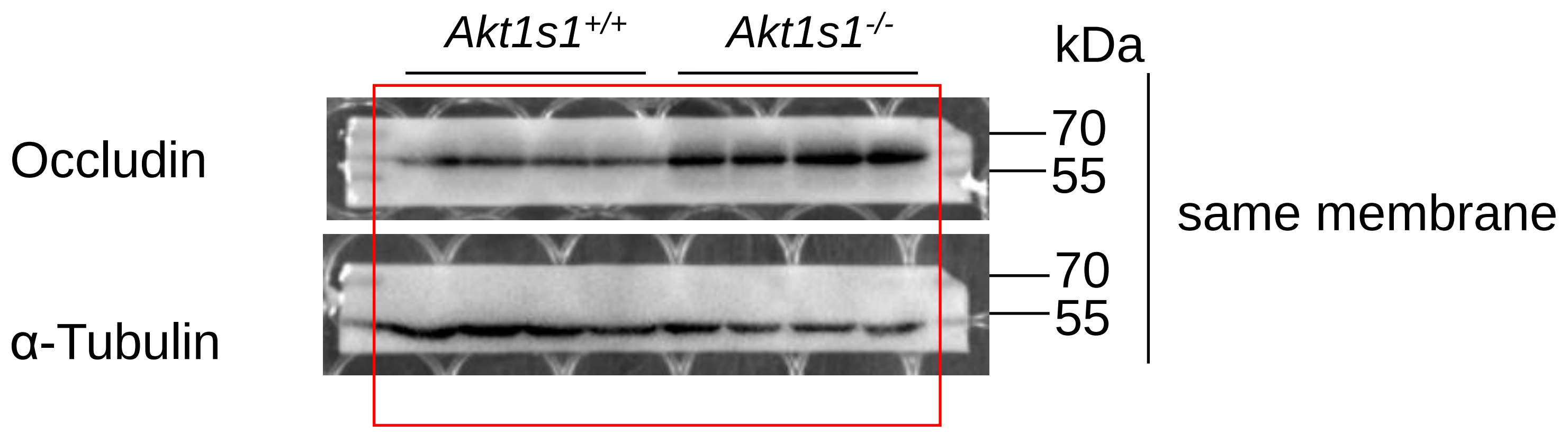

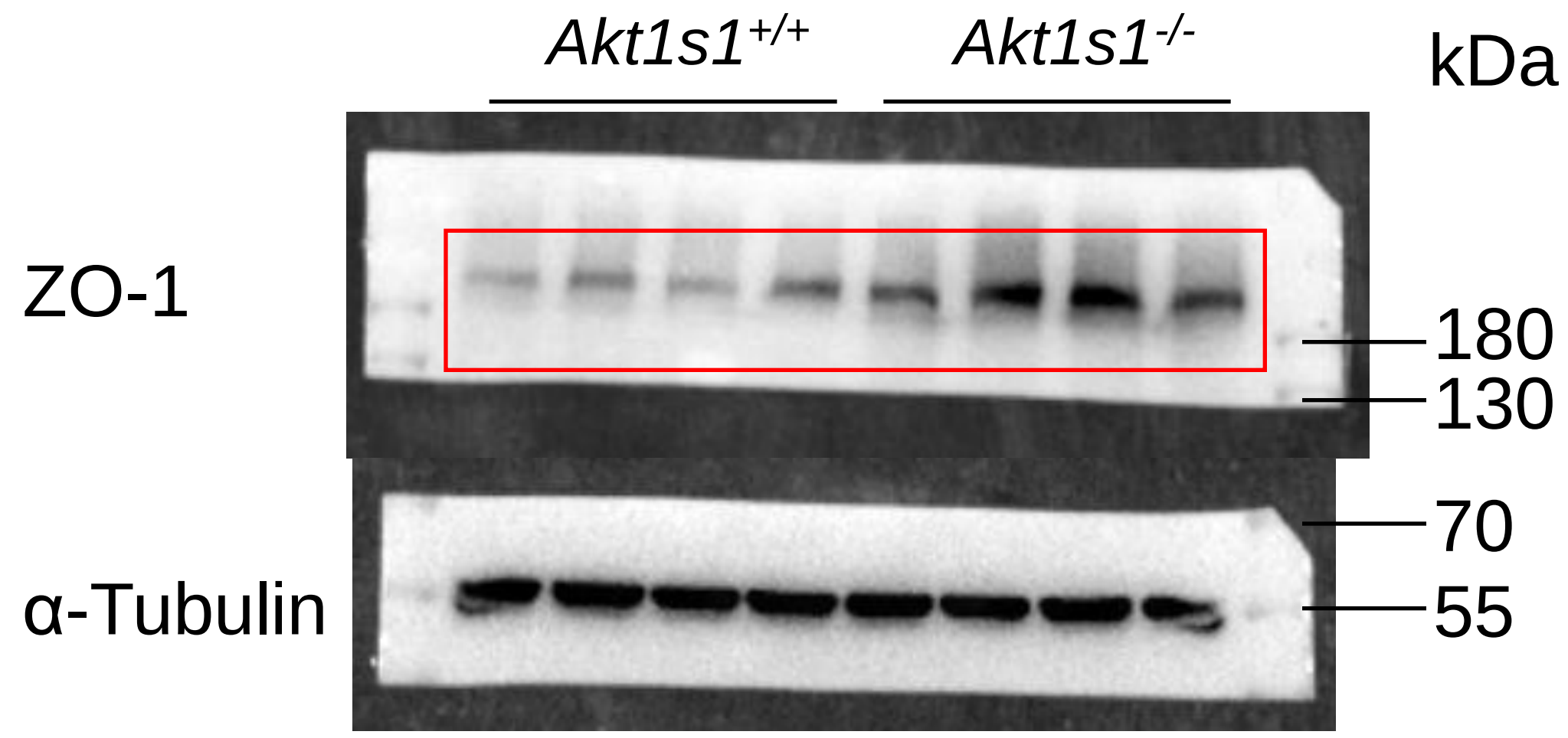

Supplementary Fig. 8H

Correlated to Fig. 3A

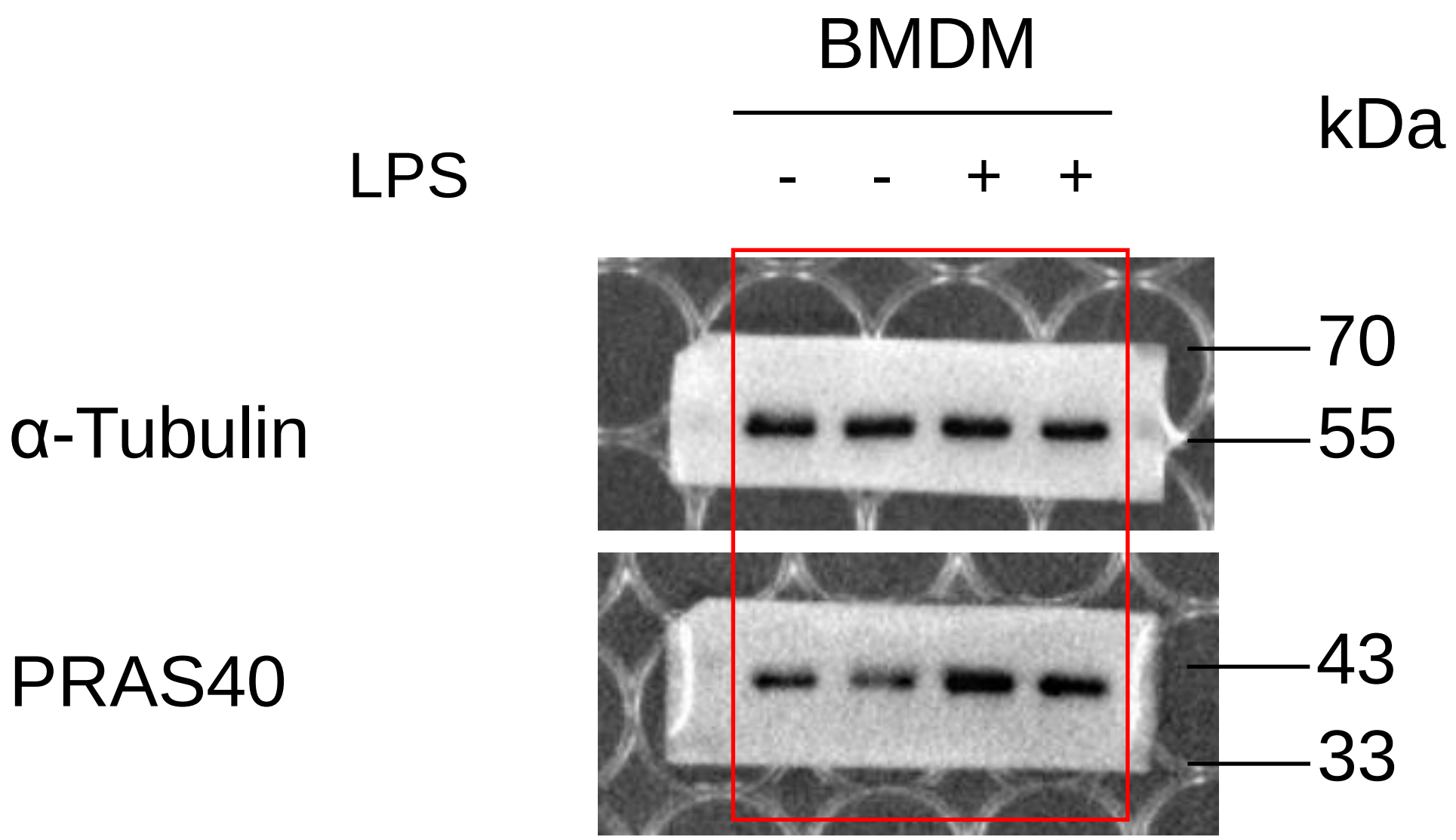

Supplementary Fig. 8I

Correlated to Fig. 3D

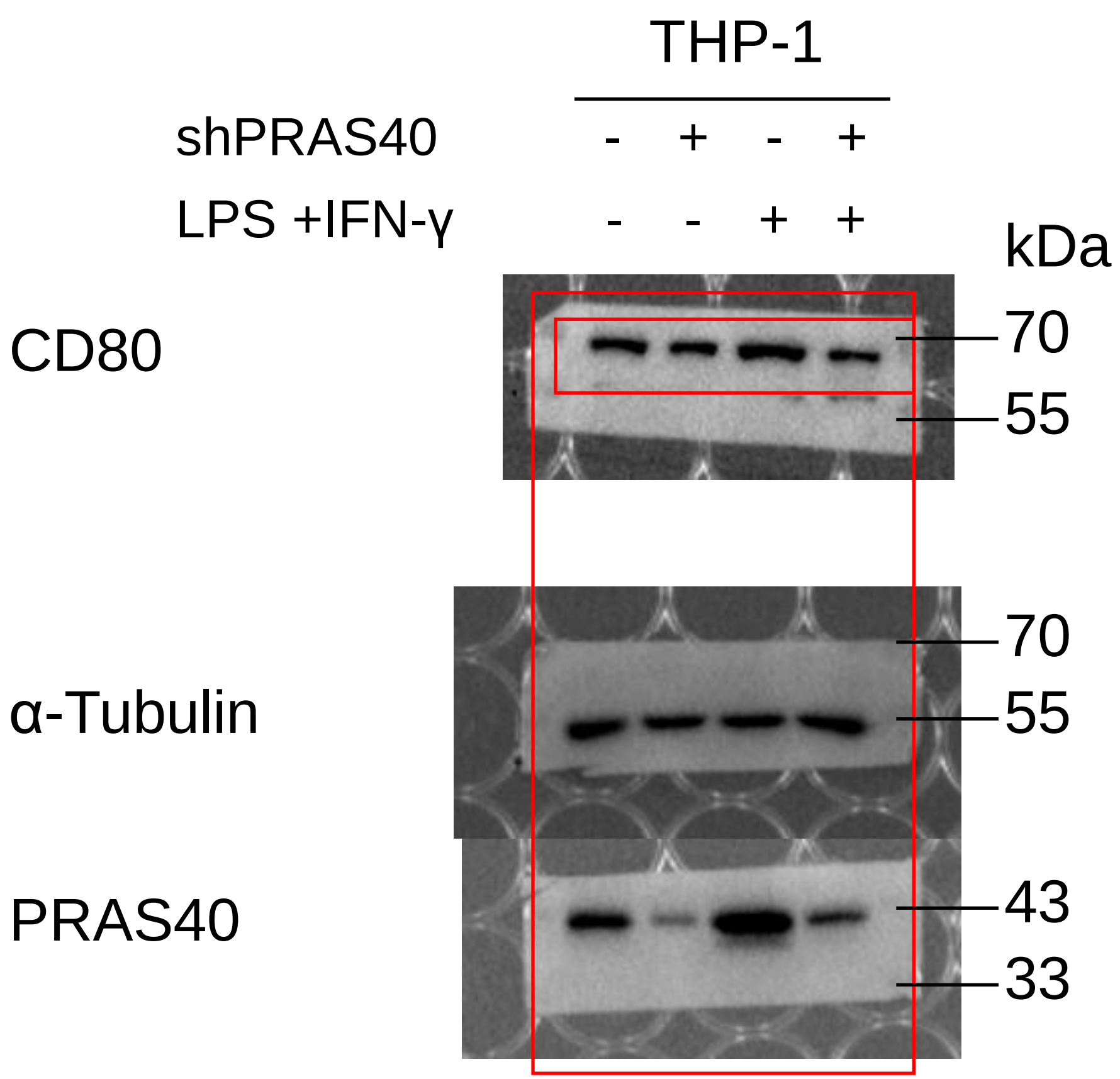

Supplementary Fig. 8J

Correlated to Fig. 3E

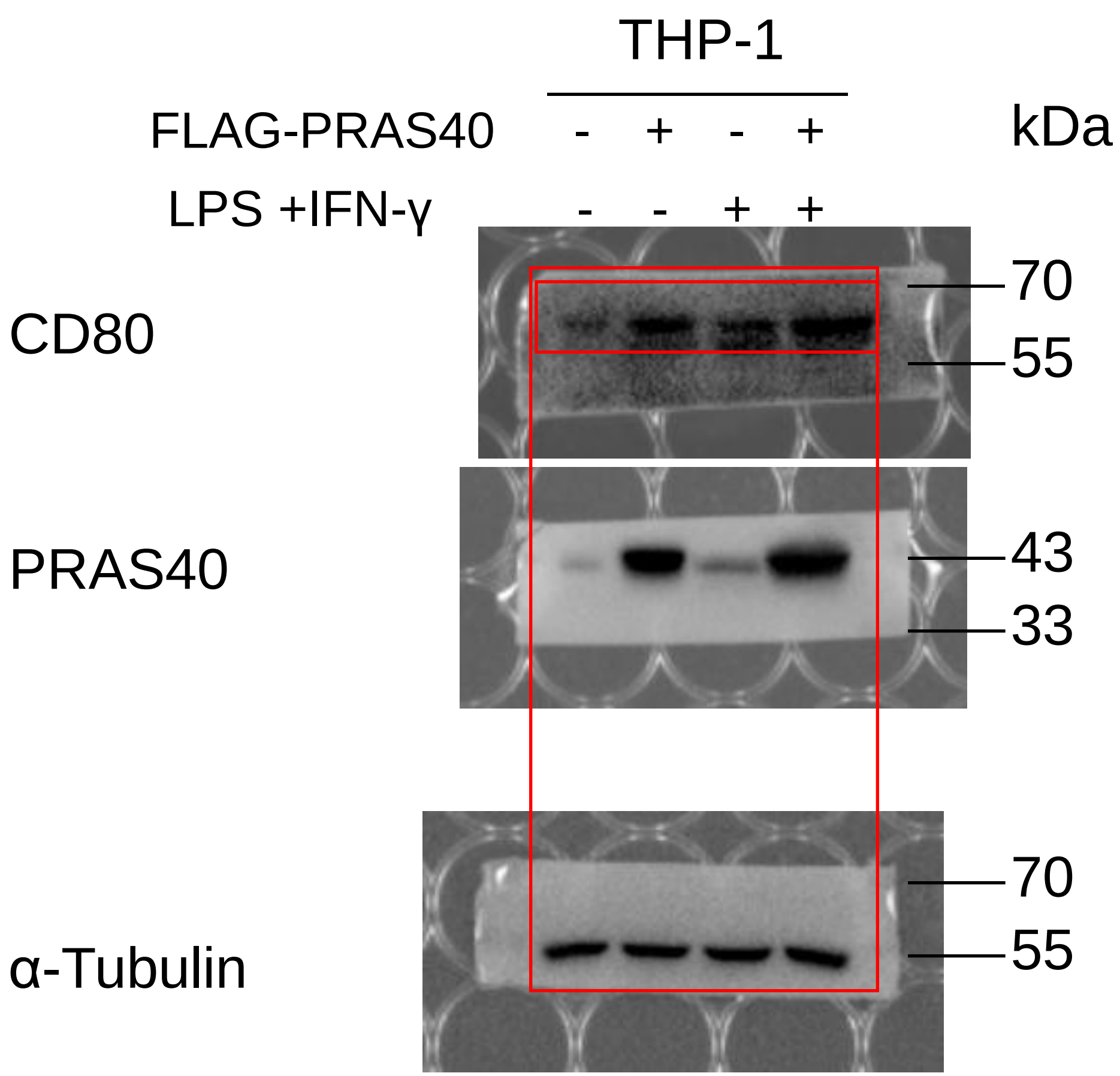

Supplementary Fig. 8K

Correlated to Fig. 3F

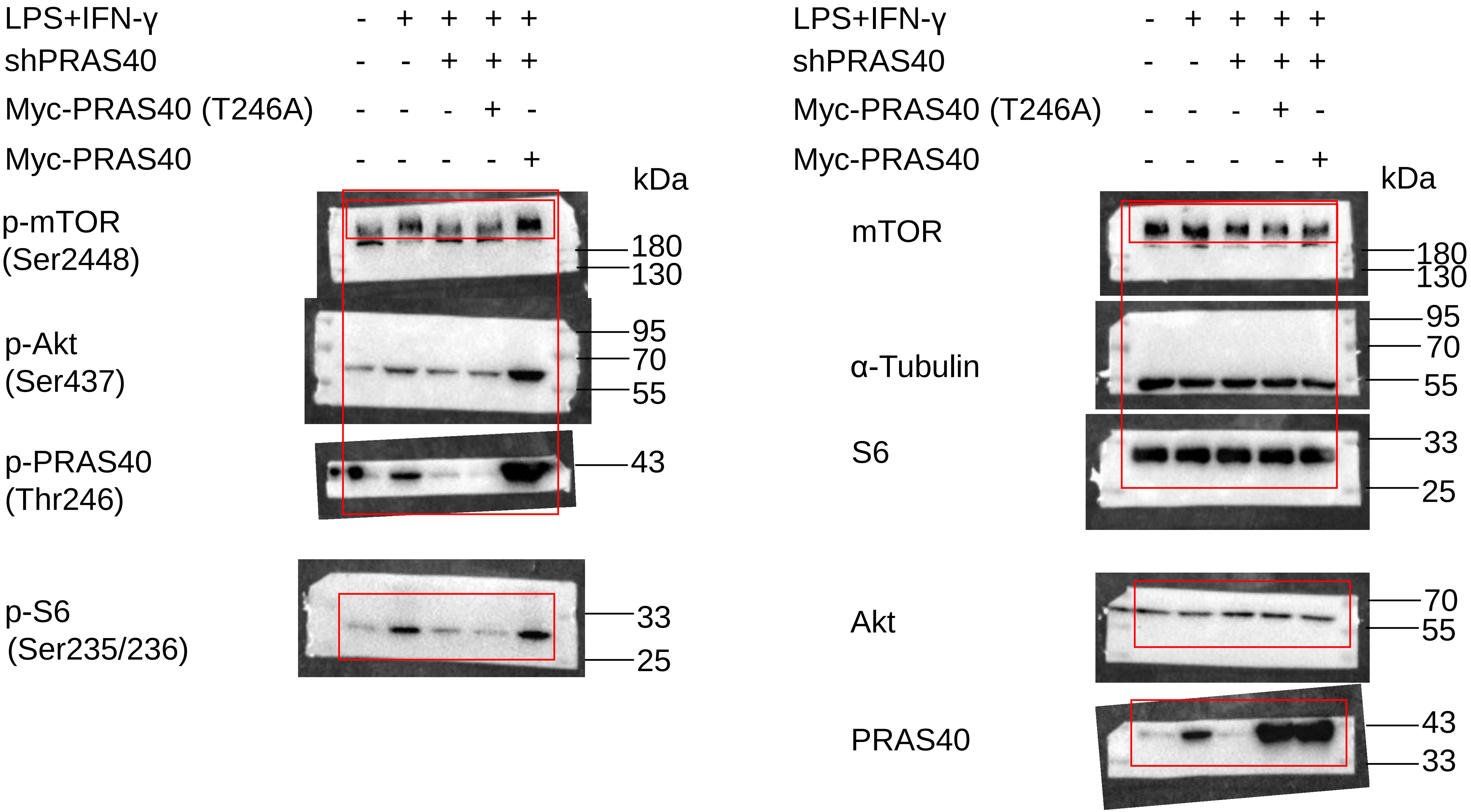

Supplementary Fig. 8L

Correlated to Fig. 4D

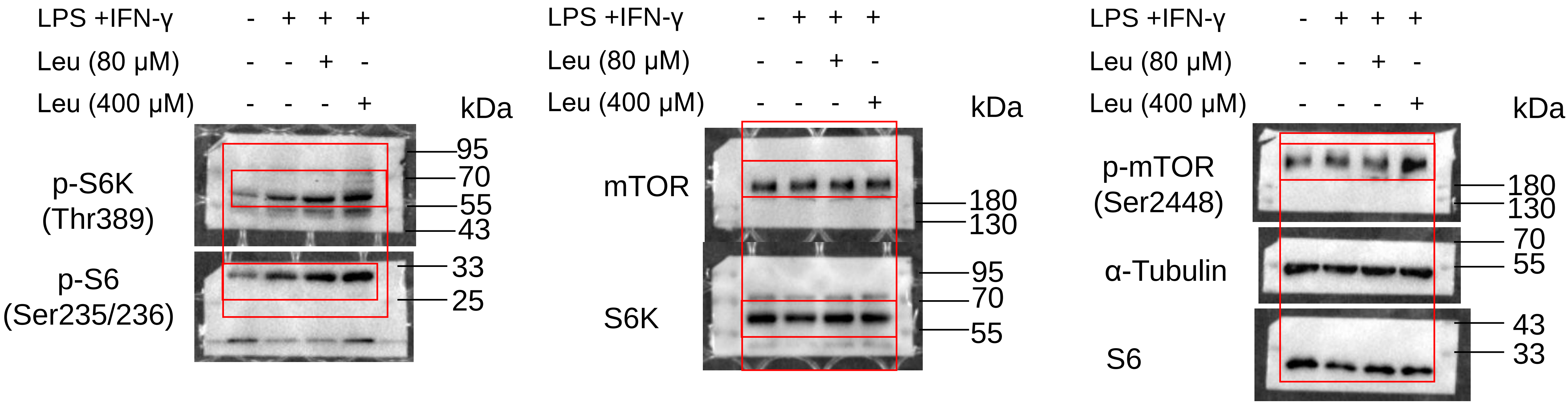

Supplementary Fig. 8M

Correlated to Fig. 4E

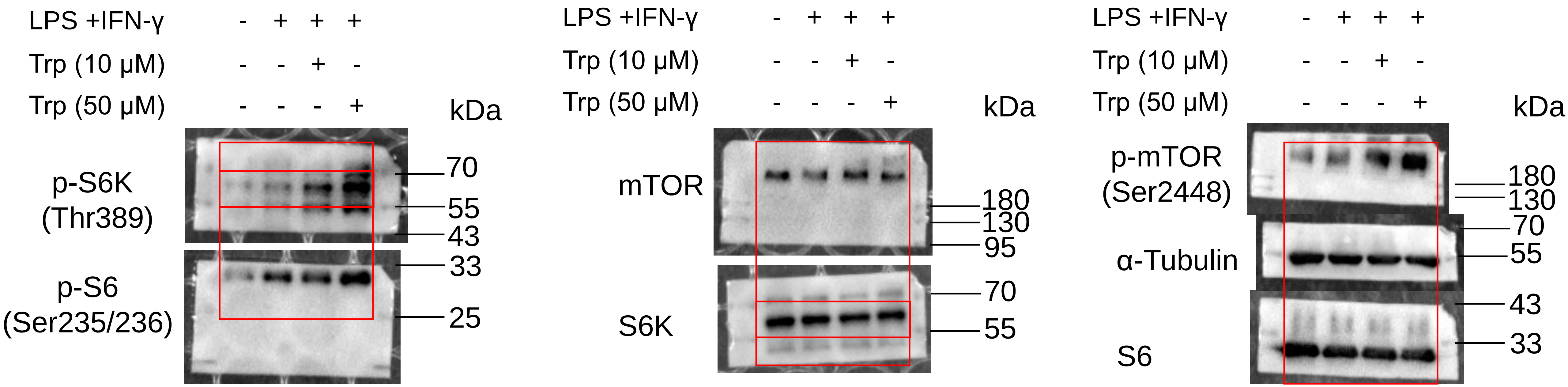

Supplementary Fig. 8N

Correlated to Fig. 4I

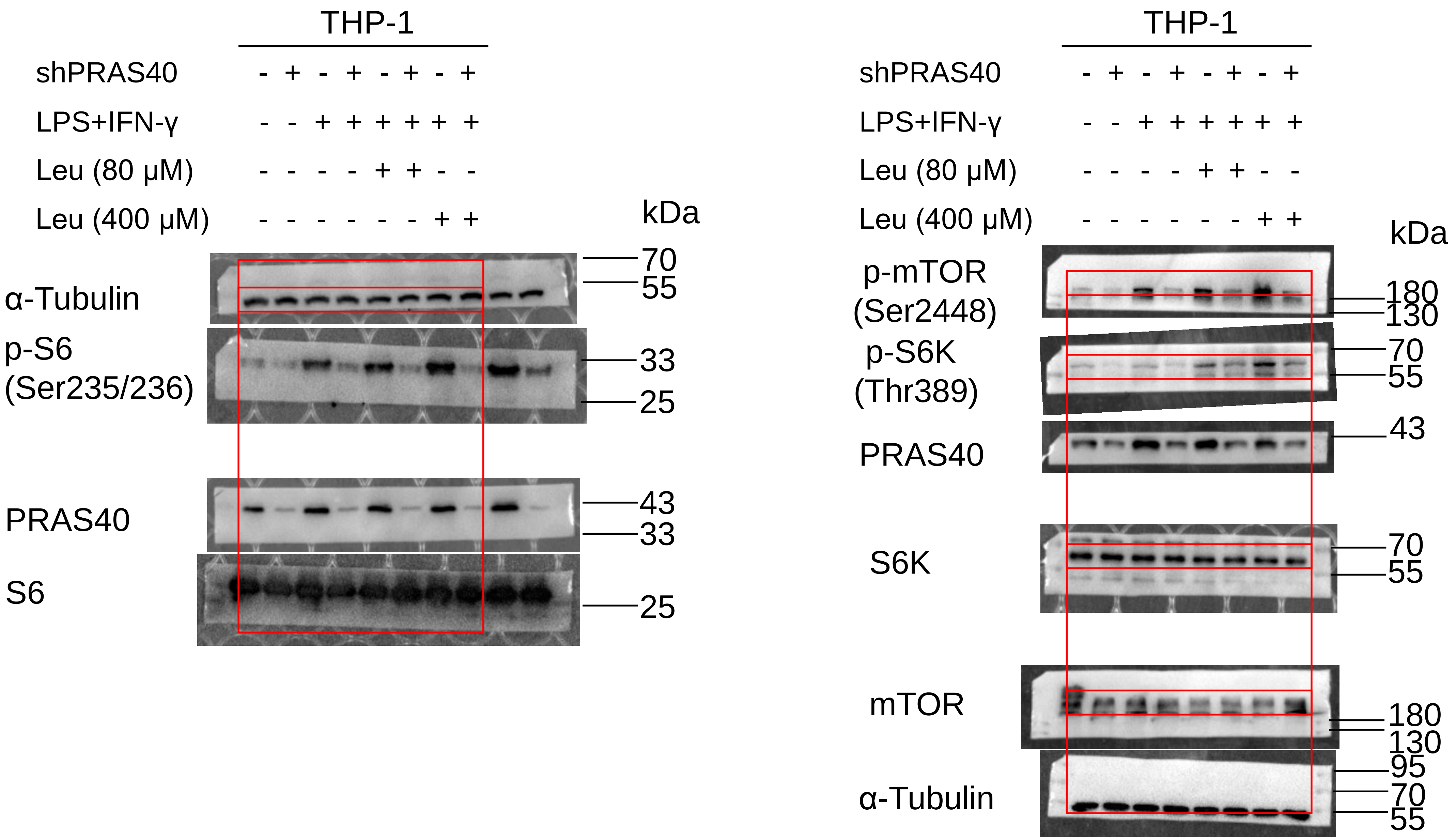

Supplementary Fig. 8O

Correlated to Fig. 4J

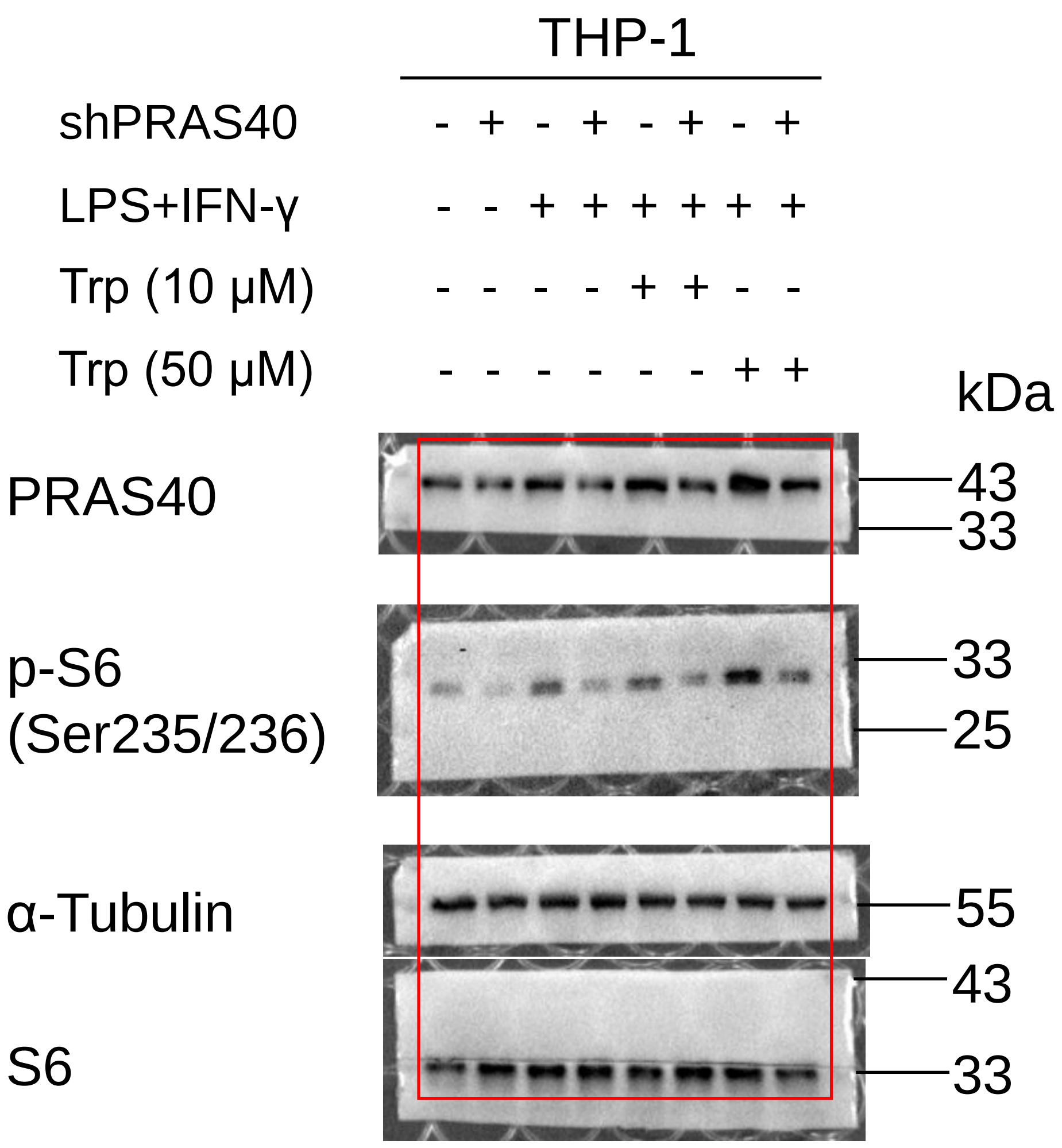

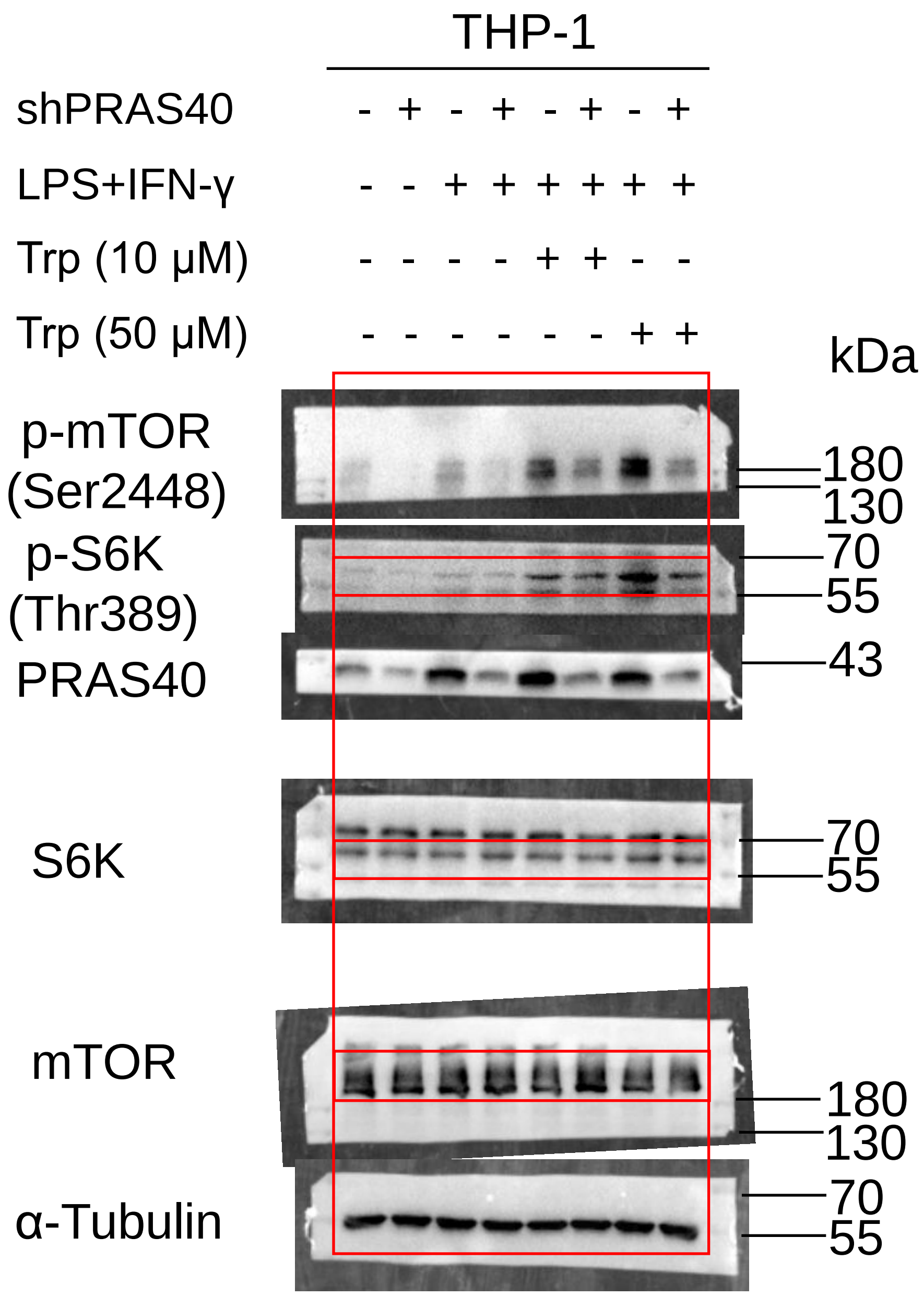

Supplementary Fig. 8P

Correlated to Fig. 5E

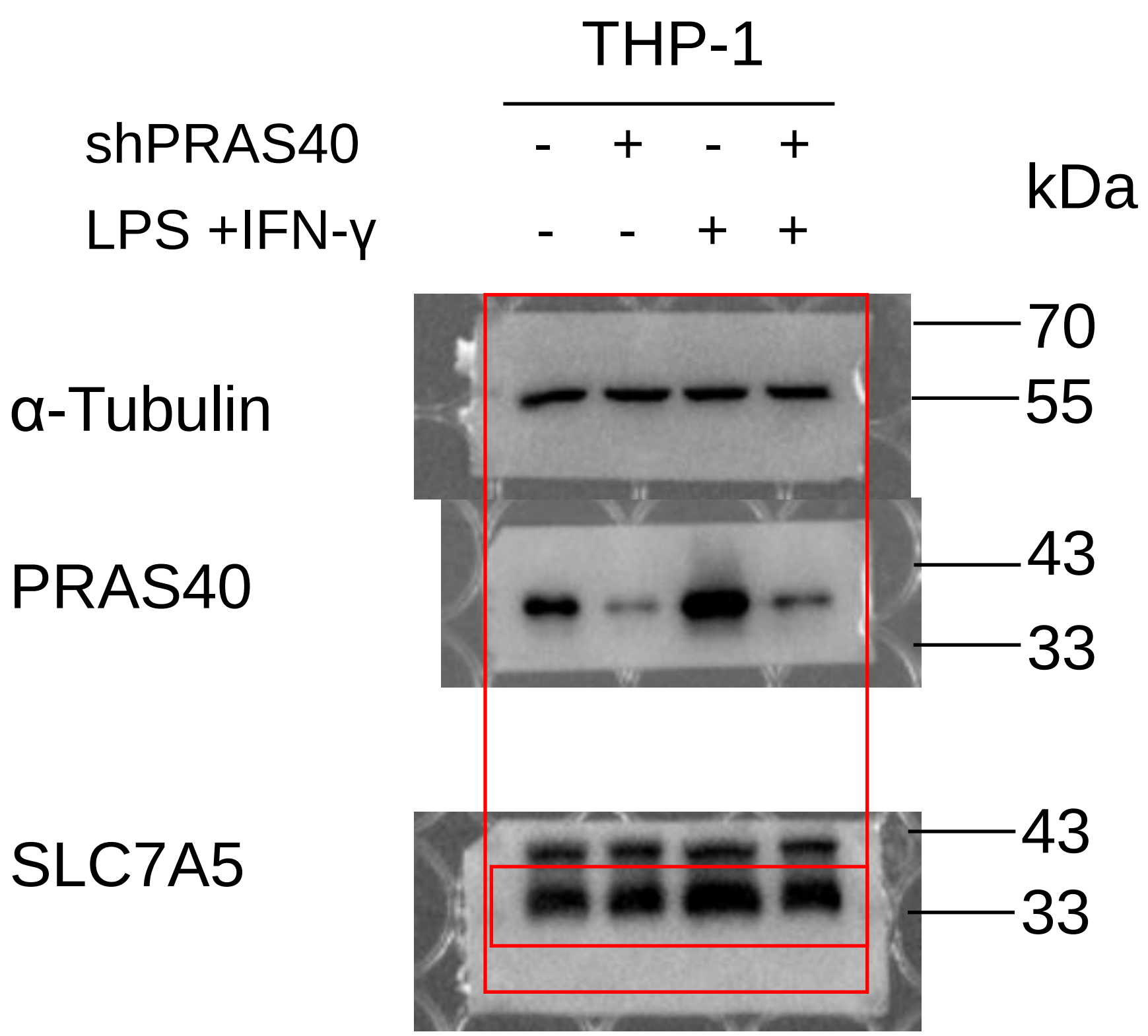

Supplementary Fig. 8Q

Correlated to Fig. 5F

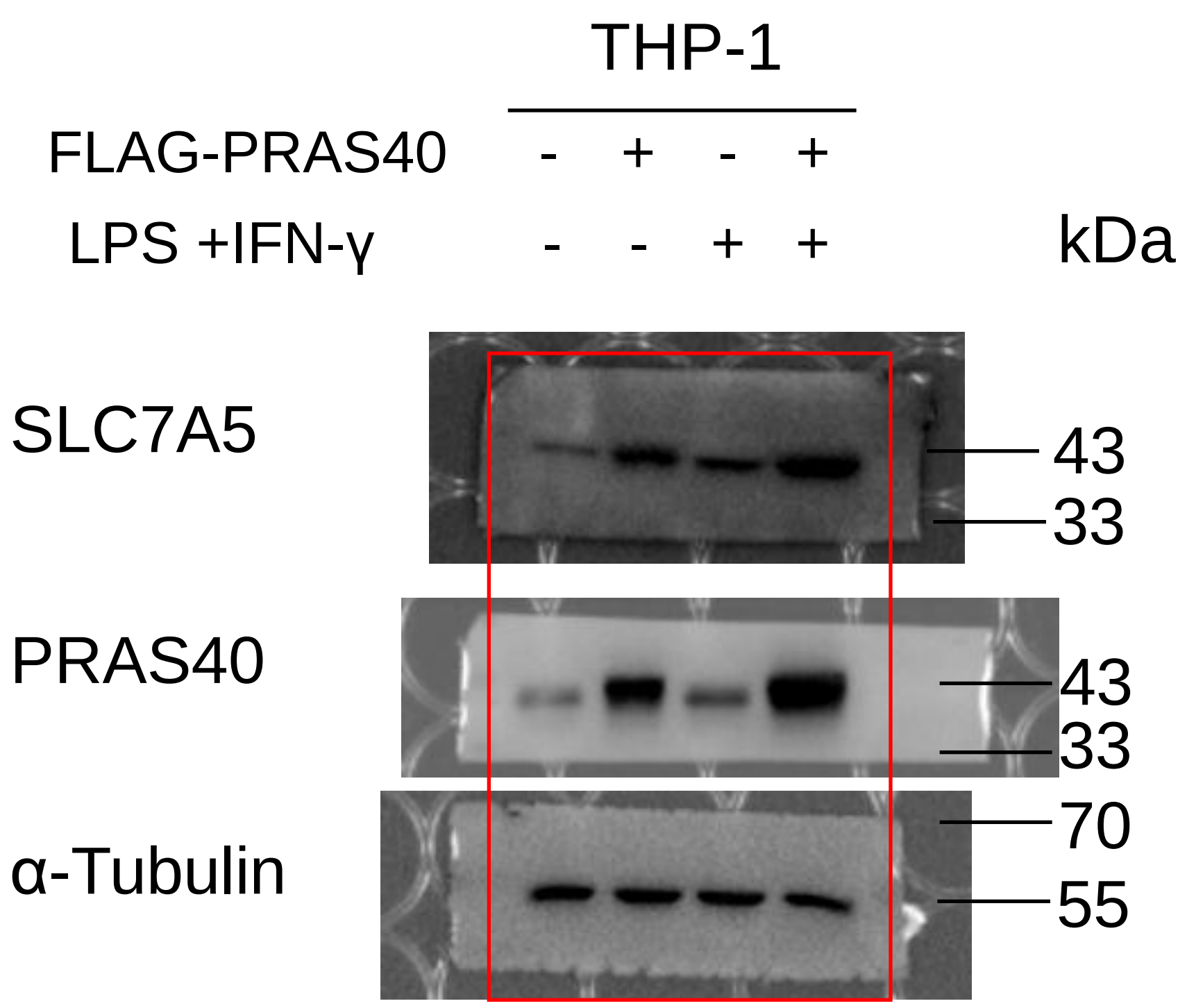

Supplementary Fig. 8R

Correlated to Fig. 5I

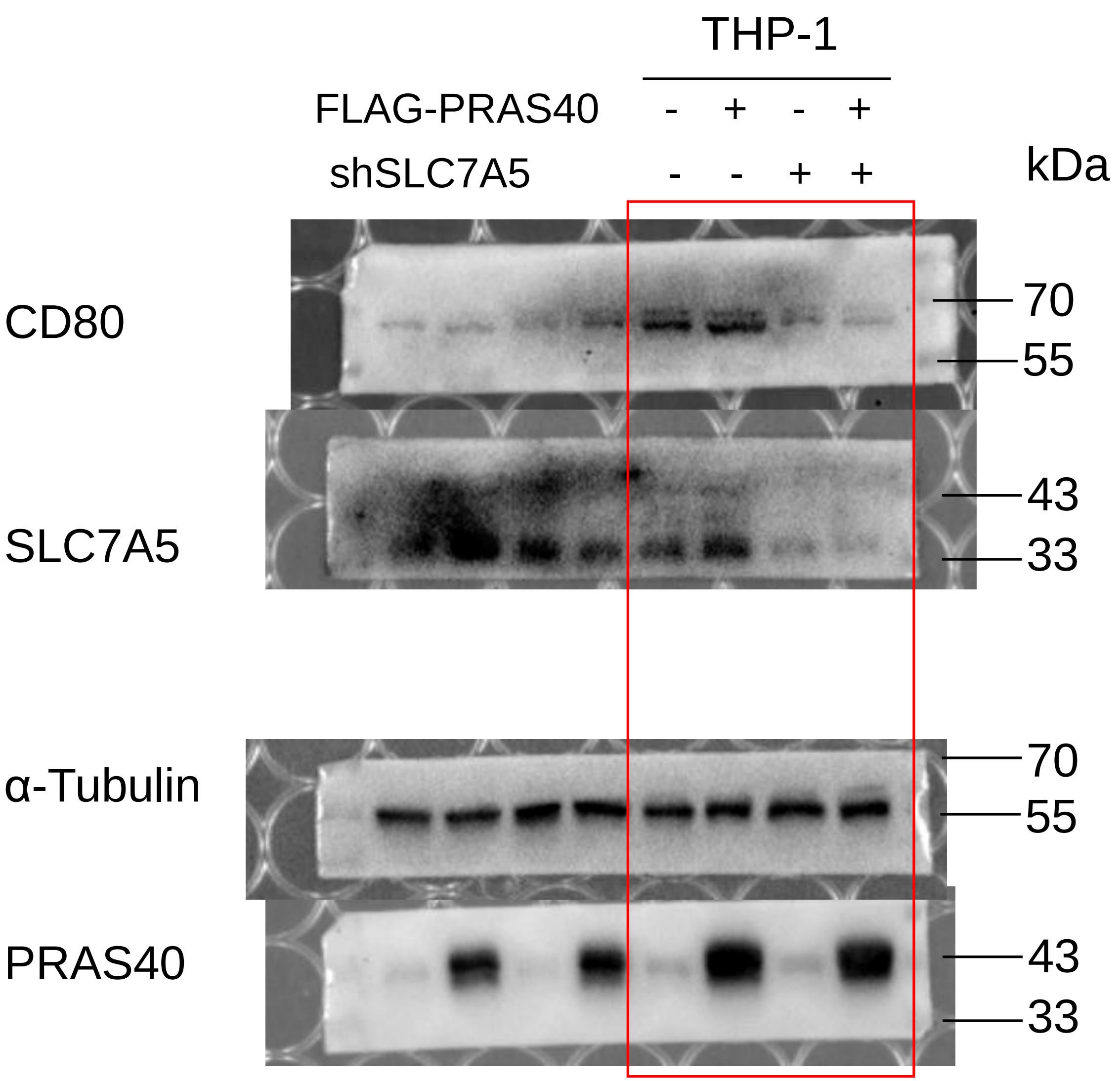

Supplementary Fig. 8S

Correlated to Fig. S6C

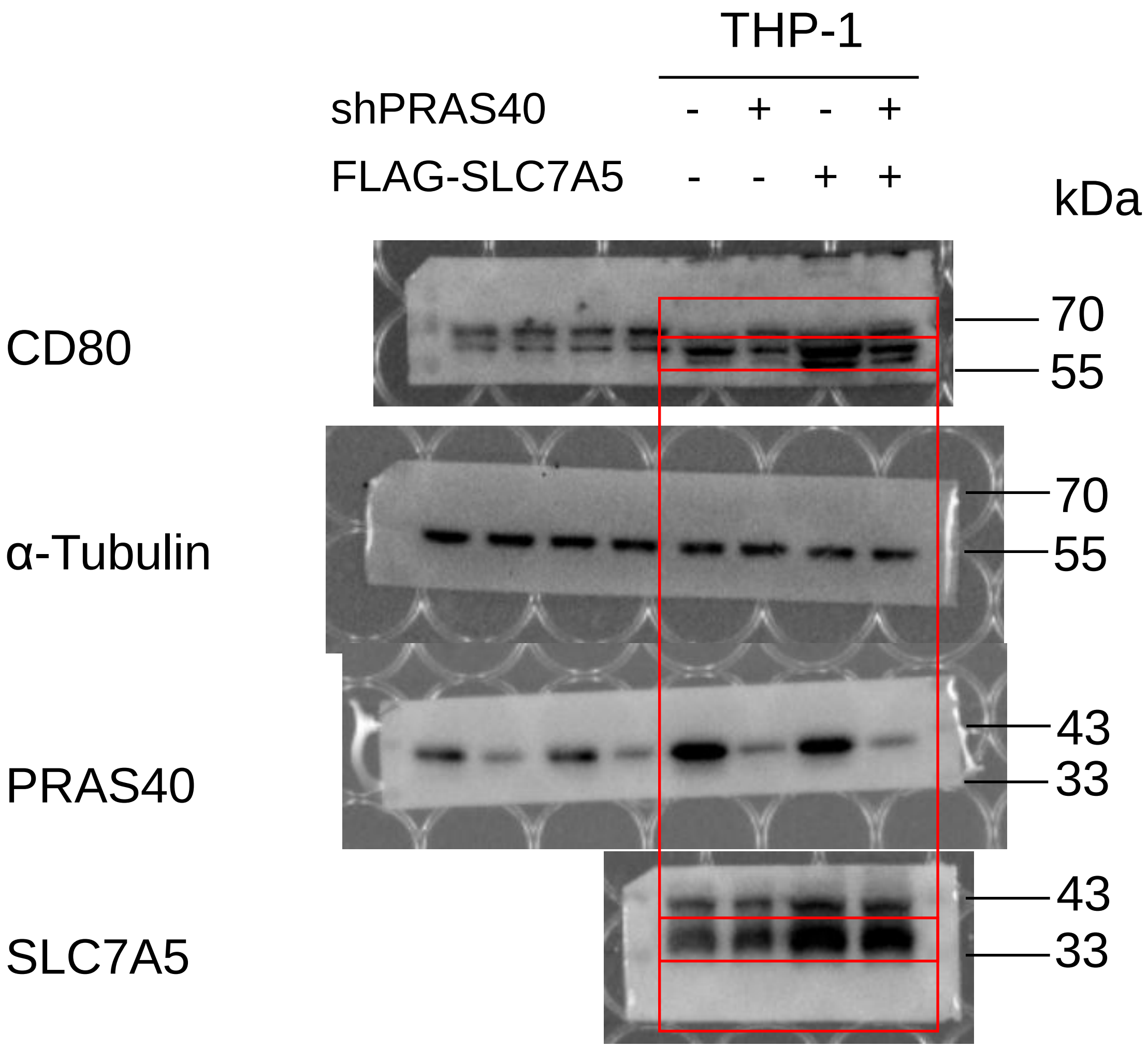

Supplementary Fig. 8T

Correlated to Fig. 6H

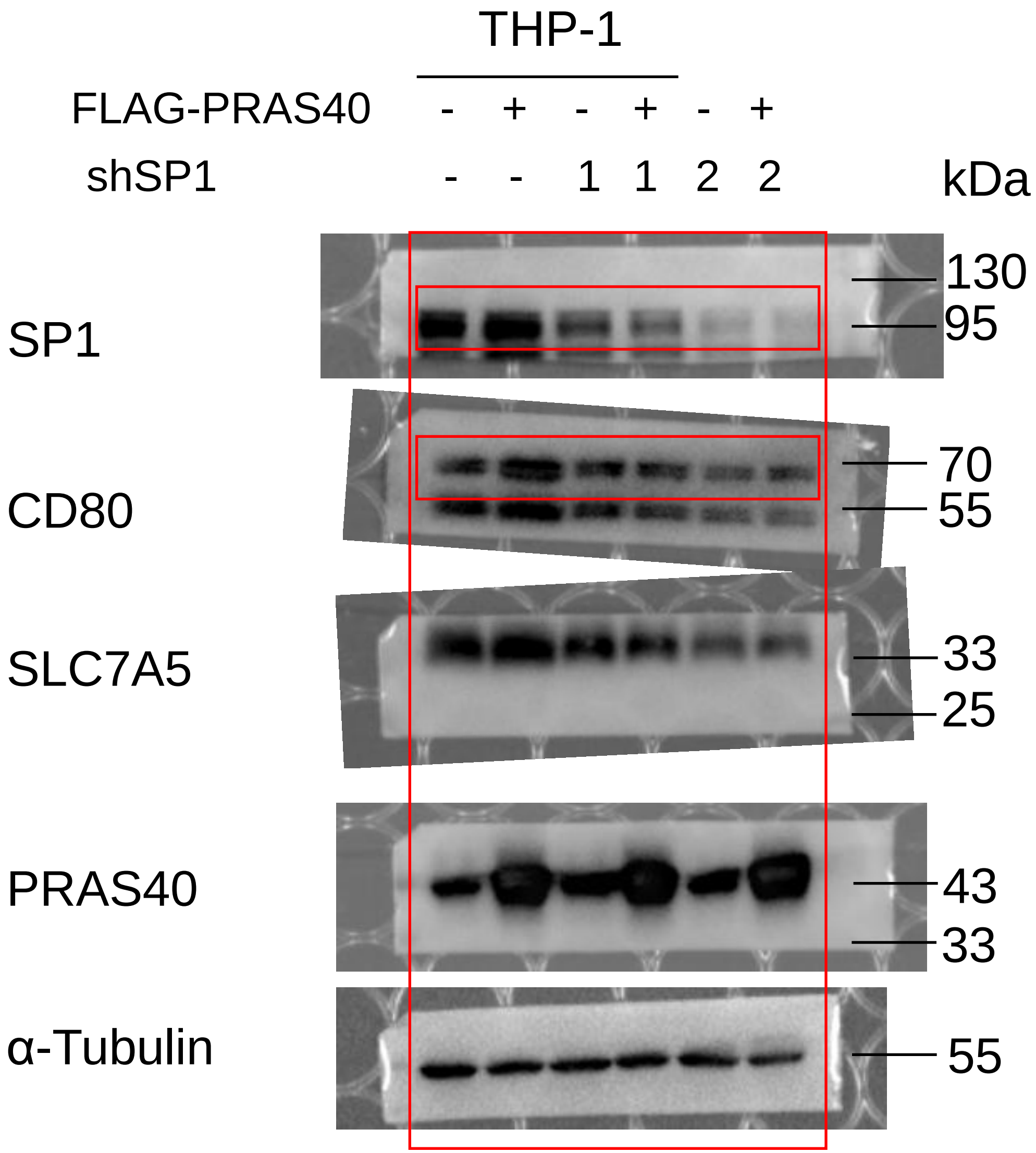

Supplementary Fig. 8U

Correlated to Fig. S7A

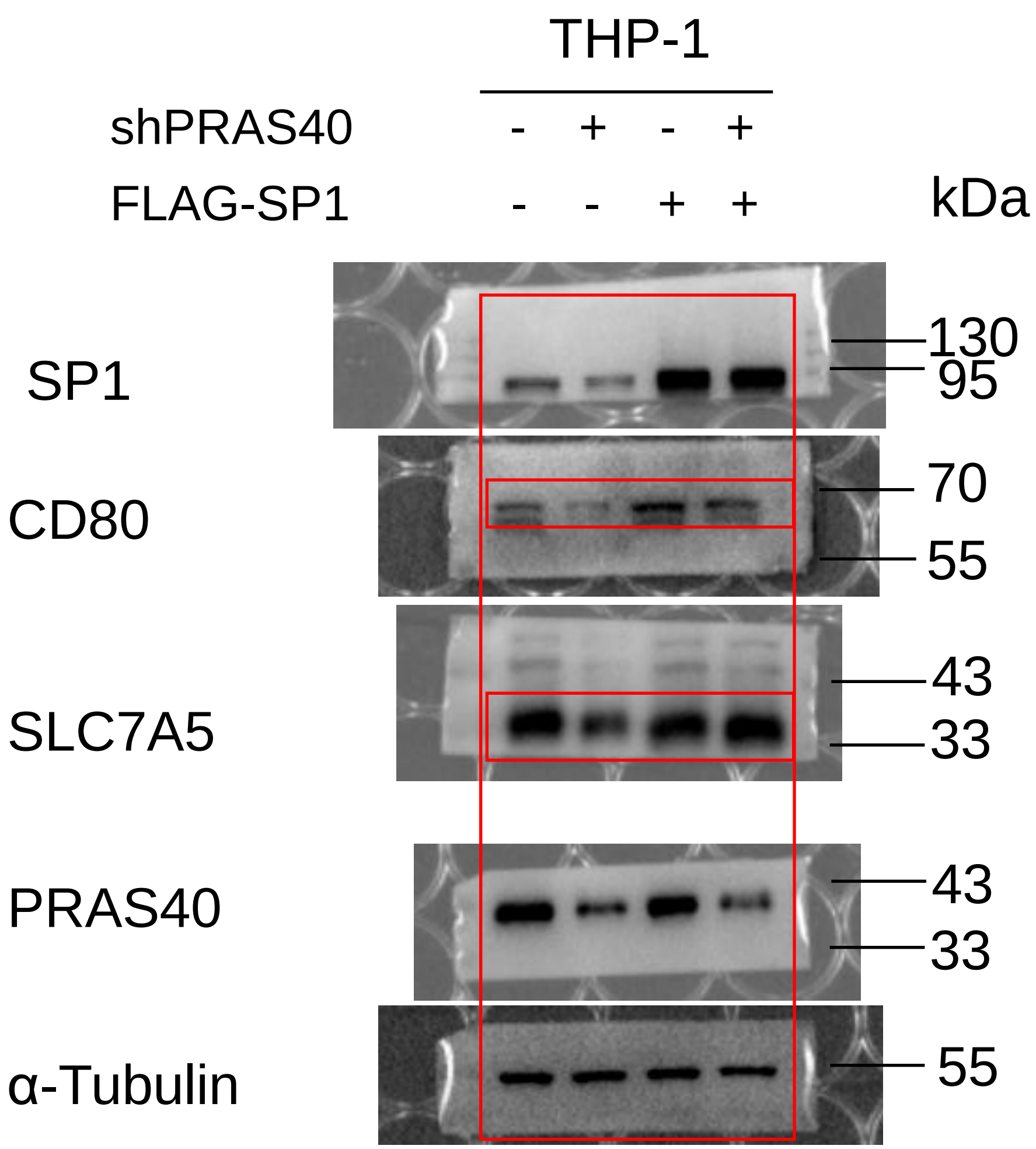

Supplementary Fig. 8V

Correlated to Fig. 7C

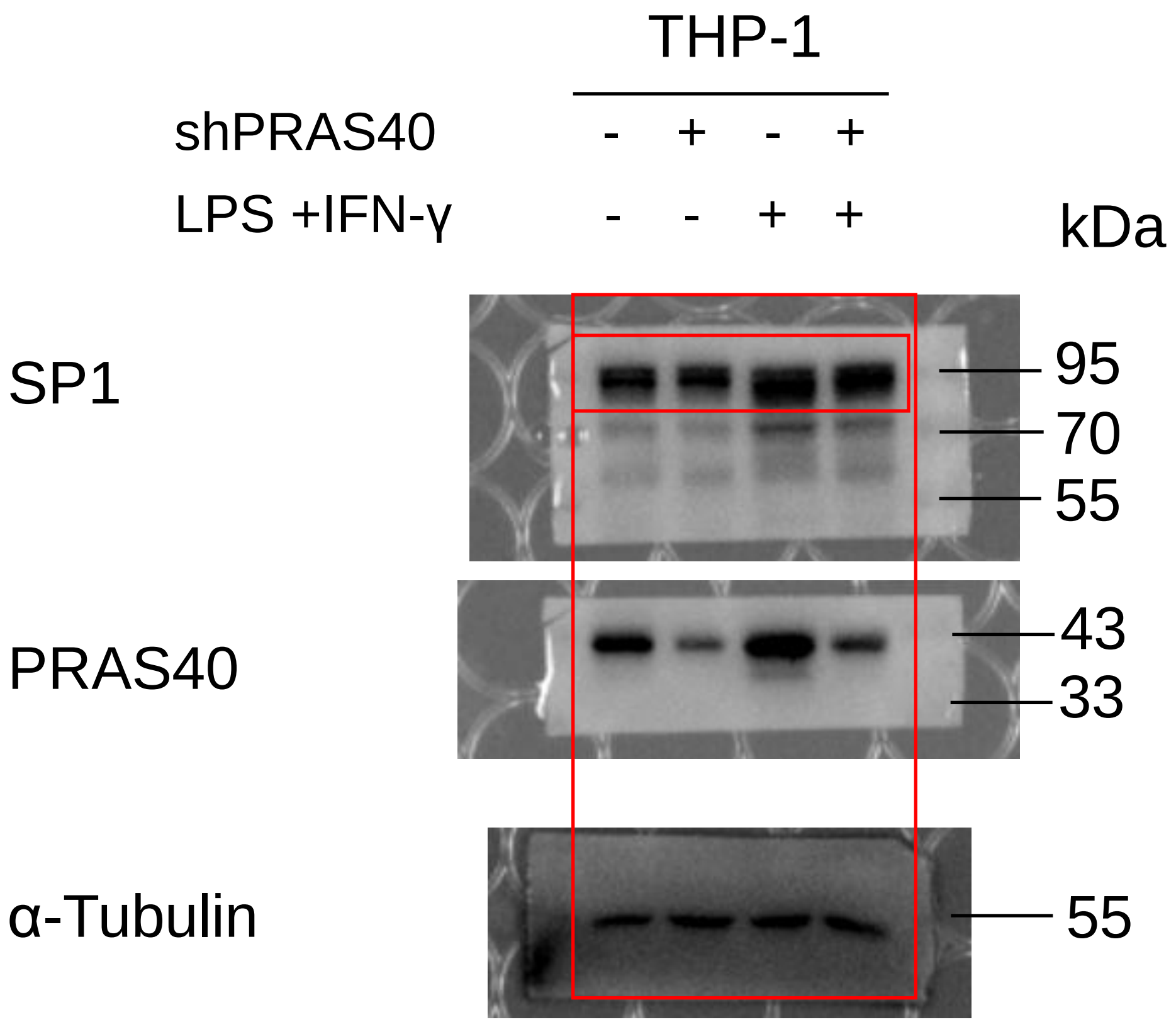

Supplementary Fig. 8W

Correlated to Fig. 7D

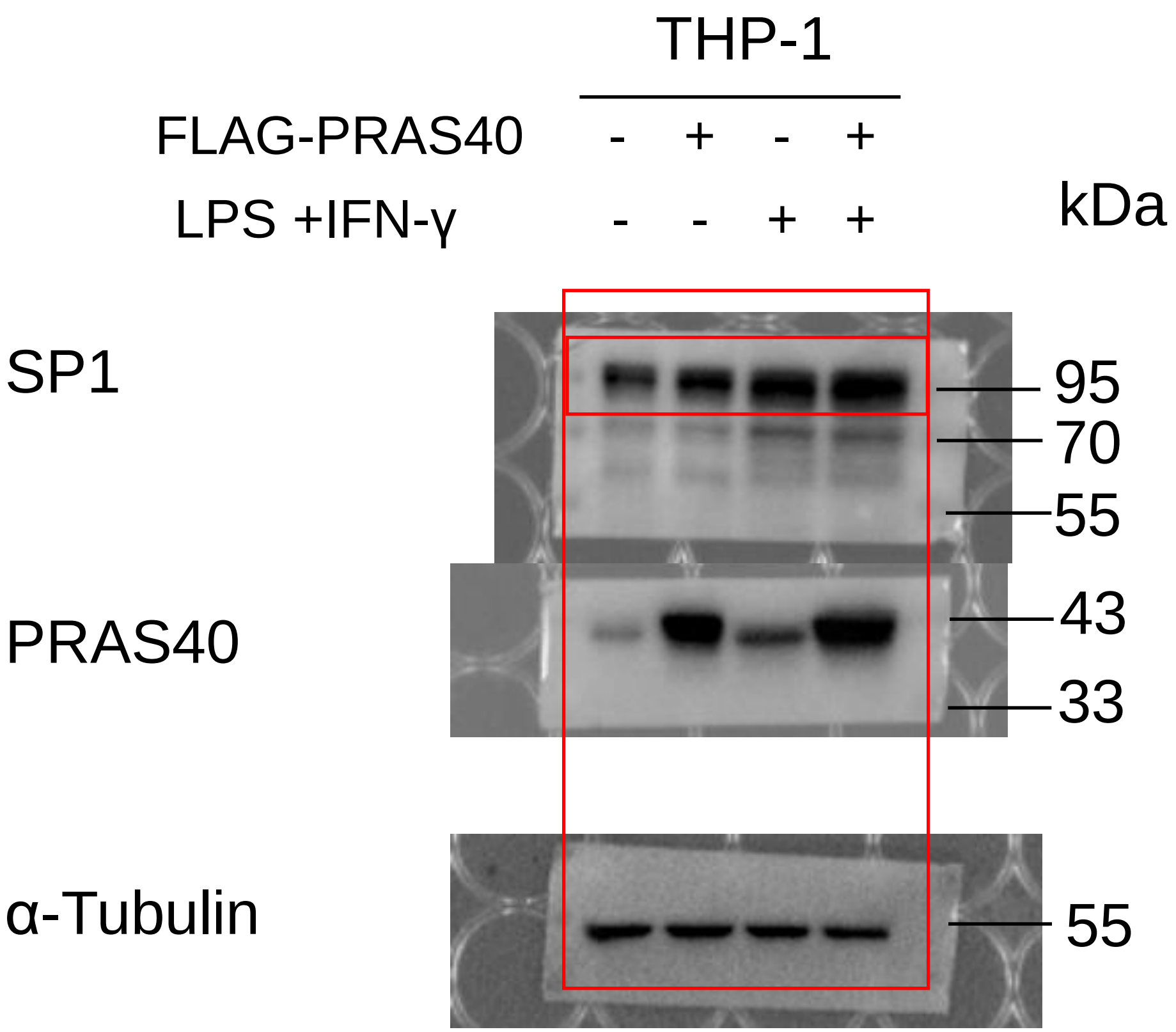

Supplementary Fig. 8X

Correlated to Fig. 7F

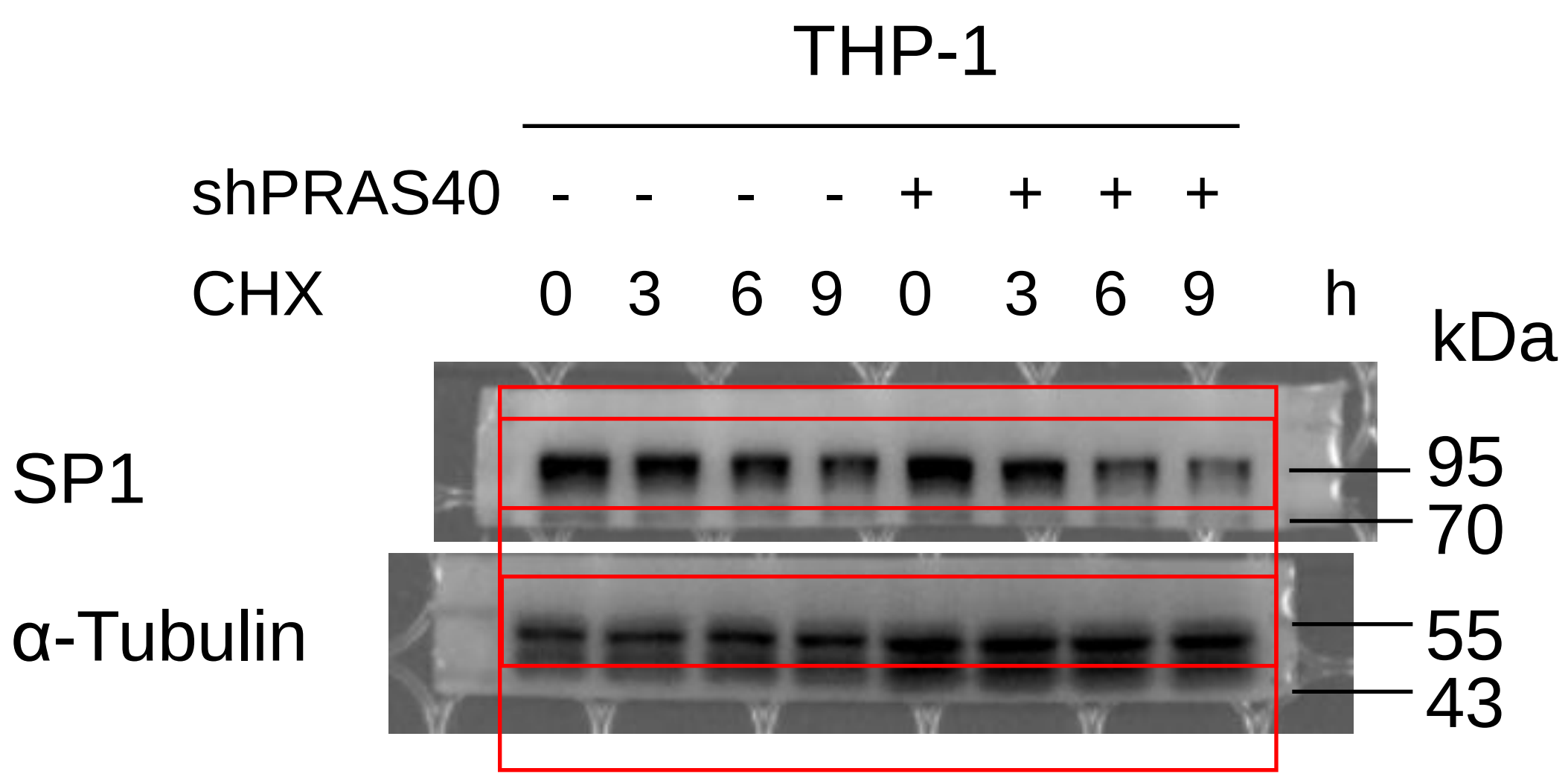

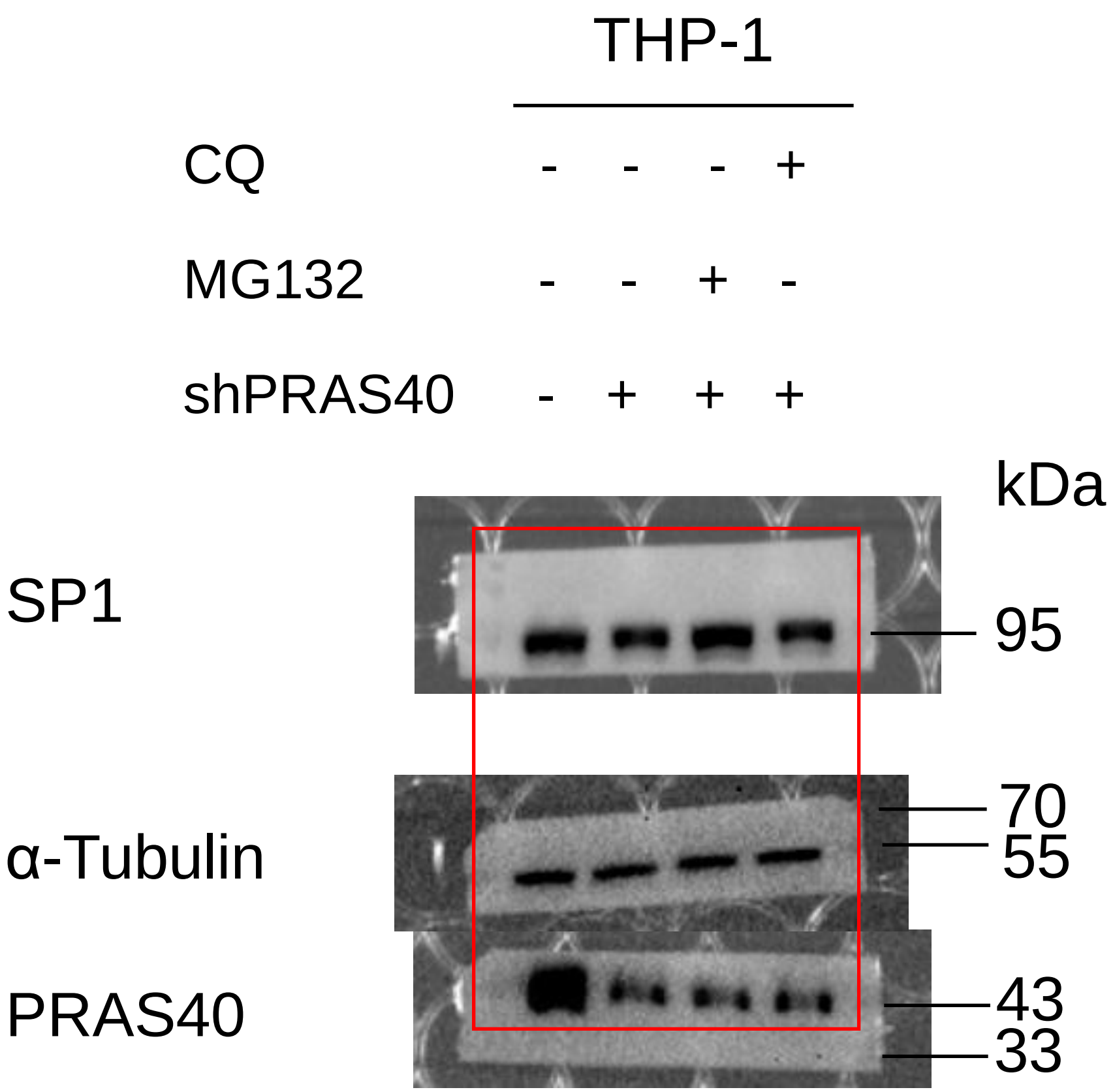
THP-1
CQ - - - +
MG132 - - + -
shPRAS40 - + + +
kDa
SP1
95
α-Tubulin
70
55
PRAS40
43
33

Supplementary Fig. 8Z

Correlated to Fig. 7H

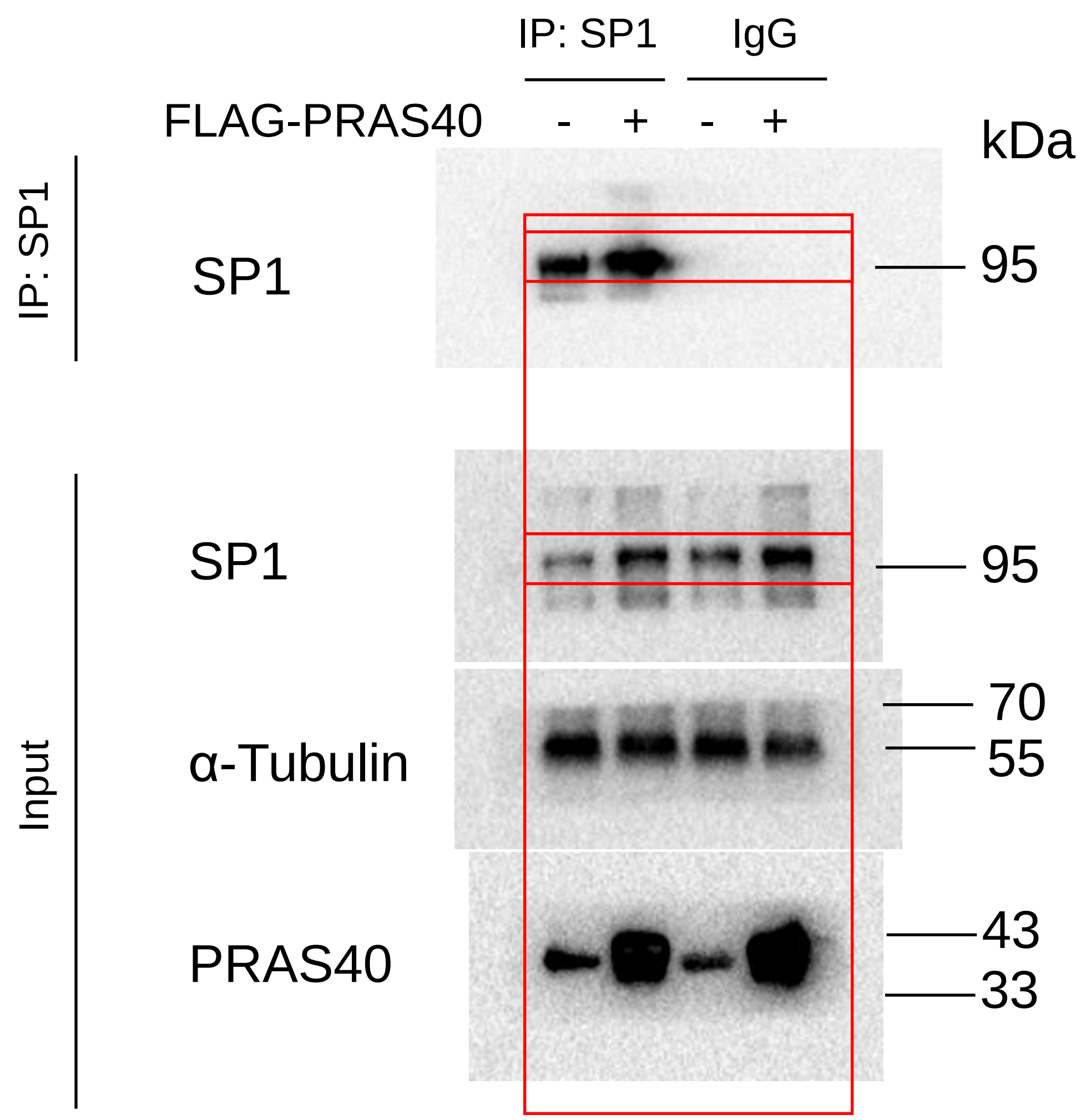

Supplementary Fig. 8AA

Correlated to Fig. 7I

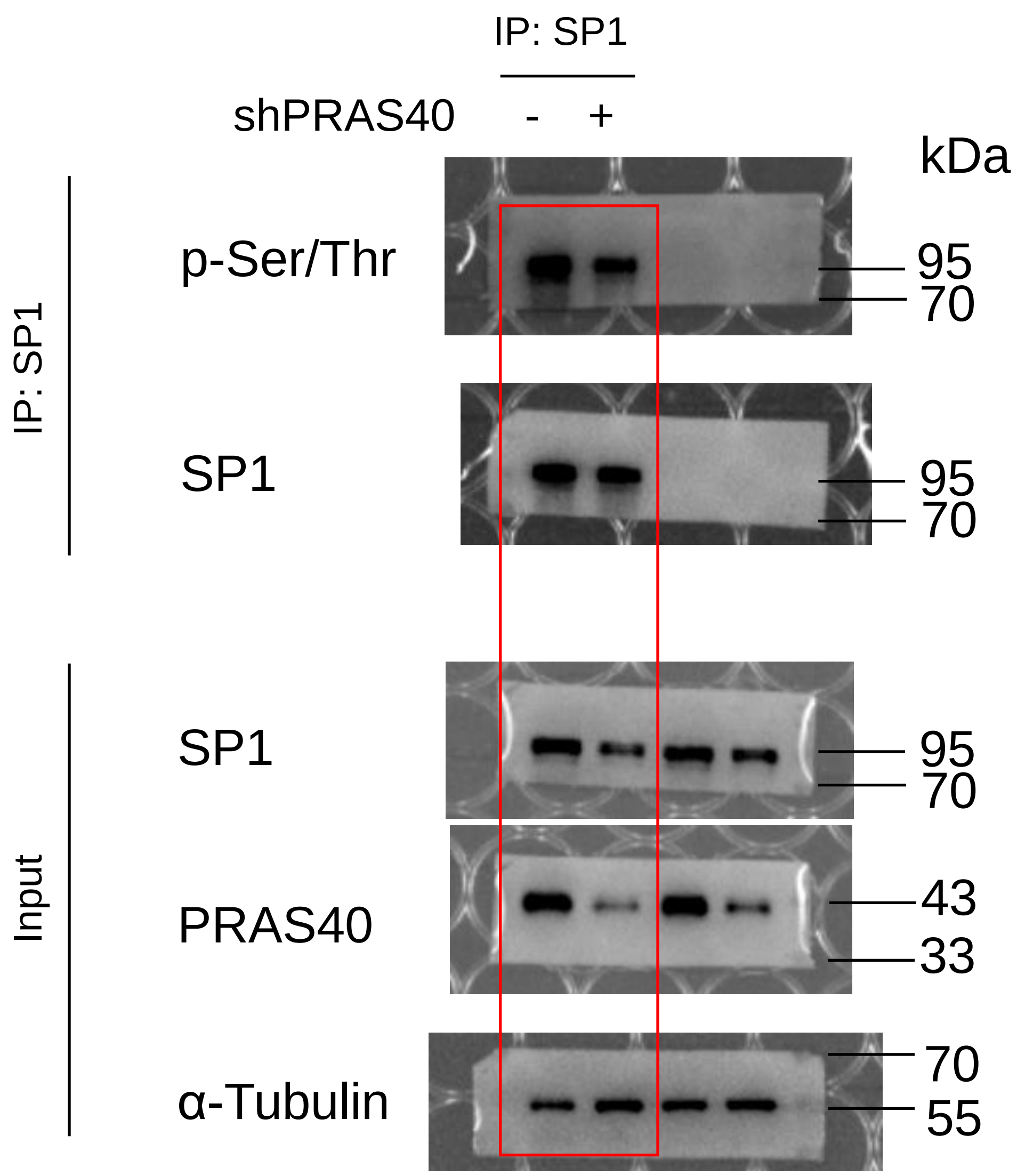

Supplementary Fig. 8AB

Correlated to Fig. 7J

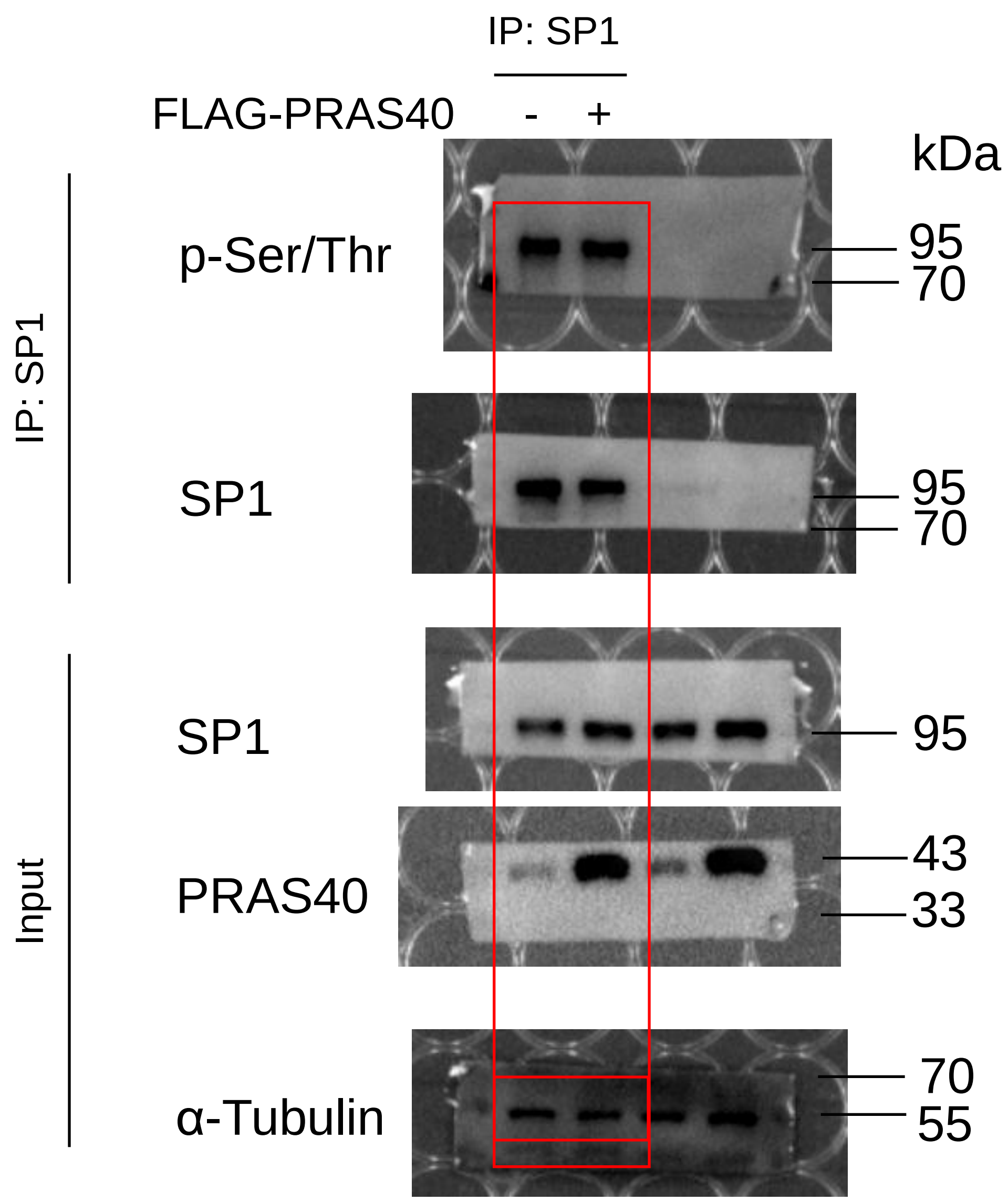

Supplementary Fig. 8AC

Correlated to Fig. 7K

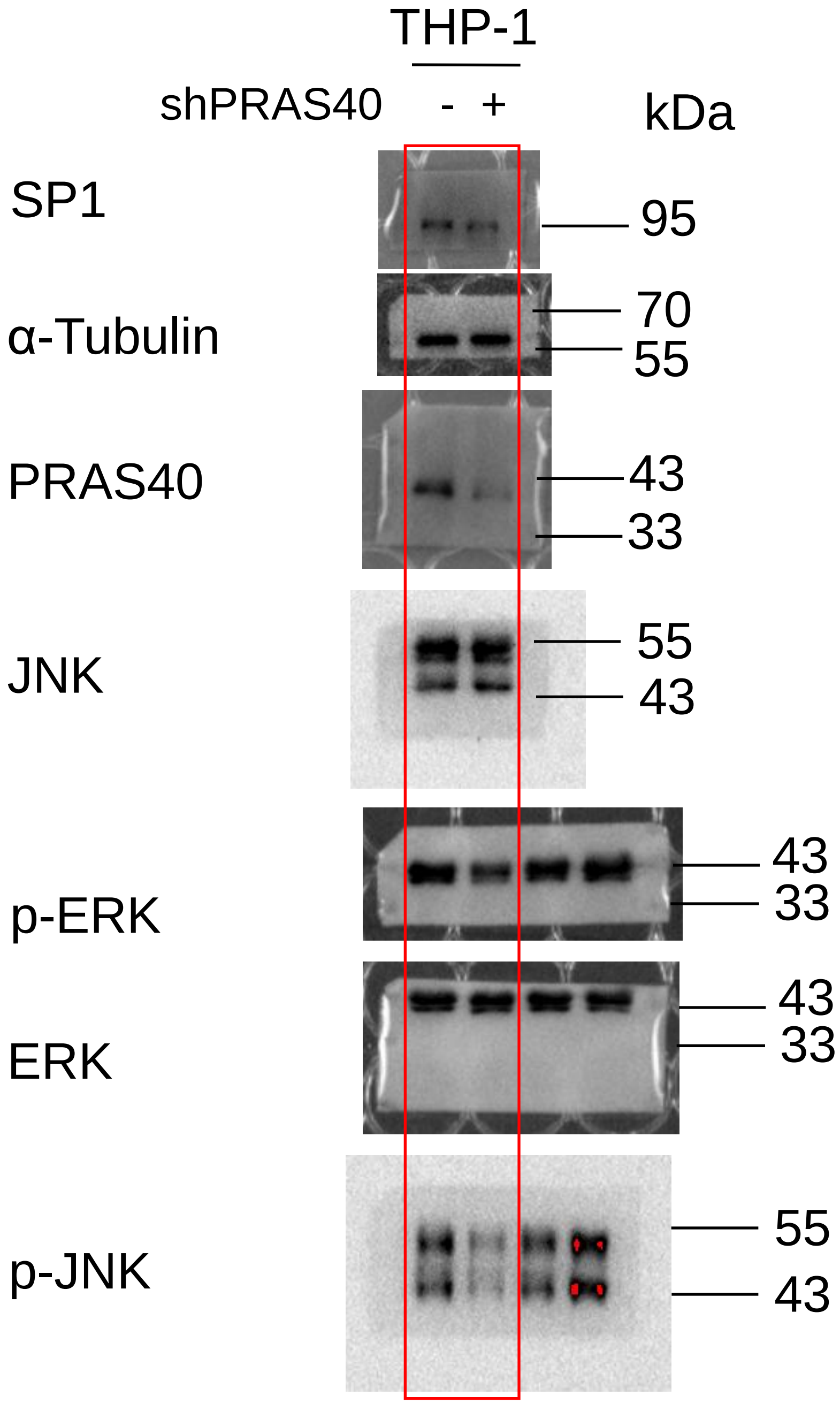

Supplementary Fig. 8AD

Correlated to Fig. 7L

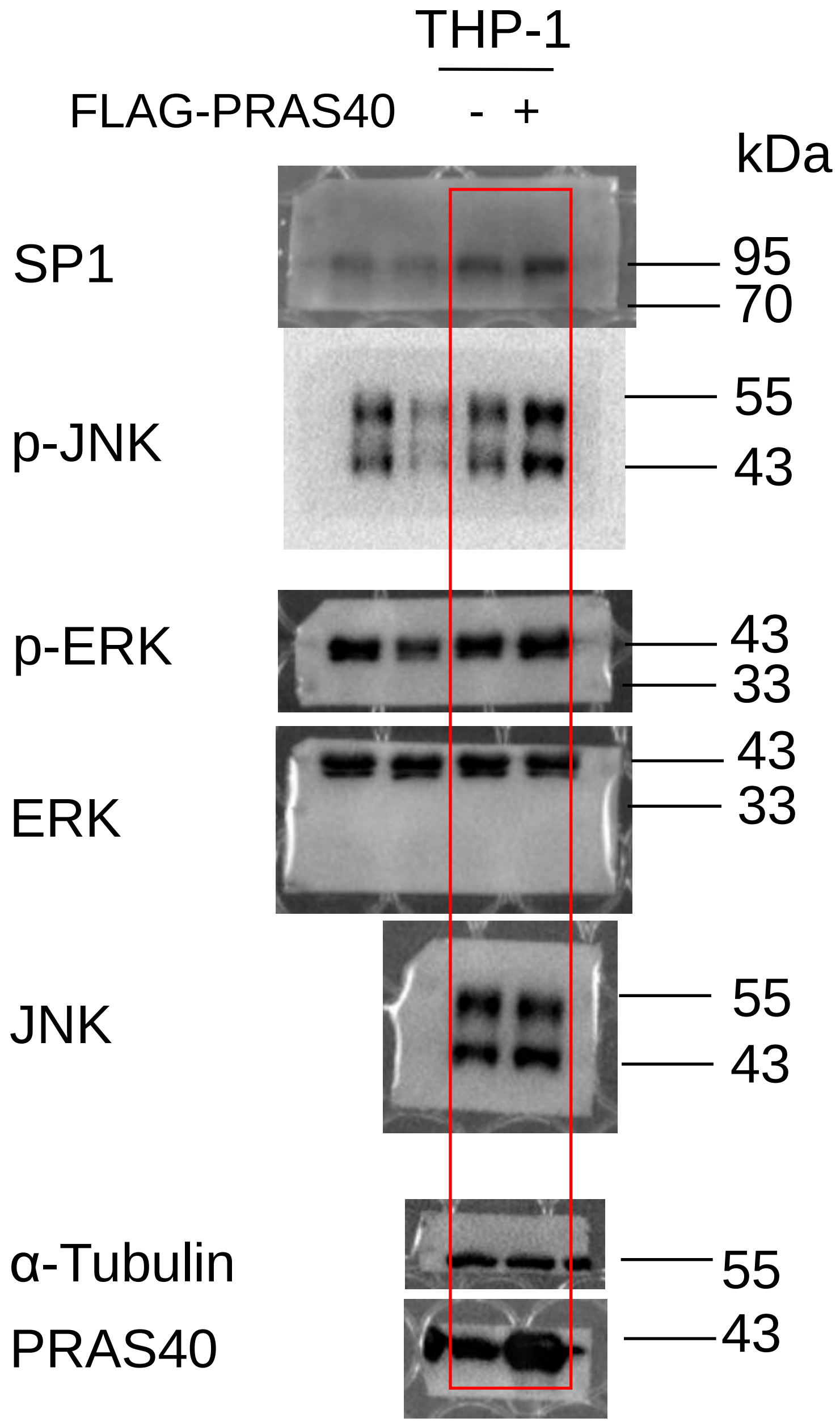

Supplementary Fig. 8AE

Correlated to Fig. 7M

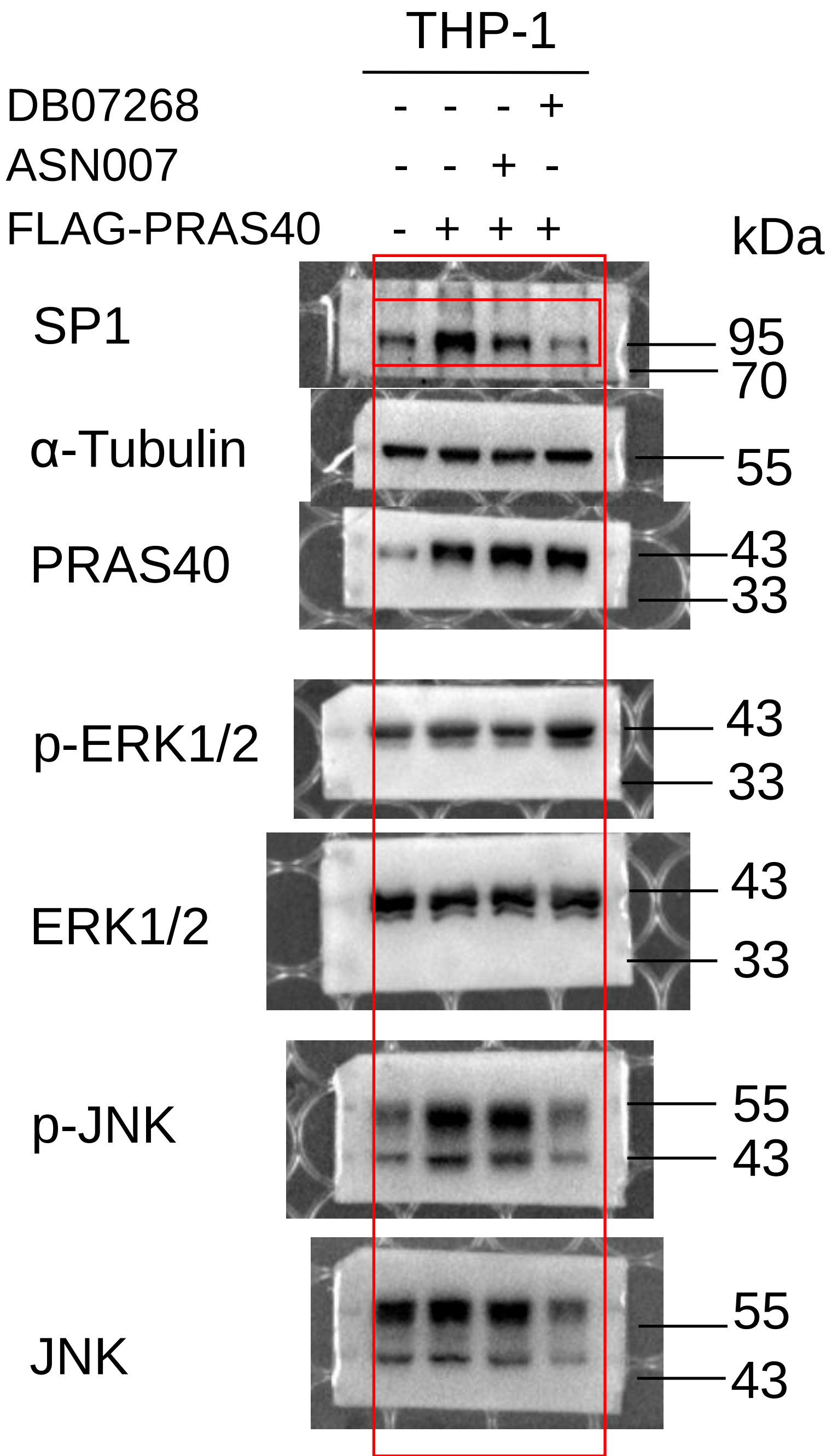

Supplementary Fig. 8AF

Correlated to Fig. 7N

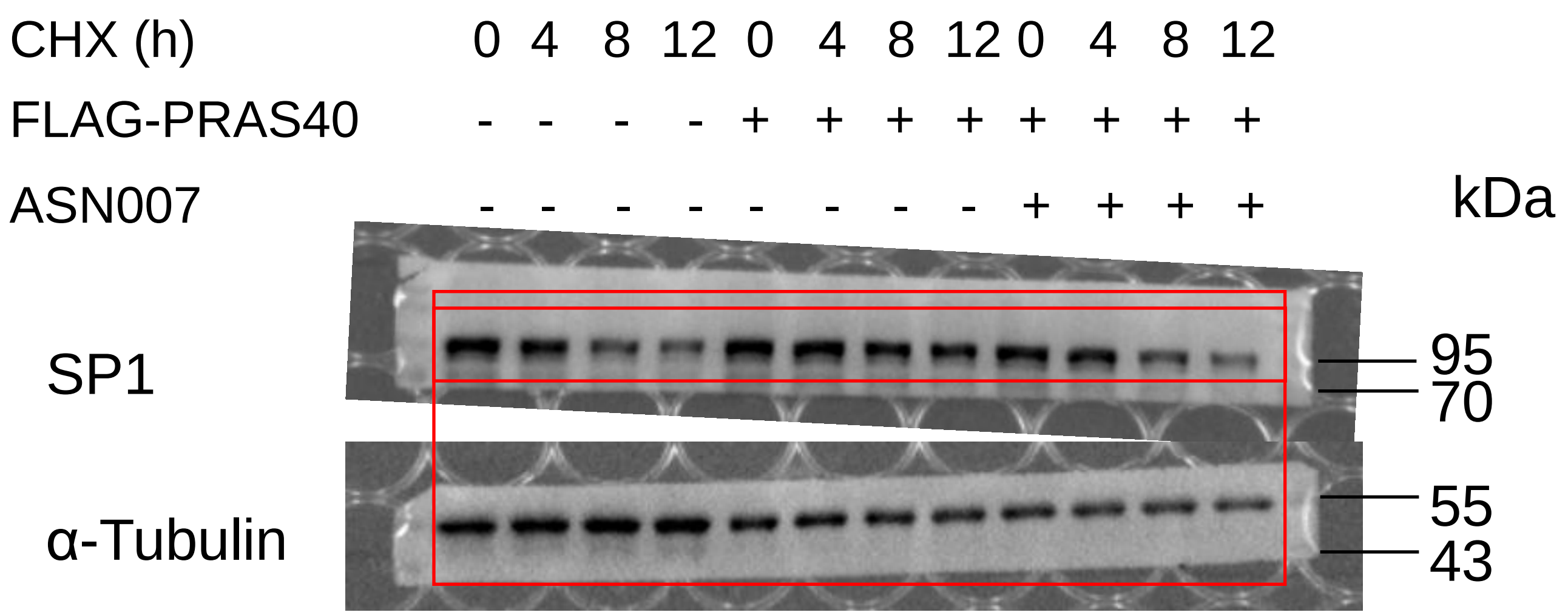

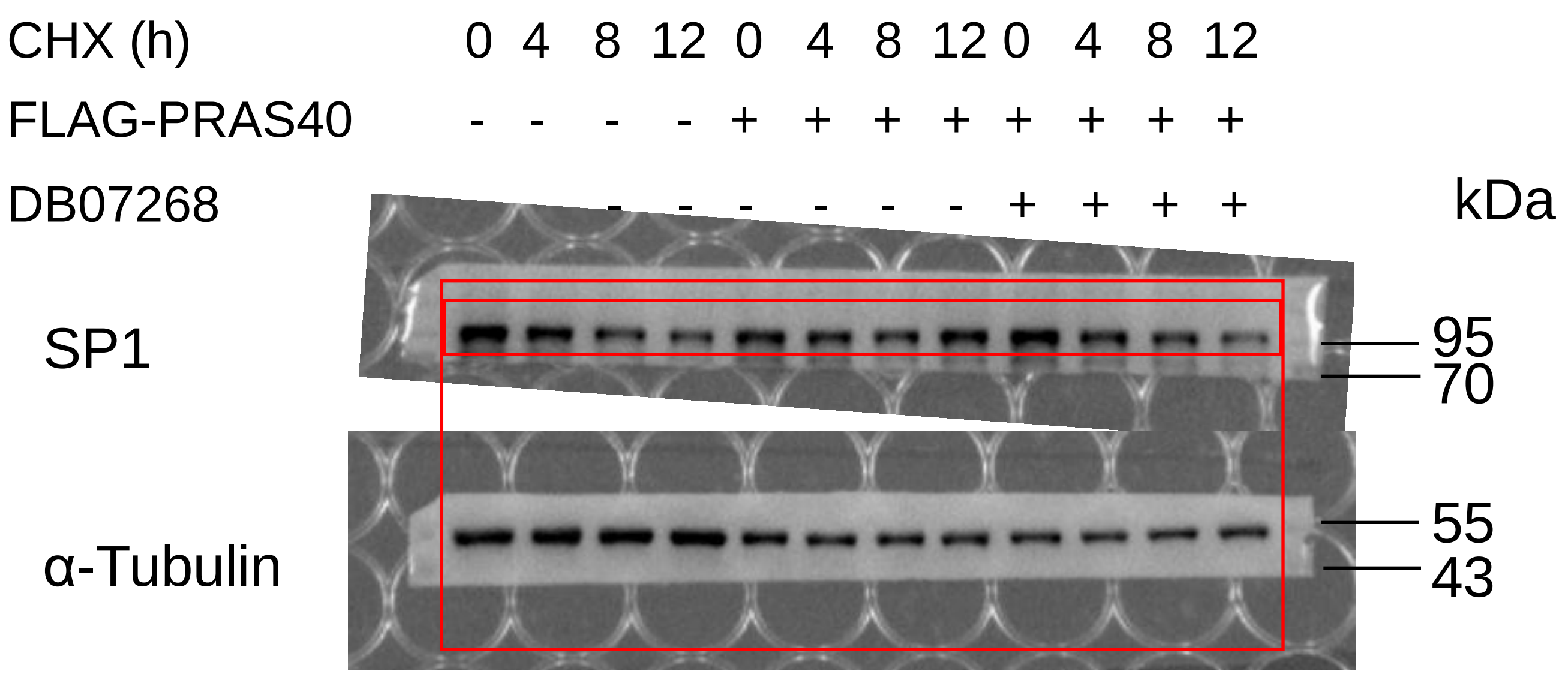

Supplement: Supplementary file 2 [file 41419_2026_8931_MOESM2_ESM.pdf]
